# 1*H*-Indoles from Deoxybenzoin Schiff Bases by Deprotonation—S_N_Ar Cyclization

**DOI:** 10.3390/molecules30193894

**Published:** 2025-09-26

**Authors:** Nash E. Nevels, Richard A. Bunce

**Affiliations:** Department of Chemistry, Oklahoma State University, Stillwater, OK 74078-3071, USA; nnevels@okstate.edu

**Keywords:** 1*H*-indoles, Schiff base deprotonation–S_N_Ar cyclization, heterocycle synthesis, ring formation, annulation

## Abstract

A transition metal-free synthesis of 1,2,5-trisubstituted 1*H*-indoles by a deprotonation–S_N_Ar cyclization sequence from 1-aryl-2-(2-fluoro-5-nitrophenyl)ethan-1-one (deoxy-benzoin) Schiff bases is reported. The starting deoxybenzoins were prepared by Friedel-Crafts acylation of activated aromatic compounds by 2-(2-fluoro-5-nitrophenyl)acetyl chloride with AlCl_3_ or the corresponding acid with (CH_3_SO_2_)_2_O. The Schiff bases were generated by slow distillation of toluene (18–24 h) from a heated solution of each deoxybenzoin (1 equiv) with a benzyl- or phenethylamine, a high-boiling aliphatic amine, or an aniline derivative (5 equiv). Subsequent addition of *N*,*N*-dimethylformamide, 2 equiv of anhydrous K_2_CO_3_, and heating at 90–95 °C for 18–24 h completed the synthesis. Benzyl-, phenethyl-, and high-boiling amines gave excellent yields while the heating requirements for the initial condensation made volatile aliphatic amines difficult to use and gave low yields. Aniline reactivities correlated with substituent-derived base strength, although modified conditions allowed some yields to be improved. Several anticipated competing processes had minimal impact on the outcome of the cyclizations.

## 1. Introduction

The search for new methods to prepare heterocyclic ring systems is a continuing challenge. New approaches require more efficient and environmentally friendly routes to previously inaccessible structures with straightforward procedures, inexpensive reagents and high functional group tolerance. It is with this goal that we have pursued new metal-free strategies terminated by an S_N_Ar cyclization to access various heterocyclic scaffolds [1,2,3].

**Scheme 1:**
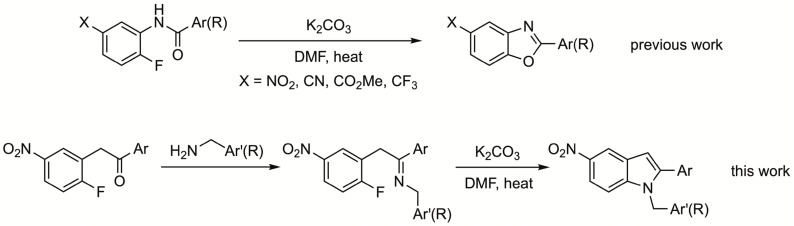
Previous and current strategy for heterocycle closure by intramolecular S_N_Ar reaction.

The current project sought to generate 1,2,5-trisubstituted 1*H*-indoles by deprotonation-cyclization of a Schiff bases derived from a 1,2-diarylethan-1-ones (deoxybenzoins) having an S_N_Ar-activated aryl group at C2. We recently published an analogous approach to benzo[*d*]oxazoles [4] from various *N*-(2-fluoroaryl)anilides, activated by electron-withdrawing groups at C5, and hoped to extend this method to deoxybenzoin substrates to produce 1*H*-indoles (Scheme 1). The requisite anilides in this earlier project were easily synthesized from readily available commercial compounds. The ease of ring closure in *N*,*N*-dimethylformamide (DMF) with 2 equiv of K_2_CO_3_ correlated well with the potency of the activating groups at C5 of the acceptor ring with NO_2_ promoting cyclization at 90 °C, CN at 115 °C, CO_2_Me at 120 °C and CF_3_ at 130 °C. The analogous reaction to prepare 1*H*-indoles from deoxybenzoins, however, proved more difficult than anticipated and required considerable effort to optimize the conversion. Additionally, potential starting materials were limited as structures with C5 electron-withdrawing groups such as CN and CO_2_R were not readily available commercially or through synthesis. Furthermore, accessible substrates with weakly activating substituents, such as CF_3_, failed to give clean conversion to the desired products at the elevated temperatures required. Thus, the current study was restricted to precursors activated only by NO_2_.

**Figure 1:**
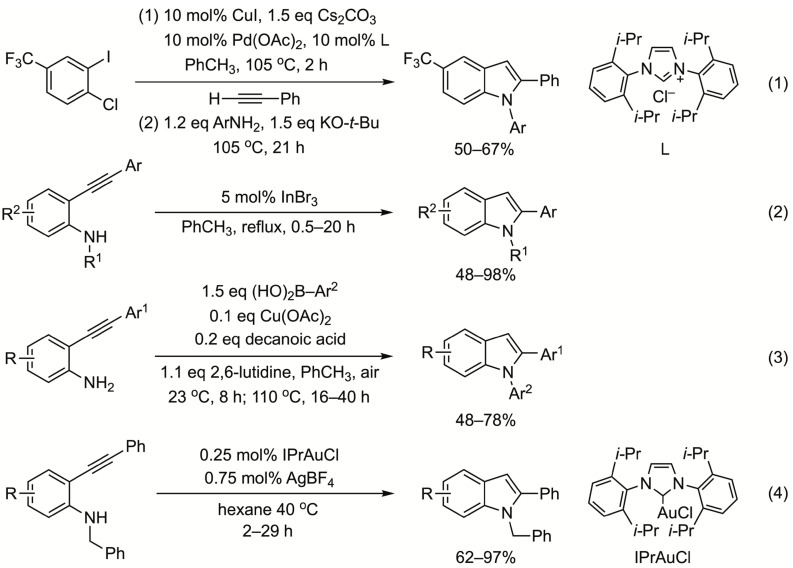
Previous syntheses of 1,2,5-trisubstituted 1*H*-indoles.

Previous syntheses of 1,2-disubstituted 1*H*-indoles have utilized metal-catalyzed cyclizations of 2-alkynylanilines using a modified Larock reaction [5] (Figure 1). Ackermann and Kaspar [6] assembled these heterocycles from *ortho*-dihaloarenes, phenylacetylene, and various anilines using a two-catalyst system involving an *N*-heterocyclic carbene palladium complex and copper(I) iodide ((1) in Figure 1). Sakai and co-workers [7] employed indium(III) bromide in toluene to promote ring closure of *N*-alkyl- or *N*-acyl-2-alkynylanilines to the target compounds ((2) in Figure 1). The Lv group [8] described a one-pot Cu(II)-catalyzed coupling-cyclization of alkynylaniline substrates with aryl or alkyl boronic acids under aerobic conditions to prepare 1,2-disubstituted 1*H*-indoles decorated at C5 by alkyl, halo or cyano groups ((3) in Figure 1). Finally, Michalska and Grela [9] accessed these 1*H*-indole derivatives using a gold-catalyzed hydroamination procedure starting from *N*-alkyl-2-alkynylanilines ((4) in Figure 1). Overall, the number of approaches to 1*H*-indoles with this substitution pattern appears limited and further work is warranted.

**Figure 2:**
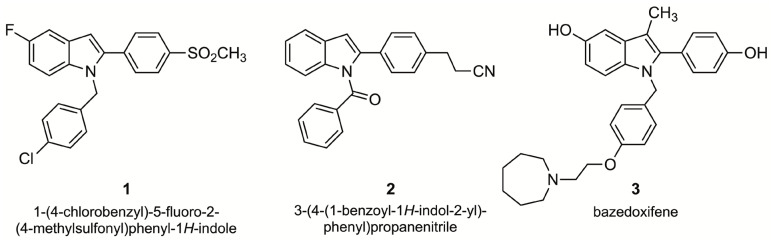
Drug candidates incorporating 1*H*-indoles with 1,2- and 1,2,5-substitution patterns.

1*H*-Indoles are core ring structures in many drug compounds that express a wide range of biological activities [10,11,12,13], although the 1,2,5-trisubstitution pattern is underrepresented. Several examples of compounds potentially available using the current reaction are pictured in Figure 2. 1*H*-Indole **1** has demonstrated significant anti-inflammatory properties through COX-2 inhibition (IC_50_ = 0.15 μM) [14]. Structure **2** is a potent LXRβ agonist (EC_50_ = 12 nM) which raises HDL levels and shows anti-diabetic activity in mice, but its outstanding activity is somewhat offset by undesirable upregulation of SREBP1-c gene expression that leads to lipogenesis in HepG2 cells [15]. Bazedoxifene (**3**), as its acetate salt, has proven effective in the treatment of peri- and postmenopausal osteoporosis [16] and has recently shown promise in the treatment of triple negative breast cancer [16,17].

## 2. Results and Discussion

The syntheses of the deoxybenzoin substrates are illustrated in Table 1. Two variants, based on the Friedel-Crafts acylation reaction were used. In Method A, 2-(2-fluoro-5-nitrophenyl)acetic acid (**4**) was converted to its acid chloride using thionyl chloride in benzene and reacted with an activated aromatic compound in the presence of AlCl_3_ to form 1-aryl-2-(2-fluoro-5-nitrophenyl)ethan-1-ones **5**–**11** [18]. Products generated in this manner were isolated in yields of 70–80%. For Method B, acid **4** was used directly to acylate aromatic precursors in the presence of methanesulfonic anhydride [19]. This approach, which allowed milder conditions, generally afforded slightly lower yields (50–70%), except in the case of 1,4-benzodioxane, which afforded the deoxybenzoin in 85% yield. As for all Friedel-Crafts acylations, success was observed primarily with electron-rich aromatic structures, although halogenated rings were also successfully reacted. Other deoxybenzoins bearing C5 S_N_Ar activating groups, such as CN and CO_2_R, have not been reported previously and are not commercially available. One other precursor, activated by CF_3_, was prepared from commercial 2-(2-fluoro-5-(trifluoromethyl)phenyl)acetic acid, but the temperature required for the final cyclization (>130 °C) resulted in significant decomposition of the substrate.

The sequence began with the formation of the Schiff base by condensation of the deoxybenzoin with a primary amine. Only primary amines were used since two bonds to the nitrogen must be formed in the reaction. Complete conversion to the Schiff base was found to be problematic, presumably due to steric and electronic factors. Reaction progress was monitored by thin layer chromatography (TLC). Several reaction solvents–EtOH, DMF, benzene, and toluene–were evaluated, with the latter providing the best results. Trials with toluene using catalytic HCl and triethylamine as well as molecular sieves to remove the water afforded 1*H*-indoles in less than 25% isolated yields. The optimized procedure involved the use of 1 equiv of the deoxybenzoin with 5 equiv of the amine (2 or 3 equiv proved insufficient) and slow distillation of toluene from the reaction mixture at 120–125 °C during 18–24 h. Following removal of the toluene, the solvent was replaced with DMF, 2 equiv of anhydrous K_2_CO_3_ was added, and the mixture was heated at 90–95 °C for 18–24 h. As in most of our work to prepare heterocycles by S_N_Ar cyclizations, 2 equiv of K_2_CO_3_ was sufficient for complete conversion; 1 equiv of base gave lower yields while 2.5 or 3 equiv offered no improvement.

The results of our study are summarized in Table 2 and spectra for all 1*H*-indoles can be found in the Supplementary Information. Benzylamines (entries 1, 2, 3, 11, 12, 14, 15, 16, 17, 20, 21, 24, 27, 28, 30 and 31; yields 55–86%) and phenethylamines (entries 4, 13, 18, 22, 26, 29 and 32; yields 62–86%) gave the best results as they were nonvolatile and not subject to electronic effects exerted by substituents on the aromatic ring. For high-boiling aliphatic amines (entry 5), a mixture of the deoxybenzoin and the amine (1:5 equiv) was treated in a similar manner to give the 1*H*-indole in excellent yield (77%). A larger excess (10 equiv) was necessary for volatile aliphatic amines (entries 6, 19 and 23), but the yields were lower (32–58%) due to inefficient condensation with the deoxybenzoin at low temperature and co-distillation of the amine with toluene. For aniline derivatives (entries 7, 8, 9 and 10), the results correlated with substituent-derived electronic effects that enhanced or diminished the basicity/nucleophilicity of the nitrogen. For the current study, we used a series of four anilines, listed from strongest to weakest base: 4-methoxyaniline (pK_b_ = 8.7), aniline (pK_b_ = 9.4), 4-fluoroaniline (pK_b_ = 9.5) and 4-(trifluoromethyl)aniline (pK_b_ = 11.4) [20]. Under standard conditions with 5 equiv of the amine, it was found that 4-methoxyaniline afforded a very high yield of the 1*H*-indole product (84%), aniline and 4-fluoroaniline gave poor yields (23% and 9%, respectively), and 4-(trifluoromethyl)aniline produced no isolable product. Yields for aniline and 4-fluoroaniline were increased to 42% and 82% by using 10 equiv of the aniline and performing the final ring closure at 120–125 °C. Although the requirement for a large excess of the amine is unclear, the dramatic increase in the yield of 1*H*-indole from 4-fluoroaniline likely results from resonance electron donation to the para position of the fluorinated aromatic system [21,22]. This phenomenon is known to activate these systems toward electrophilic aromatic substitution and should also increase the basicity of the C4 nitrogen in this compound. These observations suggest that formation of the Schiff base is the critical step of the sequence since stronger bases should be more reactive toward the deoxybenzoin carbonyl. It also demonstrates that other factors, beyond the basicity assessed from pK_b_ values, can have a major impact on the reaction outcome.

Interestingly, two potential competing S_N_Ar reactions were not observed in our study. In deoxybenzoins where C1 was substituted by a 4-fluoro- or 4-chloroarene (*viz*. **8**–**10**), no addition was observed at the halogenated site despite modest activation by the C1 carbonyl. Additionally, an alternative cyclization involving initial addition of the amine to the 2-fluoro-5-nitrophenyl ring, followed by condensation of the newly added nitrogen at the C1 carbonyl was not observed. This process was expected to compete, although the attenuated nucleophilicity of the resulting aniline should prevent closure to the 1*H*-indole, and none of this adduct was observed. A third side reaction anticipated was deprotonation to give the deoxybenzoin enolate and ring closure from oxygen to give to the benzofuran product. While this process presumably occurs to a small extent in all the reactions, it was only significant and easily separable from the reaction of 4-fluoroaniline (5 equiv scale) when slow heating of the initial condensation from 80 to 125 °C yielded 5-nitro-2-phenylbenzofuran in 25% yield. At lower temperatures, the weakly basic aniline nitrogen was unable to effectively attack the carbonyl but instead removed an acidic methylene proton from the deoxybenzoin leading to cyclization through the conjugated carbonyl oxygen. The pK_a_ of unsubstituted deoxybenzoin in DMSO is 17.7 [23] and undoubtedly lower for the nitroarene-bearing derivative. Thus, it is surprising that this reaction was less favorable than carbonyl addition at 125 °C using more basic/nucleophilic amines. The weakest base in the current study, 4-(trifluoromethyl)aniline, underwent significant decomposition and afforded no product from either pathway.

Information on the structures of the products was derived primarily from the ^1^H NMR spectra. The products showed a typical 1,3,4-substitution pattern for the NO_2_ substituted ring of the 1*H*-indole: a downfield doublet (*J* = 2.2 Hz) at *ca* δ 8.6 for the C4 proton ortho to NO_2_, a doublet of doublets (*J* = 9.0, 2.2 Hz) at *ca* δ 8.1 for the C6 proton ortho to the NO_2_, and a doublet (*J* = 9.0 Hz) between δ 7.4–7.1 for the C7 proton. Para-disubstituted aromatic substituents at C2 displayed a distinctive AA’BB’ pattern in the aromatic region of the spectrum and the C3 proton presented as a singlet or small doublet (*J* = 0.8 Hz) between δ 6.5–6.9. For the benzylamine-derived 1*H*-indoles, the methylene group appeared as a singlet at *ca* δ 5.4, while the phenethylamine-derived 1*H*-indoles displayed two triplets at *ca* δ 4.4 and δ 2.9 for the coupled methylene protons. Products derived from aliphatic amines exhibited predictable multiplicities and chemical shifts for hydrogens on the N1-substituted carbons as well as first order patterns for the remaining protons. ^13^C and ^19^F NMR were somewhat less useful for structure elucidation but did provide useful information on the numbers and types of carbons and fluorines, respectively.

**Scheme 2:**
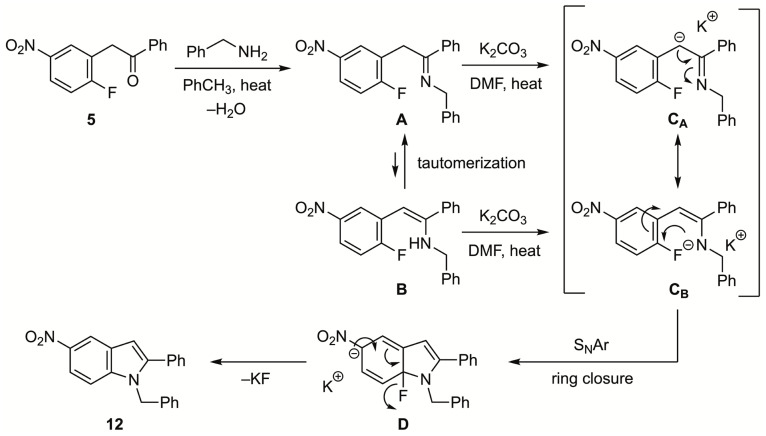
Plausible mechanism for formation of 1*H*-indole **12** from deoxybenzoin **5** and benzylamine.

A plausible mechanism to convert deoxybenzoins to 1*H*-indoles is depicted in Scheme 2 for the reaction of **5** with benzylamine to give **12**. The initial step involves condensation of the amine with the carbonyl group of **5** to give the Schiff base. Although the imine tautomer **A** of this species is generally more stable than the enamine tautomer **B** [24], the reaction must proceed through an enamine-like intermediate **C_B_**, and thus, it is highly probable that **B** is involved to some extent as the reaction progresses [25]. Treatment of the Schiff base **A** with anhydrous K_2_CO_3_ in DMF would deprotonate the carbon α to the imine to give **C_A_**. The highly conjugated delocalized anion **C** would then close from **C_B_** on the fluorine-bearing carbon to give the Meisenheimer complex **D** and rearomatize by loss of KF to give the 1*H*-indole **12**. Deprotonation of the enamine nitrogen in **B** (if present) would be directly close to the final product via **C_B_**. Although our reactions were performed under an atmosphere of dry nitrogen, it was found that these operations were not sensitive to oxygen and high yields were achieved even when the flask was briefly opened to air during the replacement of toluene with DMF.

## 3. Materials and Methods

### 3.1. General Methods

Unless otherwise indicated, all reactions were performed under N_2_ in dry glassware. All commercial reagents and solvents were used as received. Reactions were followed by TLC on silica gel GF plates (Analtech No 21521, Newark, DE, USA). Preparative separations were performed on Davisil^®^ grade 62, 70–200 mesh silica gel (Fisher Scientific, Pittsburgh, PA, USA) containing 0.5% of UV-05 phosphor (Sorbent Technologies, Norcross, GA, USA) slurry packed into quartz columns. Band elution for all chromatographic separations was detected using a hand-held 254-nm UV light source (Fisher Scientific, Pittsburgh, PA, USA). Melting points (uncorrected) were obtained using a MEL–TEMP apparatus (Cambridge, MA, USA). FT–IR spectra were run using an Agilent Cary 630 spectrometer (Santa Clara, CA, USA) fitted with an ATR sampling module. ^1^H and ^13^C NMR spectra were measured using a Bruker Avance 400 system (Billerica, MA, USA) at 400 MHz and 101 MHz, respectively, in CDCl_3_ (Cambridge Isotope, Andover, MA, USA) containing 0.05% *v*/*v* tetramethylsilane as the internal standard. Chemical shifts are given in δ (ppm) units relative to the standard and coupling constants (*J*) are given in Hz. ^19^F NMR spectra were collected at 376 MHz on the same instrument and are referenced to internal C_6_H_5_F at δ -113.15. Low-resolution mass spectra were obtained using a GC–MS instrument (Hewlett-Packard Model 1800A GCD, Palo Alto, CA, USA). Elemental analyses (±0.4%) on all new compounds were determined by Atlantic Microlabs, Inc. (Norcross, GA, USA).

### 3.2. Preparation of the 1-Aryl-2-(2-fluoro-5-nitrophenyl)lethan-1-one (Deoxybenzoin) Substrates

Method A: Friedel-Crafts reaction using AlCl_3_ [1]. The acid chloride of 2-(2-fluoro-5-nitrophenyl)acetic acid (**4**) was generated by dissolving the acid (2.00 g, 10.5 mmol, 1 equiv) in benzene, adding thionyl chloride (1.50 g, 0.92 mL, 12.6 mmol, 1.2 equiv) and refluxing for 2 h. The benzene was removed under vacuum and replaced with the aromatic compound to be substituted (5–10 mL). AlCl_3_ (1.1 equiv) was cautiously added, and the reaction was heated under reflux until TLC (25% EtOAc in hexane) indicated complete consumption of the acid chloride (2–3 h). The crude reaction mixture was added to concentrated HCl (20 mL) in ice (100 g). The mixture was extracted with EtOAc (2 × 15 mL) and the combined organic extracts were washed with saturated aqueous NaCl (1 × 25 mL), saturated aqueous NaHCO_3_ (1 × 25 mL) and additional NaCl solution (2 × 25 mL). After drying (MgSO_4_), removal of the EtOAc under vacuum and recrystallization from absolute ethanol, the deoxybenzoin derivative was isolated as a tan solid. The compounds prepared using this method are indicated below.

Method B: Friedel-Crafts reaction using methanesulfonic anhydride [2]. A solution of acid **4** (2.00 g, 10.5 mmol, 1 equiv), methanesulfonic anhydride (1.3–1.5 equiv) and the aromatic substrate (1.3–1.5 equiv) in benzene (or a less reactive aromatic solvent) was heated at 75–100 °C until TLC (50% EtOAc in hexane) indicated complete consumption of the acid (2–10 h). The reaction was boiled with 90% EtOH for 5 min, then cooled, added to saturated aqueous NaCl (50 mL), and extracted with EtOAc (2 × 25 mL). The remainder of the workup and purification scheme was as reported for Method A. The compounds prepared using this method are indicated below.

#### 3.2.1. 2-(2-Fluoro-5-nitrophenyl)-1-phenylethan-1-one (**5**)

Method A; reaction of **4** with benzene gave 2.13 g of **5** (82%) as a tan solid, mp 80–82 °C; IR: 1680, 1531, 1343 cm^−1^; ^1^H NMR (400 MHz, CDCl_3_): δ 8.24–8.19 (complex, 2H), 8.05 (m, 2H), 7.64 (tt, *J* = 7.4, 1.8 Hz, 1H), 7.53 (t, *J* = 8.0 Hz, 2H), 7.24 (t, *J* = 8.6 Hz, 1H), 4.44 (d, *J* = 1.5 Hz, 2H); ^13^C {^1^H} NMR (101 MHz, CDCl_3_): δ 194.5, 164.8 (d, *J* = 257.4 Hz), 144.2, 135.9, 133.9, 128.9, 128.5, 127.9 (d, *J* = 6.5 Hz), 125.0 (d, *J* = 10.2 Hz), 123.8 (d, *J* = 18.5 Hz), 116.3 (d, *J* = 24.9 Hz), 38.6 (d, *J* = 1.5 Hz); these NMR data matched those reported in the literature [26]; ^19^F {^1^H} NMR (376 MHz, CDCl_3_ referenced to C_6_H_5_F): δ -105.9; MS, *m*/*z* for C_14_H_10_FNO_3_: 259; Anal. Calcd for C_14_H_10_FNO_3_: C, 64.87; H, 3.89; N, 5.40. Found: C, 64.68; H, 3.94; N, 5.35.

#### 3.2.2. 2-(2-Fluoro-5-nitrophenyl)-1-(4-methylphenyl)ethan-1-one (**6**)

Methods A and B; reaction of **4** with toluene gave 2.19 g of **6** (80% from A) and 1.70 g (62% from B) as a tan solid, mp 97–98 °C; IR: 1676, 1523, 1344 cm^−1^; ^1^H NMR (400 MHz, CDCl_3_): δ 8.22–8.18 (complex, 2H), 7.94 (d, *J* = 8.2 Hz, 2H), 7.32 (d, *J* = 8.2 Hz, 2H), 7.23 (t, *J* = 8.6 Hz, 1H), 4.40 (d, *J* = 1.3 Hz, 2H), 2.44 (s, 3H); ^13^C {^1^H} NMR (101 MHz, CDCl_3_): δ 194.2, 164.8 (d, *J* = 257.3 Hz), 144.8, 144.2, 138.5, 129.6, 128.4, 127.9 (d, *J* = 6.4 Hz), 125.0 (d, *J* = 10.3 Hz), 124.0 (d, *J* = 18.5 Hz), 116.3 (d, *J* = 24.9 Hz), 38.5 (d, *J* = 1.5 Hz), 21.7; ^19^F {^1^H} NMR (376 MHz, CDCl_3_ referenced to C_6_H_5_F): δ -105.9; MS, *m*/*z* for C_15_H_12_FNO_3_: 273; Anal. Calcd for C_15_H_12_FNO_3_: C, 65.93; H, 4.43; N, 5.13. Found: C, 65.96; H, 4.42; N, 5.07.

#### 3.2.3. 2-(2-Fluoro-5-nitrophenyl)-1-(4-methoxyphenyl)ethan-1-one (**7**)

Method A; reaction of **4** with anisole gave 2.26 g of **7** (78%) as a tan solid, mp 100–101 °C; IR: 2844, 1663, 1524, 1348 cm^−1^; ^1^H NMR (400 MHz, CDCl_3_): δ 8.22–8.19 (complex, 2H), 8.02 (d, *J* = 8.9 Hz, 2H), 7.23 (t, *J* = 8.8 Hz, 1H), 6.99 (d, *J* = 8.9 Hz, 2H), 4.38 (d, *J* = 1.5 Hz, 2H), 3.90 (s, 3H); ^13^C {^1^H} NMR (101 MHz, CDCl_3_): δ 193.0, 164.7 (d, *J* = 257.1 Hz), 164.1, 144.2, 130.7, 129.0, 127.9 (d, *J* = 6.5 Hz), 124.9 (d, *J* = 10.2 Hz), 124.2 (d, *J* = 18.5 Hz), 116.2 (d, *J* = 25.0 Hz), 114.1, 55.6, 38.2 (d, *J* = 1.5 Hz); ^19^F {^1^H} NMR (376 MHz, CDCl_3_ referenced to C_6_H_5_F): δ -106.0; MS, *m*/*z* for C_15_H_12_FNO_4_: 289; Anal. Calcd for C_15_H_12_FNO_4_: C, 62.28; H, 4.18; N, 4.84. Found: C, 62.32; H, 4.15; N, 4.76.

#### 3.2.4. 2-(2-Fluoro-5-nitrophenyl)-1-(4-fluorophenyl)ethan-1-one (**8**)

Method A; reaction of **4** with fluorobenzene gave 2.17 g of **8** (78%) as a tan solid, mp 101–103 °C; IR: 1672, 1524, 1338 cm^−1^; ^1^H NMR (400 MHz, CDCl_3_): δ 8.24–8.18 (complex, 2H), 8.10–8.05 (complex, 2H), 7.27–7.17 (complex, 3H), 4.40 (d, *J* = 0.8 Hz, 2H); ^13^C {^1^H} NMR (101 MHz, CDCl_3_): δ 192.9, 166.2 (d, *J* = 256.2 Hz), 164.7 (d, *J* = 257.2 Hz), 144.2. 132.4 (d, *J* = 3.0 Hz), 131.0 (d, *J* = 9.4 Hz), 127.9 (d, *J* = 6.3 Hz), 125.0 (d, *J* = 10.2 Hz), 123.5 (d, *J* = 18.4 Hz), 116.3 (d, *J* = 24.8 Hz), 116.1 (d, *J* = 22.0 Hz), 38.5 (d, *J* = 1.5 Hz); ^19^F {^1^H} NMR (376 MHz, CDCl_3_ referenced to C_6_H_5_F): δ -103.7, -106.0; MS, *m*/*z* for C_14_H_9_F_2_NO_3_: 277; Anal. Calcd for C_14_H_9_F_2_NO_3_: C, 60.66; H, 3.27; N, 5.05. Found: C, 60.52 H, 3.24; N, 5.01.

#### 3.2.5. 1-(4-Fluoro-3-methylphenyl)-2-(2-fluoro-5-nitrophenyl)ethan-1-one (**9**)

Method B; reaction of **4** with 2-fluorotoluene gave 1.90 g of **9** (65%) as a tan solid, mp 105–107 °C; IR: 1675, 1524, 1331 cm^−1^; ^1^H NMR (400 MHz, CDCl_3_): δ 8.24–8.18 (complex, 2H), 7.93–7.86 (complex, 2H), 7.24 (t, *J* = 8.8 Hz, 1H), 7.13 (t, *J* = 8.8 Hz, 1H), 4.39 (s, 2H), 2.36 (d, *J* = 2.0 Hz, 3H); ^13^C {^1^H} NMR (101 MHz, CDCl_3_): δ 193.2, 164.8 (d, *J* = 254.8 Hz), 164.7 (d, *J* = 257.2 Hz), 144.2 (d, *J* = 2.6 Hz), 132.2 (d, *J* = 6.7 Hz), 132.1 (d, *J* = 3.3 Hz), 128.3 (d, *J* = 9.5 Hz), 127.9 (d, *J* = 6.4 Hz), 125.9 (d, *J* = 19.0 Hz), 125.1 (d, *J* = 10.2 Hz), 123.7 (d, *J* = 18.5 Hz), 116.3 (d, *J* = 24.9 Hz), 115.6 (d, *J* = 23.2 Hz), 38.5 (d, *J* = 1.3 Hz), 14.6 (d, *J* = 3.5 Hz); ^19^F {^1^H} NMR (376 MHz, CDCl_3_ referenced to C_6_H_5_F): δ -106.0, -107.9; MS, *m*/*z* for C_15_H_11_F_2_NO_3_: 291; Anal. Calcd for C_15_H_11_F_2_NO_3_: C, 61.86; H, 3.81; N, 4.81. Found: C, 61.79; H, 3.78; N, 4.74.

#### 3.2.6. 1-(4-Chlorophenyl)-2-(2-fluoro-5-nitrophenyl)ethan-1-one (**10**)

Method A; reaction of **4** with chlorobenzene gave 1.88 g of **10** (64%) as a tan solid, mp 87–89 °C; IR: 1680, 1523, 1334 cm^−1^; ^1^H NMR (400 MHz, CDCl_3_): δ 8.24–8.18 (complex, 2H), 7.98 (d, *J* = 8.6 Hz, 2H), 7.49 (d, *J* = 8.6 Hz, 2H), 7.24 (t, *J* = 8.7 Hz, 1H), 4.40 (d, *J* = 1.3 Hz, 2H); ^13^C {^1^H} NMR (101 MHz, CDCl_3_): δ 193.4, 164.7 (d, *J* = 257.2 Hz), 144.2, 140.4, 134.2, 129.7, 129.3, 127.9 (d, *J* = 6.3 Hz), 125.2 (d, *J* = 10.2 Hz), 123.4 (d, *J* = 18.4 Hz), 116.3 (d, *J* = 24.8 Hz), 38.6 (d, *J* = 1.5 Hz); ^19^F {^1^H} NMR (376 MHz, CDCl_3_ referenced to C_6_H_5_F): δ -105.9; MS, *m*/*z* for C_14_H_9_ClFNO_3_: 293, 295 (*ca*. 3:1); Anal. Calcd for C_14_H_9_ClFNO_3_: C, 57.26; H, 3.09; N, 4.77. Found: C, 57.29; H, 3.05; N, 4.71.

#### 3.2.7. 1-(2,3-Dihydrobenzo[b][1,4]dioxin-6-yl)-2-(2-fluoro-5-nitrophenyl)ethan-1-one (**11**)

Method B; reaction of **4** with 1,4-benzodioxane gave 2.71 g of **11** (85%) as a tan solid, mp 137–138 °C; IR: 1670, 1524, 1343 cm^−1^; ^1^H NMR (400 MHz, CDCl_3_): δ 8.22–8.17 (complex, 2H), 7.59–7.56 (complex, 2H), 7.22 (t, *J* = 8.7 Hz, 1H), 6.96 (d, *J* = 9.0 Hz, 1H), 4.36–4.33 (complex, 4H), 4.30 (m, 2H); ^13^C {^1^H} NMR (101 MHz, CDCl_3_): δ 192.9, 164.7 (d, *J* = 257.3 Hz), 148.7, 144.8, 143.6, 129.7, 127.9 (d, *J* = 6.5 Hz), 124.9 (d, *J* = 10.2 Hz), 124.1 (d, *J* = 18.6 Hz), 122.5, 117.9, 117.5, 116.2 (d, *J* = 24.9 Hz), 64.8, 64.1, 38.2 (d, *J* = 1.5 Hz); ^19^F {^1^H} NMR (376 MHz, CDCl_3_ referenced to C_6_H_5_F): δ -105.9; MS, *m*/*z* for C_16_H_12_FNO_5_: 317; Anal. Calcd for C_16_H_12_FNO_5_: C, 60.57; H, 3.81; N, 4.41. Found: C, 60.43; H, 3.79; N, 4.33.

### 3.3. Representative Procedure to Prepare 1H-Indoles

Method C: A solution of the deoxybenzoin (0.1 g, 1.0 equiv) and the benzyl- or phenethylamine, the high-boiling aliphatic amine or the aniline derivative (5 equiv) in toluene (8 mL) was heated in a silicone oil bath at 120–125 °C for 18–24 h with slow distillation of the toluene. [Note: In addition to TLC indicating reaction was complete, droplets of water were observed in the condenser after all the toluene was removed.] The last traces of toluene and excess amine were carefully removed under high vacuum. The resulting residue, in each case, was dissolved in DMF (4 mL), anhydrous K_2_CO_3_ (2 equiv) was added, and the mixture was stirred at 90–95 °C for 18–24 h. The crude reaction mixture was added to saturated aqueous NaCl (25 mL) and extracted with EtOAc (2 × 20 mL). The combined organic extracts were washed with saturated aqueous NaCl (3 × 20 mL) and then dried (MgSO_4_) and concentrated onto silica gel (2–3 g) under vacuum. The silica was added to the top of a 40 cm × 2 cm silica gel column and eluted with 2–5% EtOAc in hexane to remove unreacted amine and any benzofuran product. This was followed by elution with 10–25% EtOAc in hexane to give the 1*H*-indole product. Concentration of the eluent under vacuum gave a light yellow solid or oil which crystallized upon addition of 5% ether in hexane. Filtration of the solid gave the following 1*H*-indoles.

Method D: For volatile amines and two of the anilines, a mixture of the deoxybenzoin and the amine (1:10 equiv, respectively) were heated at the indicated temperature. Workup and purification were as described for Method C.

#### 3.3.1. 1-Benzyl-5-nitro-2-phenyl-1*H*-indole (**12**)

Method C: Deoxybenzoin **5** (1 equiv) with benzylamine (5 equiv) gave indole **12** (101 mg, 80%) as a yellow solid, mp 123–124 °C; IR: 1523, 1344 cm^−1^; ^1^H NMR (400 MHz, CDCl_3_): δ 8.62 (d, *J* = 2.2 Hz, 1H), 8.05 (dd, *J* = 9.0, 2.2 Hz, 1H), 7.43 (s, 5H), 7.32–7.24 (complex, 3H), 7.20 (d, *J* = 9.0 Hz, 1H), 6.97 (m, 2H), 6.80 (d, *J* = 0.8 Hz, 1H), 5.41 (s, 2H); ^13^C {^1^H} NMR (101 MHz, CDCl_3_): δ 145.0, 142.1, 140.6, 136.9, 131.4, 129.3, 129.0, 128.9, 128.8, 127.7, 127.5, 125.8, 117.7, 117.5, 109.4, 104.4, 48.1; these NMR data matched those reported in the literature [27]; MS (*m*/*z*): 328; Anal. Calcd for C_21_H_16_N_2_O_2_: C, 76.81; H, 4.91; N, 8.53; Found: C, 76.57; H, 4.89; N, 8.48.

#### 3.3.2. 1-(3-Methoxybenzyl)-5-nitro-2-phenyl-1*H*-indole (**13**)

Method C: Deoxybenzoin **5** (1 equiv) with 3-methoxybenzylamine (5 equiv) gave indole **13** (114 mg, 83%) as a yellow solid, mp 126–127 °C; IR: 2838, 1520, 1336 cm^−1^; ^1^H NMR (400 MHz, CDCl_3_): δ 8.62 (d, *J* = 2.2 Hz, 1H), 8.06 (dd, *J* = 9.0, 2.2 Hz, 1H), 7.46–7.42 (complex, 5H), 7.21 (m, 2H), 6.80 (coincident m, 1H and d, *J* = 0.8 Hz, 1H), 6.57 (d, *J* = 7.6 Hz, 1H), 6.51 (s, 1H) 5.37 (s, 2H), 3.71 (s, 3H); ^13^C {^1^H} NMR (101 MHz, CDCl_3_): δ 160.1, 145.0, 142.2, 140.7, 138.6, 132.4, 130.1, 129.3, 129.0, 128.8, 127.5, 118.1, 117.7, 117.6, 112.7, 111.9, 110.4, 104.4, 55.2, 48.0; MS (*m*/*z*): 358; Anal. Calcd for C_22_H_18_N_2_O_3_: C, 73.73; H, 5.06; N, 7.82; Found: C, 73.61; H, 5.02; N, 7.76.

#### 3.3.3. 5-Nitro-2-phenyl-1-(3-(trifluoromethyl)phenyl)-1*H*-indole (**14**)

Method C: Deoxybenzoin **5** (1 equiv) with 3-(trifluoromethyl)benzylamine (5 equiv) gave indole **14** (103 mg, 67%) as a yellow solid, mp 128–129 °C; IR: 1516, 1328, 1111, 1064 cm^−1^; ^1^H NMR (400 MHz, CDCl_3_): δ 8.64 (d, *J* = 2.2 Hz, 1H), 8.09 (dd, *J* = 9.0, 2.2 Hz, 1H), 7.53 (d, *J* = 7.8 Hz, 1H), 7.47–7.38 (complex, 6H), 7.25 (s, 1H), 7.20 (d, *J* = 9.0 Hz, 1H), 7.07 (d, *J* = 7.8 Hz, 1H), 6.82 (d, *J* = 0.8 Hz, 1H), 5.45 (s, 2H); ^13^C {^1^H} NMR (101 MHz, CDCl_3_): δ 144.9, 142.4, 140.4, 137.9, 131.6, 131.23, 131.15, 129.6, 129.23, 129.15, 128.9, 127.7, 125.1, 123.8 (q, *J* = 272.5 Hz), 124.7 (q, *J* = 3.8 Hz), 122.9 (q, *J* = 3.8 Hz), 117.84, 117.80, 110.1, 47.6; ^19^F {^1^H} NMR (376 MHz, CDCl_3_ referenced to C_6_H_5_F): δ -62.8; MS (*m*/*z*): 396; Anal. Calcd for C_22_H_15_F_3_N_2_O_2_: C, 66.67; H, 3.81; N, 7.07; Found: C, 66.55; H, 3.88; N, 7.08.

#### 3.3.4. 1-(2-Fluorophenethyl)-5-nitro-2-phenyl-1*H*-indole (**15**)

Method C: Deoxybenzoin **5** (1 equiv) with 2-fluorophenethylamine (5 equiv) gave indole **15** (95 mg, 69%) as a yellow solid, mp 121–123 °C; IR: 1518, 1344 cm^−1^; ^1^H NMR (400 MHz, CDCl_3_): δ 8.58 (d, *J* = 2.2 Hz, 1H), 8.12 (dd, *J* = 9.0, 2.2 Hz, 1H), 7.49–7.44 (complex, 3H), 7.38 (d, *J* = 9.0 Hz, 1H), 7.38–7.34 (complex, 2H), 7.15 (m, 1H), 6.90 (m, 2H), 6.72 (td, *J* = 7.5, 1.7 Hz, 1H), 6.65 (d, *J* = 0.8 Hz, 1H), 4.44 (t, *J* = 7.3 Hz, 2H), 2.96 (t, *J* = 7.3 Hz, 2H); ^13^C {^1^H} NMR (101 MHz, CDCl_3_): δ 159.7 (d, *J* = 246.2 Hz), 144.7, 144.5, 143.7, 142.2, 140.4, 129.4 (d, *J* = 8.1 Hz), 127.5, 127.4 (d, *J* = 3.8 Hz), 124.6 (d, *J* = 3.6 Hz), 124.4, 124.1, 124.0, 122.4, 118.2, 117.8, 117.5 (d, *J* = 5.4 Hz), 115.5 (d, *J* = 20.9 Hz), 110.1, 104.2, 64.2 (d, *J* = 11.6 Hz), 42.0 (d, *J* = 5.6 Hz); ^19^F {^1^H} NMR (376 MHz, CDCl_3_ referenced to C_6_H_5_F): δ -118.9; MS (*m*/*z*): 360; Anal. Calcd for C_22_H_17_FN_2_O_2_: C, 73.32; H, 4.75; N, 7.77; Found: C, 73.14; H, 4.76; N, 7.75.

#### 3.3.5. 1-(3-Isopropoxypropyl)-5-nitro-2-phenyl-1*H*-indole (**16**)

Method C: Deoxybenzoin **5** (1 equiv) with 3-isopropoxypropylamine (5 equiv) gave indole **16** (101 mg, 77%) as a yellow solid, mp 44–45 °C; IR: 1512, 1328 cm^−1^; ^1^H NMR (400 MHz, CDCl_3_): δ 8.58 (d, *J* = 2.3 Hz, 1H), 8.13 (dd, *J* = 9.1, 2.3 Hz, 1H), 7.52–7.46 (complex, 6H), 6.69 (d, *J* = 0.8 Hz, 1H), 4.35 (t, *J* = 7.1 Hz, 2H), 3.75 (septet, *J* = 6.1 Hz, 1H), 3.20 (t, *J* = 5.7 Hz, 2H), 1.87 (pentet, *J* = 6.0 Hz, 2H), 1.06 (d, *J* = 6.1 Hz, 6H); ^13^C {^1^H} NMR (101 MHz, CDCl_3_): δ 144.4, 141.8, 140.5, 131.9, 129.3, 128.79, 128.77, 127.2, 117.6, 117.1, 110.1, 104.5, 71.6, 64.2, 41.4, 30.7, 22.0; MS (*m*/*z*): 338; Anal. Calcd for C_20_H_22_N_2_O_3_: C, 70.99; H, 6.55; N, 8.28; Found: C, 71.13; H, 6.51; N, 8.12.

#### 3.3.6. 1-Cyclohexyl-5-nitro-2-phenyl-1*H*-indole (**17**)

Method D: Deoxybenzoin 5 (1 equiv) with cyclohexylamine (10 equiv) at a ring closing temperature of 90–95 °C gave indole 17 (72 mg, 58%) as a yellow solid, mp 120–121 °C; IR: 1516, 1328 cm^−1^; ^1^H NMR (400 MHz, CDCl_3_): δ 8.56 (d, *J* = 2.3 Hz, 1H), 8.07 (dd, *J* = 9.2, 2.3 Hz, 1H), 7.66 (d, *J* = 9.2 Hz, 1H), 7.53–7.46 (complex, 3H), 7.45–7.41 (complex, 2H), 6.62 (s, 1H), 4.23 (tt, *J* = 12.5, 3.9 Hz, 1H), 2.37–2.23 (complex, 2H), 1.98–1.87 (complex, 4H), 1.74 (m, 1H), 1.35–1.24 (complex, 3H); ^13^C {^1^H} NMR (101 MHz, CDCl_3_): δ 144.8, 141.3, 138.7, 132.4, 129.5, 128.8, 128.7, 128.1, 117.7, 116.4, 112.2, 104.5, 56.9, 31.5, 26.1, 25.1; MS (*m*/*z*): 320; Anal. Calcd for C_20_H_20_N_2_O_2_: C, 74.98; H, 6.29; N, 8.74; Found: C, 74.75; H, 6.24; N, 8.71.

#### 3.3.7. 1-(4-Methoxyphenyl)-5-nitro-2-phenyl-1*H*-indole (**18**)

Method C: Deoxybenzoin **5** (1 equiv) with 4-methoxyaniline (5 equiv) gave indole **18** (111 mg, 84%) as a yellow solid, mp 169–171 °C; IR: 2837, 1516, 1333 cm^−1^; ^1^H NMR (400 MHz, CDCl_3_): δ 8.63 (d, *J* = 2.2 Hz, 1H), 8.06 (dd, *J* = 9.1, 2.2 Hz, 1H), 7.28 (m, 5H), 7.22 (d, *J* = 9.1 Hz, 1H), 7.17 (d, *J* = 8.9 Hz, 2H), 6.93 (d, *J* = 8.9 Hz, 2H), 6.92 (d, *J* = 0.8 Hz, 1H), 3.86 (s, 3H); ^13^C {^1^H} NMR (101 MHz, CDCl_3_): δ 159.3, 144.2, 142.3, 142.0, 131.3, 130.0, 129.01, 128.97, 128.4, 128.2, 127.3, 117.7, 117.6, 114.8, 110.6, 104.6, 55.5; MS (*m*/*z*): 344; Anal. Calcd for C_21_H_16_N_2_O_3_: C, 73.24; H, 4.68; N: 8.13; Found: C, 73.32; H, 4.74; N, 8.09.

#### 3.3.8. 5-Nitro-1,2-diphenyl-1*H*-indole (**19**)

Method D: Deoxybenzoin **5** (1 equiv) with aniline (10 equiv) at a ring closure temperature of 120–125 °C gave indole **19** (51 mg, 42%) as a yellow solid, mp 187–188 °C (lit [28] mp 182–185 °C); using aniline (5 equiv) and a ring closure temperature of 90–95 °C produced 28 mg (23%) of **19**; IR: 1516, 1328 cm^−1^; ^1^H NMR (400 MHz, CDCl_3_): δ 8.64 (d, *J* = 2.2 Hz, 1H), 8.08 (dd, *J* = 9.1, 2.2 Hz, 1H), 7.49–7.40 (complex, 4H), 7.29–7.24 (complex 7H), 6.94 (d, *J* = 0.8 Hz, 1H); ^13^C {^1^H} NMR (101 MHz, CDCl_3_): δ 144.1, 142.5, 141.6, 137.3, 131.2, 129.7, 129.0, 128.4, 128.3, 128.2, 127.9, 127.5, 117.8, 117.6, 110.6, 105.0; MS (*m*/*z*): 314; Anal. Calcd for C_20_H_14_N_2_O_2_: C, 76.42; H, 4.49; N: 8.91; Found: C, 76.13; H, 4.44; N, 8.87.

#### 3.3.9. 1-(4-Fluorophenyl)-5-nitro-2-phenyl-1*H*-indole (**20**)

Method D: Deoxybenzoin **5** (1 equiv) with 4-fluoroaniline (10 equiv) at a ring closure temperature of 120–125 °C gave indole **20** (105 mg, 82%) as a yellow solid, mp 149–150 °C; using 4-fluoroaniline (5 equiv) and a ring closure temperature of 90–95 °C produced 12 mg (9%) of **20**; IR: 1516, 1325 cm^−1^; ^1^H NMR (400 MHz, CDCl_3_): δ 8.64 (d, *J* = 2.2 Hz, 1H), 8.09 (dd, *J* = 9.1, 2.2 Hz, 1H), 7.32–7.28 (complex, 3H), 7.28–7.22 (complex, 5H), 7.16 (t, *J* = 8.3 Hz, 2H), 6.94 (d, *J* = 0.9 Hz, 1H); ^13^C {^1^H} NMR (101 MHz, CDCl_3_): δ 162.0 (d, *J* = 249.2 Hz), 144.1, 142.6, 141.7, 133.3 (d, *J* = 3.1 Hz), 131.0, 129.6 (d, *J* = 8.7 Hz), 129.0, 128.5, 128.4, 127.5, 118.0, 117.6, 116.7 (d, *J* = 22.8 Hz), 110.4, 105.1; ^19^F {^1^H} NMR (376 MHz, CDCl_3_ referenced to C_6_H_5_F): δ -112.3; MS (*m*/*z*): 332; Anal. Calcd for C_20_H_13_FN_2_O_2_: C, 72.28; H, 3.94; N, 8.43; Found: C, 71.99; H, 3.91; N, 8.38.

In one case, a mixture of deoxybenzoin **5** (1 equiv) and 4-fluoroaniline (5 equiv) was slowly heated from 80 to 125 °C to give 5-nitro-2-phenylbenzofuran (**20a**, 32 mg, 25%) as the major product; light yellow solid, mp 156–158 °C (lit [29] mp 158–159 °C); IR: 1524, 1351 cm^−1^; ^1^H NMR (400 MHz, CDCl_3_): δ 8.51 (d, *J* = 2.4 Hz, 1H), 8.22 (dd, *J* = 9.0, 2.4 Hz, 1H), 7.88 (m, 2H), 7.59 (d, *J* = 9.0 Hz, 1H), 7.49 (m, 2H), 7.43 (m, 1H), 7.13 (d, *J* = 0.9 Hz, 1H); ^13^C {^1^H} NMR (101 MHz, CDCl_3_): δ 159.3, 157.6, 144.4, 129.7, 129.6, 129.2, 129.0, 125.3, 120.1, 117.3, 111.4, 101.6; MS (*m*/*z*): 239.

#### 3.3.10. 5-Nitro-2-phenyl-1-(4-(trifluoromethyl)phenyl)-1*H*-indole (**21**)

Methods C or D: No 1*H*-indole product was isolated from reaction of 4-(trifluoromethyl)aniline with deoxybenzoin **5**.

#### 3.3.11. 1-Benzyl-2-(4-methylphenyl)-5-nitro-1*H*-indole (**22**)

Method C: Deoxybenzoin **6** (1 equiv) with benzylamine (5 equiv) gave indole **22** (98 mg, 78%) as a yellow solid, mp 127–128 °C; IR: 1523, 1321 cm^−1^; ^1^H NMR (400 MHz, CDCl_3_): δ 8.60 (d, *J* = 2.2 Hz, 1H), 8.03 (dd, *J* = 9.0, 2.2 Hz, 1H), 7.34–7.27 (complex, 5H), 7.23 (d, *J* = 8.1 Hz, 2H), 7.18 (d, *J* = 9.0 Hz, 1H), 6.97 (d, *J* = 7.8 Hz, 2H), 6.76 (s, 1H), 5.39 (s, 2H), 2.40 (s, 3H); ^13^C {^1^H} NMR (101 MHz, CDCl_3_): δ 145.2, 142.1, 140.6, 139.0, 137.0, 129.5, 129.1, 129.0, 128.4, 127.64, 127.55, 125.8, 117.5, 117.4, 110.4, 104.1, 48.0, 21.3; MS (*m*/*z*): 342; Anal. Calcd for C_22_H_18_N_2_O_2_: C, 77.17; H, 5.30; N, 8.18; Found: C, 77.02; H, 5.29; N, 8.14.

#### 3.3.12. 1-(3-Methoxybenzyl)-2-(4-methylphenyl)-5-nitro-1*H*-indole (**23**)

Method C: Deoxybenzoin **6** (1 equiv) with 3-methoxybenzylamine (5 equiv) gave indole **23** (105 mg, 77%) as a yellow solid, mp 93–94 °C; IR: 2844, 1602, 1523, 1321 cm^−1^; ^1^H NMR (400 MHz, CDCl_3_): δ 8.60 (d, *J* = 2.2 Hz, 1H), 8.04 (dd, *J* = 9.1, 2.2 Hz, 1H), 7.33 (d, *J* = 8.3 Hz, 2H), 7.24–7.18 (complex, 4H), 6.80 (dd, *J* = 8.1, 2.5 Hz, 1H), 6.76 (d, *J* = 0.7 Hz, 1H), 6.57 (ddd, *J* = 7.8, 1.8, 0.9 Hz, 1H), 6.51 (t, *J* = 1.8 Hz, 1H), 5.36 (s, 2H), 3.71 (s, 3H), 2.40 (s, 3H); ^13^C {^1^H} NMR (101 MHz, CDCl_3_): δ 160.1, 145.2, 142.1, 140.6, 139.0, 138.7, 130.1, 129.5, 129.2, 128.4, 127.6, 118.1, 117.5, 117.4, 112.6, 111.9, 110.4, 104.0, 55.2, 47.9, 22.3; MS (*m*/*z*): 372; Anal. Calcd for C_23_H_20_N_2_O_3_: C, 74.18; H, 5.41; N, 7.52; Found: C, 74.38; H, 5.45; N, 7.50.

#### 3.3.13. 2-(4-Methylphenyl)-5-nitro-1-phenethyl-1*H*-indole (**24**)

Method C: Deoxybenzoin **6** (1 equiv) with phenethylamine (5 equiv) gave indole **24** (81 mg, 62%) as a yellow solid, mp 120–122 °C; IR: 1523, 1321 cm^−1^; ^1^H NMR (400 MHz, CDCl_3_): δ 8.57 (d, *J* = 2.2 Hz, 1H), 8.10 (dd, *J* = 9.1, 2.2 Hz, 1H), 7.32 (d, *J* = 9.1 Hz, 1H), 7.27 (d, *J* = 8.2 Hz, 2H), 7.22 (d, *J* = 8.2 Hz, 2H), 7.19–7.15 (complex, 3H), 6.87–6.85 (complex, 2H), 6.62 (d, *J* = 0.8 Hz, 1H), 4.40 (t, *J* = 7.5 Hz, 2H), 2.92 (t, *J* = 7.5 Hz, 2H), 2.44 (s, 3H); ^13^C {^1^H} NMR (101 MHz, CDCl_3_): δ 144.7, 141.8, 140.0, 138.8, 137.5, 129.4, 129.3, 128.7, 128.63, 128.58, 127.4, 126.8, 117.6, 117.1, 109.7, 104.2, 45.9, 36.2, 21.4; MS (*m*/*z*): 356; Anal. Calcd for C_23_H_20_N_2_O_2_: C, 77.51; H, 5.66; N, 7.86; Found: C, 77.40; H, 5.67; N, 7.89.

#### 3.3.14. 1-Benzyl-2-(4-methoxyphenyl)-5-nitro-1*H*-indole (**25**)

Method C: Deoxybenzoin **7** (1 equiv) with benzylamine (5 equiv) gave indole **25** (106 mg, 86%) as a yellow solid, mp 143–144 °C; IR: 2837, 1516, 1328 cm^−1^; ^1^H NMR (400 MHz, CDCl_3_: δ 8.60 (d, *J* = 2.2 Hz, 1H), 8.04 (dd, *J* = 9.1, 2.2 Hz, 1H), 7.35 (d, *J* = 8.7 Hz, 2H), 7.31–7.24 (complex, 3H), 7.18 (d, *J* = 9.0 Hz, 1H), 6.98 (d, *J* = 9.0 Hz, 2H), 6.95 (d, *J* = 8.7 Hz, 2H), 6.74 (s, 1H), 5.39 (s, 2H), 3.84 (s, 3H); ^13^C {^1^H} NMR (101 MHz, CDCl_3_): δ 160.2, 145.0, 142.1, 140.5, 137.0, 130.6, 129.0, 127.7, 127.6, 125.8, 123.7, 117.4, 117.3, 114.3, 110.3, 103.8, 55.4, 48.0; MS (*m*/*z*): 358; Anal. Calcd for C_22_H_18_N_2_O_3_: C, 73.73; H, 5.06; N, 7.82; Found: C, 73.66; H, 5.05; N, 7.79.

#### 3.3.15. 1-(3-Methoxybenzyl)-2-(4-methoxyphenyl)-5-nitro-1*H*-indole (**26**)

Method C: Deoxybenzoin **7** (1 equiv) with 3-methoxybenzylamine (5 equiv) gave indole **26** (107 mg, 80%) was isolated as a yellow solid, mp 118–119 °C; IR: 2842, 1516, 1325 cm^−1^; ^1^H NMR (400 MHz, CDCl_3_): δ 8.58 (d, *J* = 2.2 Hz, 1H), 8.03 (dd, *J* = 9.0, 2.2 Hz, 1H), 7.36 (d, *J* = 8.8 Hz, 2H), 7.22 (d, *J* = 8.0 Hz, 1H), 7.18 (d, *J* = 8.7 Hz, 1H), 6.94 (d, *J* = 8.8 Hz, 2H), 6.79 (ddd, *J* = 8.3, 2.5, 0.9 Hz, 1H), 6.72 (d, *J* = 0.9 Hz, 1H), 6.56 (ddd, *J* = 7.6, 1.7, 0.9 Hz, 1H), 6.51 (t, *J* = 2.2 Hz, 1H), 5.35 (s, 2H), 3.84 (s, 3H), 3.71 (s, 3H); ^13^C {^1^H} NMR (101 MHz, CDCl_3_): δ 160.2, 160.1, 144.9, 142.1, 140.6, 138.7, 130.6, 130.1, 127.5, 123.6, 118.0, 117.4, 117.3, 114.3, 112.6, 111.8, 110.3, 103.7, 55.4, 55.2, 47.9; MS (*m*/*z*): 388; Anal. Calcd for C_23_H_20_N_2_O_4_: C, 71.12; H, 5.19; N, 7.21; Found: C, 70.98; H, 5.16; N, 7.15.

#### 3.3.16. 1-(2-Chlorobenzyl)-2-(4-methoxyphenyl)-5-nitro-1*H*-indole (**27**)

Method C: Deoxybenzoin **7** (1 equiv) with 2-chlorobenzylamine (5 equiv) gave indole **27** (104 mg, 77%) was isolated as a yellow solid, mp 147–149 °C; IR: 2841, 1516, 1328 cm^−1^; ^1^H NMR (400 MHz, CDCl_3_): δ 8.62 (d, *J* = 2.2 Hz, 1H), 8.05 (dd, *J* = 9.0, 2.2 Hz, 1H), 7.44 (dd, *J* = 8.0, 1.3 Hz, 1H), 7.31 (d, *J* = 8.8 Hz, 2H), 7.23 (td, *J* = 7.9, 1.6 Hz, 1H), 7.14–7.08 (complex, 2H), 6.96 (d, *J* = 8.8 Hz, 2H), 6.78 (d, *J* = 0.9 Hz, 1H), 6.52 (dd, *J* = 7.5, 1.6 Hz, 1H), 5.43 (s, 2H), 3.83 (s, 3H); ^13^C {^1^H} NMR (101 MHz, CDCl_3_): δ 160.2, 145.0, 142.3, 140.4, 134.4, 131.9, 130.3, 129.8, 128.9, 127.6, 127.4, 126.8, 123.3, 117.50, 117.48, 114.4, 110.1, 103.9, 55.4, 46.0; MS (*m*/*z*): 392, 394 (*ca* 3:1); Anal. Calcd for C_22_H_17_ClN_2_O_3_: C, 67.26; H, 4.36; N, 7.13; Found: C, 67.25; H, 4.39; N, 7.18.

#### 3.3.17. 2-(4-Methoxyphenyl)-5-nitro-1-(3-(trifluoromethyl)benzyl)-1*H*-indole (**28**)

Method C: Deoxybenzoin **7** (1 equiv) with 3-(trifluoromethyl)benzylamine (5 equiv) gave indole **28** (119 mg, 81%) as a yellow solid, mp 115–117 °C; IR: 2844, 1516, 1328, 1105, 1071 cm^−1^; ^1^H NMR (400 MHz, CDCl_3_): δ 8.62 (d, *J* = 2.2 Hz, 1H), 8.08 (dd, *J* = 9.0, 2.2 Hz, 1H), 7.53 (d, *J* = 7.8 Hz, 1H), 7.40 (t, *J* = 7.8 Hz, 1H), 7.30 (d, *J* = 8.7 Hz, 2H), 7.26 (obscured, 1H), 7.18 (d, *J* = 9.0 Hz, 1H), 7.08 (d, *J* = 7.8 Hz, 1H), 6.95 (d, *J* = 8.7 Hz, 2H), 6.76 (d, *J* = 0.8 Hz, 1H), 5.43 (s, 2H), 3.85 (s, 3H); ^13^C {^1^H} NMR (101 MHz, CDCl_3_): δ 160.3, 144.8, 142.3, 140.3, 138.1, 131.5, 131.4 (q, *J* = 32.5 Hz), 130.6, 129.6, 129.2, 127.7, 124.7 (q, *J* = 3.7 Hz), 123.8 (q, *J* = 272.5 Hz), 122.9 (q, *J* = 3.8 Hz), 117.62, 117.58, 114.4, 109.9, 104.3, 55.4, 47.6; ^19^F {^1^H} NMR (376 MHz, CDCl_3_ referenced to C_6_H_5_F): δ -62.8; MS (*m*/*z*): 426; Anal. Calcd for C_23_H_17_F_3_N_2_O_3_: C, 64.79; H, 4.02; N, 6.57; Found: C, 64.89; H, 3.91; N, 6.59.

#### 3.3.18. 2-(4-Methoxyphenyl)-5-nitro-1-phenethyl-1*H*-indole (**29**)

Method C: Deoxybenzoin **7** (1 equiv) with phenethylamine (5 equiv) gave indole **29** (97 mg, 75%) as a yellow solid, mp 141–143 °C; IR: 2838, 1516, 1334 cm^−1^; ^1^H NMR (400 MHz, CDCl_3_): δ 8.56 (d, *J* = 2.2 Hz, 1H), 8.10 (dd, *J* = 9.1, 2.2 Hz, 1H), 7.32 (d, *J* = 9.1 Hz, 1H), 7.24 (d, *J* = 8.8 Hz, 2H), 7.19–7.15 (complex, 3H), 6.98 (d, *J* = 8.8 Hz, 2H), 6.87–6.82 (complex, 2H), 6.59 (d, *J* = 0.7 Hz, 1H), 4.38 (t, *J* = 7.5 Hz, 2H), 3.89 (s, 3H), 2.92 (t, *J* = 7.5 Hz, 2H); ^13^C {^1^H} NMR (101 MHz, CDCl_3_): δ 160.0, 144.5, 141.7, 139.9, 137.5, 130.7, 128.63, 128.59, 127.4, 126.8, 123.9, 117.5, 117.0, 114.1, 109.6, 104.0, 55.4, 45.8, 36.2; MS (*m*/*z*): 372; Anal. Calcd for C_23_H_20_N_2_O_3_: C, 74.18; H, 5.41; N, 7.52; Found: C, 74.07; H, 5.38; N, 7.51.

#### 3.3.19. 1-Hexyl-2-(4-methoxyphenyl)-5-nitro-1*H*-indole (**30**)

Method D: Deoxybenzoin **7** (1 equiv) with hexylamine (10 equiv) at a ring closing temperature of 90–95 °C gave indole **30** (44 mg, 36%) was isolated as a yellow solid, mp 69–70 °C; IR: 2835, 1516, 1330 cm^−1^; ^1^H NMR (400 MHz, CDCl_3_): δ 8.56 (d, *J* = 2.2 Hz, 1H), 8.11 (dd, *J* = 9.0, 2.2 Hz, 1H), 7.40 (d, *J* = 8.7 Hz, 2H), 7.36 (d, *J* = 9.0 Hz, 1H), 7.03 (d, *J* = 8.7 Hz, 2H), 6.61 (d, *J* = 0.8 Hz, 1H), 4.15 (t, *J* = 7.6 Hz, 2H), 3.89 (s, 3H), 1.67 (pentet, *J* = 7.1 Hz, 2H), 1.25–1.12 (complex, 6H), 0.81 (t, *J* = 6.6 Hz, 3H); ^13^C {^1^H} NMR (101 MHz, CDCl_3_): δ 160.0, 144.5, 141.6, 140.0, 130.7, 127.4, 124.2, 117.5, 116.9, 114.2, 109.7, 103.8, 55.4, 44.3, 31.2, 29.9, 26.3, 22.4, 13.9; MS (*m*/*z*): 352; Anal. Calcd for C_21_H_24_N_2_O_3_: C, 71.57; H, 6.86; N, 7.95; Found: C, 71.64; H, 6.91; N, 7.93.

#### 3.3.20. 1-Benzyl-2-(4-fluorophenyl)-5-nitro-1*H*-indole (**31**)

Method C: Deoxybenzoin **8** (1 equiv) with benzylamine (5 equiv) gave indole **31** (102 mg, 85%) as a yellow solid, mp 129–130 °C; IR: 1510, 1335 cm^−1^; ^1^H NMR (400 MHz, CDCl_3_): δ 8.62 (d, *J* = 2.2 Hz, 1H), 8.07 (dd, *J* = 9.0, 2.2 Hz, 1H), 7.38 (dd, *J* = 8.8, 5.2 Hz, 2H), 7.35–7.25 (complex, 3H), 7.22 (d, *J* = 9.0 Hz, 1H), 7.11 (t, *J* = 8.7 Hz, 2H), 6.95 (m, 2H), 6.78 (d, *J* = 0.7 Hz, 1H), 5.37 (s, 2H); ^13^C {^1^H} NMR (101 MHz, CDCl_3_): δ 163.1 (d, *J* = 249.5 Hz,), 143.9, 142.2, 140.6, 136.7, 131.1 (d, *J* = 8.2 Hz), 129.1, 127.8, 127.5 (d, *J* = 3.5 Hz), 127.4, 125.7, 117.7, 116.9 (d, *J* = 21.8 Hz), 110.4, 104.5, 48.0 (1 aromatic carbon unresolved); ^19^F {^1^H} NMR (376 MHz, CDCl_3_ referenced to C_6_H_5_F): δ -111.9; MS (*m*/*z*): 346; Anal. Calcd for C_21_H_15_FN_2_O_2_: C, 72.82; H, 4.37; N, 8.09; Found: C, 72.73; H, 4.33; N, 8.06.

#### 3.3.21. 2-(4-Fluorophenyl)-5-nitro-1-(3-(trifluoromethyl)benzyl)-1*H*-indole (**32**)

Method C: Deoxybenzoin **8** (1 equiv) with 3-(trifluoromethyl)benzylamine (5 equiv) gave indole **32** (81 mg, 55%) as a yellow solid, mp 129–130 °C; IR: 1516, 1327 cm^−1^; ^1^H NMR (400 MHz, CDCl_3_): δ 8.64 (d, *J* = 2.2 Hz, 1H), 8.10 (dd, *J* = 9.0, 2.2 Hz, 1H), 7.54 (d, *J* = 7.8 Hz, 1H), 7.40 (t, *J* = 7.8 Hz, 1H), 7.35 (dd, *J* = 8.8, 5.2 Hz, 2H), 7.24 (br s, 1H), 7.21 (d, *J* = 9.0 Hz, 1H), 7.13 (t, *J* = 8.7 Hz, 2H), 7.05 (br d, *J* = 7.8 Hz, 1H), 6.80 (d, *J* = 0.8 Hz, 1H), 5.42 (s, 2H); ^13^C {^1^H} NMR (101 MHz, CDCl_3_): δ 163.2 (d, *J* = 250.1 Hz), 143.7, 142.4, 140.4, 137.8, 131.5 (q, *J* = 32.6 Hz), 131.1 (d, *J* = 8.2 Hz), 129.7, 129.1, 127.5, 127.2 (d, *J* = 3.6 Hz), 124.8 (q, *J* = 3.8 Hz), 123.7 (q, *J* = 272.5 Hz), 122.8 (q, *J* = 3.8 Hz), 117.94, 117.86, 116.1 (d, *J* = 21.7 Hz), 110.1, 105.0, 47.6; ^19^F {^1^H} NMR (376 MHz, CDCl_3_ referenced to C_6_H_5_F): δ -62.9, -111.5; MS (*m*/*z*): 414: Anal. Calcd for C_22_H_14_F_4_N_2_O_2_: C, 63.77; H, 3.41; N, 6.76; Found: C, 63.49; H, 3.40; N, 6.70.

#### 3.3.22. 2-(4-Fluorophenyl)-5-nitro-1-phenethyl-1*H*-indole (**33**)

Method C: Deoxybenzoin **8** (1 equiv) with phenethylamine (5 equiv) gave indole **33** (103 mg, 79%) as a yellow solid, mp 129–130 °C; IR: 1516, 1338 cm^−1^; ^1^H NMR (400 MHz, CDCl_3_): δ 8.58 (d, *J* = 2.2 Hz, 1H), 8.13 (dd, *J* = 9.0, 2.2 Hz, 1H), 7.37 (d, *J* = 9.0 Hz, 1H), 7.23–7.09 (complex, 7H), 6.79 (m, 2H), 6.60 (s, 1H), 4.39 (t, *J* = 7.2 Hz, 2H), 2.92 (t, *J* = 7.2 Hz, 2H); ^13^C {^1^H} NMR (101 MHz, CDCl_3_): δ 163.0 (d, *J* = 249.3 Hz), 143.5, 141.9, 139.8, 137.4, 131.2 (d, *J* = 8.4 Hz), 128.7, 128.6, 127.7 (d, *J* = 3.4 Hz), 127.3, 126.9, 117.8, 117.4, 115.7 (d, *J* = 21.7 Hz), 109.8, 104.5, 45.8, 36.1; ^19^F {^1^H} NMR (376 MHz, CDCl_3_ referenced to C_6_H_5_F): δ -112.3; MS (*m*/*z*): 360; Anal. Calcd for C_22_H_17_FN_2_O_2_: C, 73.32; H, 4.75; N, 7.77; Found: C, 73.19; H, 4.77; N, 7.73.

#### 3.3.23. 1-Isobutyl-2-(4-fluorophenyl)-5-nitro-1*H*-indole (**34**)

Method D: Deoxybenzoin **8** (1 equiv) with isobutylamine (10 equiv) at a ring closing temperature of 90–95 °C gave indole **34** (36 mg, 32%) as a yellow solid, mp 93–94 °C; IR: 1516, 1320 cm^−1^; ^1^H NMR (400 MHz, CDCl_3_): δ 8.57 (d, *J* = 2.2 Hz, 1H), 8.12 (dd, *J* = 9.1, 2,2 Hz, 1H), 7.45 (dd, *J* = 8.7, 5.3 Hz, 2H), 7.38 (d, *J* = 9.1 Hz, 1H), 7.19 (t, *J* = 8.7 Hz, 2H), 6.65 (d, *J* = 0.8 Hz, 1H), 4.02 (d, *J* = 7.6 Hz, 2H), 1.96 (nonet, *J* = 6.9 Hz, 1H), 0.68 (d, *J* = 6.7 Hz, 6H); ^13^C {^1^H} NMR (101 MHz, CDCl_3_): δ 161.9 (d, *J* = 249.1 Hz), 142.7, 140.7, 139.4, 130.5 (d, *J* = 8.3 Hz), 127.2 (d, *J* = 3.4 Hz), 126.1, 116.7, 116.2, 114.8 (d, *J* = 21.7 Hz), 109.2, 103.7, 50.6, 28.1, 19.0; ^19^F {^1^H} NMR (376 MHz, CDCl_3_ referenced to C_6_H_5_F): δ -112.3; MS (*m*/*z*): 312; Anal. Calcd for C_18_H_17_FN_2_O_2_: C, 69.22; H, 5.49; N, 8.97; Found: C, 69.10; H, 5.49; N, 8.94.

#### 3.3.24. 1-Benzyl-2-(4-fluoro-3-methylphenyl)-5-nitro-1*H*-indole (**35**)

Method C: Deoxybenzoin **9** (1 equiv) with benzylamine (5 equiv) gave indole **35** (103 mg, 83%) as a yellow solid, mp 124–125 °C; IR: 1516, 1325 cm^−1^; ^1^H NMR (400 MHz, CDCl_3_): δ 8.61 (d, *J* = 2.2 Hz, 1H), 8.06 (dd, *J* = 9.0, 2.2 Hz, 1H), 7.32–7.25 (complex, 3H), 7.24–7.17 (complex, 3H), 7.04 (d, *J* = 9.0 Hz, 1H), 6.95 (m, 2H), 6.75 (d, *J* = 0.8 Hz, 1H), 5.37 (s, 2H), 2.26 (d, *J* = 1.9 Hz, 3H); ^13^C {^1^H} NMR (101 MHz, CDCl_3_): δ 161.7 (d, *J* = 248.1 Hz), 144.2, 142.2, 140.5, 136.9, 132.7 (d, *J* = 5.5 Hz), 129.0, 128.3 (d, *J* = 8.4 Hz), 127.8, 127.4, 127.1 (d, *J* = 3.9 Hz), 125.8, 125.5 (d, *J* = 17.7 Hz), 117.62, 117.57, 115.4 (d, *J* = 22.7 Hz), 110.3, 104.3, 48.0, 14.7 (d, *J* = 3.5 Hz); ^19^F {^1^H} NMR (376 MHz, CDCl_3_ referenced to C_6_H_5_F): δ -116.3; MS (*m*/*z*): 360; Anal. Calcd for C_22_H_17_FN_2_O_2_: C, 73.32; H, 4.75; N, 7.77; Found: C, 73.16; H, 4.76; N, 7.79.

#### 3.3.25. 2-(4-Fluoro-3-methylphenyl)-5-nitro-1-phenethyl-1*H*-indole (**36**)

Method C: Deoxybenzoin **9** (1 equiv) with phenethylamine (5 equiv) gave indole **36** (102 mg, 80%) as a yellow solid, mp 126–128 °C; IR: 1516, 1329 cm^−1^; ^1^H NMR (400 MHz, CDCl_3_): δ 8.57 (d, *J* = 2.2 Hz, 1H), 8.06 (dd, *J* = 9.0, 2.2 Hz, 1H), 7.34 (d, *J* = 9.0 Hz, 1H), 7.21–7.13 (complex, 3H), 7.08–7.01 (complex, 2H), 6.98 (d, *J* = 7.1 Hz, 1H), 6.78 (m, 2H), 6.57 (s, 1H), 4.39 (t, *J* = 7.2 Hz, 2H), 2.93 (t, *J* = 7.2 Hz, 2H), 2.31 (d, *J* = 2.0 Hz, 3H); ^13^C {^1^H} NMR (101 MHz, CDCl_3_): δ 161.6 (d, *J* = 247.9 Hz), 143.9, 141.9, 139.8, 137.5, 132.7 (d, *J* = 5.4 Hz), 128.6, 128.4 (d, *J* = 8.2 Hz), 127.4, 127.3, 126.8, 125.3 (d, *J* = 17.6 Hz), 117.7, 117.3, 115.2 (d, *J* = 22.7 Hz), 109.8, 104.3, 45.1, 36.1, 14.6 (d, *J* = 3.5 Hz), (1 aromatic carbon unresolved); ^19^F {^1^H} NMR (376 MHz, CDCl_3_ referenced to C_6_H_5_F): δ -116.7; MS (*m*/*z*): 374; Anal. Calcd for C_23_H_19_FN_2_O_2_: C, 73.78; H, 5.12; N, 7.48; Found: C, 73.58; H, 5.07; N, 7.46.

#### 3.3.26. 2-(4–Fluoro-3-methylphenyl)-1-(2-fluorophenethyl)-5-nitro-1*H*-indole (**37**)

Method C: Deoxybenzoin **9** (1 equiv) with 2-fluorophenethylamine (5 equiv) gave indole **37** (101 mg, 75%) as a yellow solid, mp 129–130 °C; IR: 1516, 1328 cm^−1^; ^1^H NMR (400 MHz, CDCl_3_): δ 8.57 (d, *J* = 2.2 Hz, 1H), 8.13 (dd, *J* = 9.0, 2.2 Hz, 1H), 7.39 (d, *J* = 9.0 Hz, 1H), 7.20–7.05 (complex, 4H), 6.91 (m, 2H), 6.71 (td, *J* = 7.8, 1.9 Hz, 1H), 6.58 (d, *J* = 0.8 Hz, 1H), 4.41 (t, *J* = 7.2 Hz, 2H), 2.97 (t, *J* = 7.2 Hz, 2H), 2.33 (d, *J* = 2.0 Hz, 3H); ^13^C {^1^H} NMR (101 MHz, CDCl_3_): δ 161.6 (d, *J* = 247.7 Hz), 161.2 (d, *J* = 245.5 Hz), 143.7, 141.9, 139.9, 132.6 (d, *J* = 5.5 Hz), 130.9 (d, *J* = 4.7 Hz), 128.8 (d, *J* = 8.2 Hz), 128.3 (d, *J* = 8.3 Hz), 127.3, 127.2 (d, *J* = 3.8 Hz), 125.4 (d, *J* = 17.7 Hz), 124.3 (d, *J* = 13.1 Hz), 124.2, 117.7, 117.3, 115.33 (d, *J* = 21.7 Hz), 115.28 (d, *J* = 22.8 Hz), 109.7, 104.3, 44.2, 30.0 (d, *J* = 1.9 Hz), 14.6 (d, *J* = 3.5 Hz); ^19^F {^1^H} NMR (376 MHz, CDCl_3_ referenced to C_6_H_5_F): δ -116.6, -118.9; MS (*m*/*z*): 392; Anal. Calcd for C_23_H_18_F_2_N_2_O_2_: C, 70.40; H, 4.62; N, 7.14; Found: C, 70.29; H, 4.54; N, 7.16.

#### 3.3.27. 1-Benzyl-2-(4-chlorophenyl)-5-nitro-1*H*-indole (**38**)

Method C: Deoxybenzoin **10** (1 equiv) with benzylamine (5 equiv) gave indole **38** (95 mg, 77%) as a yellow solid, mp 129–130 °C; IR: 1516, 1331 cm^−1^; ^1^H NMR (400 MHz, CDCl_3_): δ 8.62 (d, *J* = 2.2 Hz, 1H), 8.07 (dd, *J* = 9.1, 2.2 Hz, 1H), 7.40 (d, *J* = 8.6 Hz, 2H), 7.35 (d, *J* = 8.6 Hz, 2H), 7.33–7.27 (complex, 3H), 7.22 (d, *J* = 9.1 Hz, 1H), 6.95 (m, 2H), 6.80 (d, *J* = 0.8 Hz, 1H), 5.38 (s, 2H); ^13^C {^1^H} NMR (101 MHz, CDCl_3_): δ 143.7, 142.3, 140.7, 136.7, 135.2, 130.5, 129.8, 129.1, 127.8, 127.4, 125.7, 117.80, 117.76, 110.5, 104.7, 48.1 (1 aromatic carbon unresolved); MS (*m*/*z*) 362, 364 (*ca* 3:1); Anal. Calcd for C_21_H_15_ClN_2_O_2_: C, 69.52; H, 4.17; N, 7.72; Found: C, 69.23; H, 4.10; N, 7.71.

#### 3.3.28. 2-(4-Chlorophenyl)-5-nitro-1-(3-(trifluoromethyl)benzyl)-1*H*-indole (**39**)

Method C: Deoxybenzoin **10** (1 equiv) with 3-(trifluoromethyl)benzylamine (5 equiv) gave indole **39** (91 mg, 62%) as a yellow solid, mp 150–151 °C; IR: 1516, 1333, 1111, 1071 cm^−1^; ^1^H NMR (400 MHz, CDCl_3_): δ 8.63 (d, *J* = 2.2 Hz, 1H), 8.10 (dd, *J* = 9.1, 2.2 Hz, 1H), 7.54 (d, *J* = 7.8 Hz, 1H), 7.42 (d, *J* = 8.5 Hz, 2H), 7.40 (obscured, 1H), 7.32 (d, *J* = 8.5 Hz, 2H), 7.30 (br s, 1H), 7.21 (d, *J* = 9.1 Hz, 1H), 7.05 (d, *J* = 7.8 Hz, 1H), 6.82 (d, *J* = 0.8 Hz, 1H), 5.43 (s, 2H); ^13^C {^1^H} NMR (101 MHz, CDCl_3_): δ 143.5, 142.5, 140.5, 137.7, 135.5, 131.5 (q, *J* = 32.5 Hz), 130.4, 129.7, 129.6, 129.2, 129.0, 127.5, 124.8 (q, *J* = 3.6 Hz), 123.7 (q, *J* = 272.5 Hz), 122.7 (q, *J* = 3.7 Hz), 118.1, 117.9, 110.1, 105.2, 47.6; ^19^F {^1^H} NMR (376 MHz, CDCl_3_ referenced to C_6_H_5_F): δ -62.9; MS (*m*/*z*): 430, 432 (*ca* 3:1); Anal. Calcd for C_22_H_14_ClF_3_N_2_O_2_: C, 61.34; H, 3.28; N, 6.50; Found: C, 61.07; H, 3.33; N, 6.45.

#### 3.3.29. 2-(4-Chlorophenyl)-5-nitro-1-phenethyl-1*H*-indole (**40**)

Method C: Deoxybenzoin **10** (1 equiv) with phenethylamine (5 equiv) gave indole **40** (93 mg, 73%) as a yellow solid, mp 153–154 °C; IR: 1516, 1333 cm^−1^; ^1^H NMR (400 MHz, CDCl_3_): δ 8.58 (d, *J* = 2.2 Hz, 1H), 8.13 (d, *J* = 9.1, 2.2 Hz, 1H), 7.40 d, *J* = 8.5 Hz, 2H), 7.36 (d, *J* = 9.1 Hz, 1H), 7.21–7.12 (obscured, 3H), 7.16 (d, *J* = 8.6 Hz, 2H), 6.78 (m, 2H), 6.61 (s, 1H), 4.40 (t, *J* = 7.2 Hz, 2H), 2.93 (t, *J* = 7.2 Hz, 2H); ^13^C {^1^H} NMR (101 MHz, CDCl_3_): δ 143.3, 142.0, 140.0, 137.3, 134.9, 130.6, 130.1, 128.9, 128.7, 128.6, 127.3, 126.9, 117.8, 117.5, 109.9, 104.7, 45.8, 36.1; MS (*m*/*z*): 376, 378 (*ca* 3:1); Anal. Calcd for C_22_H_17_ClN_2_O_2_: C, 70.12; H, 4.55; N, 7.43; Found: C, 69.98; H, 4.49; N, 7.49.

#### 3.3.30. 1-Benzyl-2-(2,3-dihydrobenzo[*b*][1,4]dioxin-6-yl-)-5-nitro-1*H*-indole (**41**)

Method C: Deoxybenzoin **11** (1 equiv) with benzylamine (5 equiv) gave indole **41** (103 mg, 85%) as a yellow solid, mp 124–125 °C; IR: 1518, 1336 cm^−1^; ^1^H NMR (400 MHz, CDCl_3_): δ 8.59 (d, *J* = 2.2 Hz, 1H), 8.03 (dd, *J* = 9.1, 2.2 Hz, 1H), 7.31–7.24 (complex, 3H), 7.16 (d, *J* = 9.1 Hz, 1H), 6.96 (m, 3H), 6.89 (m, 2H), 6.73 (d, *J* = 0.8 Hz, 1H), 5.41 (s, 2H), 4.29 (m, 4H); ^13^C {^1^H} NMR (101 MHz, CDCl_3_): δ 144.7, 144.4, 143.7, 142.1, 140.5, 136.9, 129.0, 127.7, 127.5, 125.8, 124.6, 122.5, 118.3, 117.7, 117.5, 117.4, 110.4, 103.9, 64.5, 64.3, 48.0; MS (*m*/*z*): 386; Anal. Calcd for C_23_H_18_N_2_O_4_: C, 71.49; H, 4.70; N, 7.25; Found: C, 71.43; H, 4.73; N, 7.29.

#### 3.3.31. 2-(2,3-Dihydobenzo[*b*][1,4]dioxin-6-yl)-1-(2-fluorobenzyl)-5-nitro-1*H*-indole (**42**)

Method C: Deoxybenzoin **11** (1 equiv) with 2-fluorobenzylamine (5 equiv) gave indole **42** (109 mg, 86%) as a yellow solid, mp 153–155 °C; IR: 1520, 1334 cm^−1^; ^1^H NMR (400 MHz, CDCl_3_): δ 8.60 (d, *J* = 2.2 Hz, 1H), 8.05 (dd, *J* = 9.0, 2.2 Hz, 1H), 7.25 (m, 1H), 7.18 (d, *J* = 9.0 Hz, 1H), 7.09 (ddd, *J* = 10.1, 8.3, 1.2 Hz, 1H), 6.98 (td, *J* = 7.6, 1.2 Hz, 1H), 6.95 (d, *J* = 2.0 Hz, 1H), 6.91 (d, *J* = 8.3 Hz, 1H), 6.88 (dd, *J* = 8.3, 2.0 Hz, 1H), 6.74 (d, *J* = 0.8 Hz, 1H), 6.56 (td, *J* = 7.6, 1.7 Hz, 1H), 5.45 (s, 2H), 4.30 (m, 4H); ^13^C {^1^H} NMR (101 MHz, CDCl_3_): δ 159.7 (d, *J* = 246.2 Hz), 144.7, 144.5, 143.7, 142.2, 140.4, 129.4 (d, *J* = 8.1 Hz), 127.5, 127.4 (d, *J* = 3.9 Hz), 124.6 (d, *J* = 3.6 Hz), 124.4, 124.0 (d, *J* = 14.2 Hz), 122.4, 118.2, 117.7, 117.52, 117.47, 115.5 (d, *J* = 20.8 Hz), 110.1, 104.2, 64.5, 64.3, 42.0 (d, *J* = 5.5 Hz); ^19^F {^1^H} NMR (376 MHz, CDCl_3_ referenced to C_6_H_5_F): δ -118.9; MS (*m*/*z*): 404; Anal. Calcd for C_23_H_17_FN_2_O_4_: C, 68.31; H, 4.24; N, 6.93; Found: C, 68.19; H, 4.27; N, 6.89.

#### 3.3.32. 2-(2,3-Dihydobenzo[*b*][1,4]dioxin-6-yl)-5-nitro-1-phenethyl-1*H*-indole (**43**)

Method C: Deoxybenzoin **11** (1 equiv) with phenethylamine (5 equiv) gave indole **43** (109 mg, 86%) as a yellow solid, mp 104–105 °C; IR: 1516, 1334 cm^−1^; ^1^H NMR (400 MHz, CDCl_3_): δ 8.55 (d, *J* = 2.2 Hz, 1H), 8.09 (dd, *J* = 9.1, 2.2 Hz, 1H), 7.30 (d, *J* = 9.1 Hz, 1H), 7.16 (m, 3H), 6.94 (d, *J* = 8.3 Hz, 1H), 6.87 (m, 2H), 6.84 (dd, *J* = 2.1 Hz, 1H), 6.80 (dd, *J* = 8.3, 2.1 Hz, 1H), 6.53 (d, *J* = 0.8 Hz, 1H), 4.41 (t, *J* = 7.5 Hz, 2H), 4.33 (m, 4H), 2.92 (t, *J* = 7.5 Hz, 2H); ^13^C {^1^H} NMR (101 MHz, CDCl_3_): δ 144.22, 144.17, 143.6, 141.8, 139.9, 137.6, 128.6, 127.3, 126.9, 124.9, 122.5, 118.4, 117.6, 117.5, 117.1, 109.7, 104.1, 64.5, 64.4, 45.9, 36.2 (1 aromatic carbon unresolved); MS (*m*/*z*): 400; Anal. Calcd for C_24_H_20_N_2_O_4_: C, 71.99; H, 5.03; N, 7.00; Found: C, 71.95; H, 4.98; N, 6.97.

## 4. Conclusions

A new transition metal-free approach to the synthesis of 1,2,5-trisubstituted 1*H*-indoles has been developed via deprotonation-S_N_Ar cyclization of Schiff bases derived from 1-aryl-2-(2-fluoro-5-nitrophenyl)ethan-1-one (deoxybenzoin) precursors. This study was inspired by an earlier synthesis of benzo[*d*]oxazoles from suitably substituted *N*-(2-fluoroaryl)anilides which readily cyclized using an analogous strategy. However, due to the limited availability of deoxybenzoins bearing C2 fluoroaromatics with C5 S_N_Ar activating groups, only the substrate having NO_2_ activation was fully explored. One attempt with a CF_3_-substituted acceptor ring was unsuccessful as the elevated temperature required for the ring closure resulted in significant degradation of the substrate. Seven NO_2_-activated deoxybenzoins were prepared using two variants of the Friedel-Crafts acylation. Once in hand, these precursors were converted to their Schiff bases by condensation with 5 equiv of a primary amine in boiling toluene slowly distilled at 120–125 °C for 18–24 h. Solvent replacement with dry DMF, addition of 2 equiv of anhydrous K_2_CO_3_, and further heating at 90–95 °C for 18–24 h then afforded the 1*H*-indole products in 32–86% yields following aqueous workup and chromatography. The reaction proceeded best with benzyl- and phenethylamines. High-boiling aliphatic primary amines also gave high yields of the desired heterocycles. A 10-fold excess of volatile amines was required but gave lower conversions due to inefficient reaction at lower temperatures and co-distillation with toluene during the condensation step. Anilines afforded excellent yields when rings were substituted with electron-donating groups and low yields (or no yields) when electron-withdrawing substituents were present. A 10-fold excess of aniline and 4-fluoroaniline and a cyclization temperature of 120–125 °C was required for successful 1*H*-indole formation and, due to resonance electron donation, 4-fluoroaniline proved to be an excellent nucleophile in this reaction. The conversion likely starts from the imine form of the Schiff base, but the conjugated enamine undoubtedly also participates in this process. Several predicted side reactions, e.g., competing nucleophilic aromatic substitutions and cyclization from the oxygen of an intermediate enolate did not cause major deviations from the desired reaction path. We are continuing our efforts to utilize the S_N_Ar reaction as a terminating reaction in the synthesis of heterocyclic targets.

## Figures and Tables

**Table 1:** Synthesis of deoxybenzoin 1*H*-indole precursors.

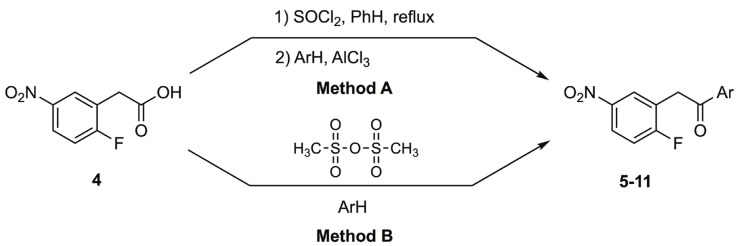		
Ar	Method	Yield (%)
Phenyl (**5**)	A	82
4-Methylphenyl (**6**)	A or B	80 (A) 62 (B)
4-Methoxyphenyl (**7**)	A	78
4-Fluorophenyl (**8**)	A	78
1-(4-Fluoro-3-methylphenyl) (**9**)	B	65
1-(4-Chlorophenyl) (**10**)	A	64
1-(2,3-Dihydrobenzo[*b*][1,4]dioxin-6-yl) (**11**)	B	85

**Table 2:** 1,2,5-Trisubstituted 1*H*-indoles prepared.

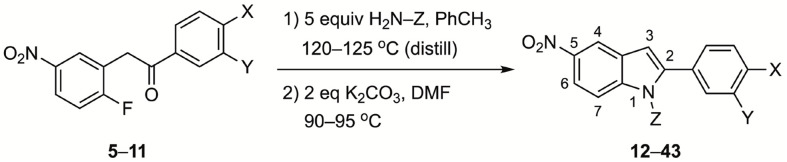						
Entry	Substrate	X	Y	Z	Product	Yield (%)
1	**5**	–H	–H	–CH_2_C_6_H_5_	**12**	80
2				–CH_2_C_6_H_4_-3-OCH_3_	**13**	83
3				–CH_2_C_6_H_4_-3-CF_3_	**14**	67
4				–CH_2_CH_2_C_6_H_4_-2-F	**15**	69
5				–CH_2_CH_2_CH_2_O-*i*-Pr	**16**	77
6				–*c*-C_6_H_11_	**17**	58
7				–C_6_H_4_-4-OCH_3_	**18**	84
8				–C_6_H_5_	**19**	42 ^a^
9				–C_6_H_4_-4-F	**20**	82 ^b^
10				–C_6_H_4_-4-CF_3_	**21**	0
11	**6**	–CH_3_	–H	–CH_2_C_6_H_5_	**22**	78
12				–CH_2_C_6_H_4_-3-OCH_3_	**23**	77
13				–CH_2_CH_2_C_6_H_5_	**24**	62
14	**7**	–OCH_3_	–H	–CH_2_C_6_H_5_	**25**	86
15				–CH_2_C_6_H_4_-3-OCH_3_	**26**	80
16				–CH_2_C_6_H_4_-2-Cl	**27**	77
17				–CH_2_C_6_H_4_-3-CF_3_	**28**	81
18				–CH_2_CH_2_C_6_H_5_	**29**	75
19				–CH_2_(CH_2_)_4_CH_3_	**30**	36
20	**8**	–F	–H	–CH_2_C_6_H_5_	**31**	85
21				–CH_2_C_6_H_4_-3-CF_3_	**32**	55
22				–CH_2_CH_2_C_6_H_5_	**33**	79
23				–CH_2_CH(CH_3_)_2_	**34**	32
24	**9**	–F	–CH_3_	–CH_2_C_6_H_5_	**35**	83
25				–CH_2_CH_2_C_6_H_5_	**36**	80
26				–CH_2_CH_2_C_6_H_4_-2-F	**37**	75
27	**10**	–Cl	–H	–CH_2_C_6_H_5_	**38**	77
28				–CH_2_C_6_H_4_-3-CF_3_	**39**	62
29				–CH_2_CH_2_C_6_H_5_	**40**	73
30	**11**	–OCH_2_CH_2_O–		–CH_2_C_6_H_5_	**41**	85
31				–CH_2_C_6_H_4_-2-F	**42**	86
32				–CH_2_CH_2_C_6_H_5_	**43**	86

^a^ 10 equiv of aniline was used at a reaction temperature of 120–125 °C. When 5 equiv of aniline was used at a reaction temperature of 90–95 C, the yield was 23%. ^b^ 10 equiv of 4-fluoroaniline was used at a reaction temperature of 120–125 °C. When 5 equiv of 4-fluoroaniline was used at a reaction temperature of 90–95 °C, the yield was 9%.

## Data Availability

No new data were created or analyzed in this study. Data sharing is not applicable to this article.

## Supplementary Materials

The following supporting information can be downloaded at: https://www.mdpi.com/article/10.3390/molecules30193894/s1, Copies of ^1^H NMR and ^13^C NMR spectra for all substrates and 1*H*-indole products are provided. ^19^F NMR spectra are provided for compounds that incorporate fluorine.
