# Supplementary material for: 1H-Indoles from Deoxybenzoin Schiff Bases by Deprotonation—SNAr Cyclization
Source: Molecules. 2025 Sep 26;30(19):3894. doi: 10.3390/molecules30193894 (PMC12526362; doi:10.3390/molecules30193894)

1*H*-Indoles from Deoxybenzoin Schiff Bases by Deprotonation—S$_N$Ar Cyclization

Nash E. Nevels and Richard A. Bunce *

Department of Chemistry, Oklahoma State University, Stillwater, OK 74078-3071, USA; nnevels@okstate.edu
* Correspondence: richard.a.bunce@okstate.edu; Tel.: +1-405-744-5952

Supplemental Information

^{1}H NMR spectra were measured at 400 MHz in $CDCl_3$ with Me_4Si as the standard
^{13}C NMR spectra were measured at 101 MHz in $CDCl_3$ with Me_4Si as the standard
^{19}F NMR spectra were measured in at 376 MHz in $CDCl_3$ with fluorobenzene as the standard

Compound **Page**

^{1}H NMR of 2-(2-fluoro-5-nitrophenyl)-1-phenylethan-1-one (**5**) 4
^{13}C NMR of 2-(2-fluoro-5-nitrophenyl)-1-phenylethan-1-one (**5**) 4
^{19}F NMR of 2-(2-fluoro-5-nitrophenyl)-1-phenylethan-1-one (**5**) 5

^{1}H NMR of 2-(2-fluoro-5-nitrophenyl)-1-(4-methylphenyl)ethan-1-one (**6**) 6
^{13}C NMR of 2-(2-fluoro-5-nitrophenyl)-1-(4-methylphenyl)ethan-1-one (**6**) 6
^{19}F NMR of 2-(2-fluoro-5-nitrophenyl)-1-(4-methylphenyl)ethan-1-one (**6**) 7

^{1}H NMR of 2-(2-fluoro-5-nitrophenyl)-1-(4-methoxyphenyl)ethan-1-one (**7**) 8
^{13}C NMR of 2-(2-fluoro-5-nitrophenyl)-1-(4-methoxyphenyl)ethan-1-one (**7**) 8
^{19}F NMR of 2-(2-fluoro-5-nitrophenyl)-1-(4-methoxyphenyl)ethan-1-one (**7**) 9

^{1}H NMR of 2-(2-fluoro-5-nitrophenyl)-1-(4-fluorophenyl)ethan-1-one (**8**) 10
^{13}C NMR of 2-(2-fluoro-5-nitrophenyl)-1-(4-fluorophenyl)ethan-1-one (**8**) 10
^{19}F NMR of 2-(2-fluoro-5-nitrophenyl)-1-(4-fluorophenyl)ethan-1-one (**8**) 11

^{1}H NMR of 1-(4-fluoro-3-methylphenyl)- 2-(2-fluoro-5-nitrophenyl)ethan-1-one (**9**) 12
^{13}C NMR of 1-(4-fluoro-3-methylphenyl)- 2-(2-fluoro-5-nitrophenyl)ethan-1-one (**9**) 12
^{19}F NMR of 1-(4-fluoro-3-methylphenyl)- 2-(2-fluoro-5-nitrophenyl)ethan-1-one (**9**) 13

^{1}H NMR of 1-(4-chlorophenyl)-2-(2-fluoro-5-nitrophenyl)ethan-1-one (**10**) 14
^{13}C NMR of 1-(4-chlorophenyl)-2-(2-fluoro-5-nitrophenyl)ethan-1-one (**10**) 14
^{19}F NMR of 1-(4-chlorophenyl)-2-(2-fluoro-5-nitrophenyl)ethan-1-one (**10**) 15

^{1}H NMR of 1-(2,3-dihydrobenzo[*b*][1,4]dioxin-6-yl)-2-(2-fluoro-5-nitrophenyl)ethan-1-one (**11**) 16
^{13}C NMR of 1-(2,3-dihydrobenzo[*b*][1,4]dioxin-6-yl)-2-(2-fluoro-5-nitrophenyl)ethan-1-one (**11**) 16
^{19}F NMR of 1-(2,3-dihydrobenzo[*b*][1,4]dioxin-6-yl)-2-(2-fluoro-5-nitrophenyl)ethan-1-one (**11**) 17

^{1}H NMR of 1-benzyl-5-nitro-2-phenyl-1*H*-indole (**12**) 18
^{13}C NMR of 1-benzyl-5-nitro-2-phenyl-1*H*-indole (**12**) 18

^{1}H NMR of 1-(3-methoxybenzyl)-5-nitro-2-phenyl-1*H*-indole (**13**) 19
^{13}C NMR of 1-(3-methoxybenzyl)-5-nitro-2-phenyl-1*H*-indole (**13**) 19

^{1}H NMR of 5-nitro-2-phenyl-1-(3-(trifluoromethyl)phenyl)-1*H*-indole (**14**) 20
^{13}C NMR of 5-nitro-2-phenyl-1-(3-(trifluoromethyl)phenyl)-1*H*-indole (**14**) 20
^{19}F NMR of 5-nitro-2-phenyl-1-(3-(trifluoromethyl)phenyl)-1*H*-indole (**14**) 21

^{1}H NMR of 1-(2-fluorophenethyl)-5-nitro-2-phenyl-1*H*-indole (**15**) 22
^{13}C NMR of 1-(2-fluorophenethyl)-5-nitro-2-phenyl-1*H*-indole (**15**) 22
^{19}F NMR of 1-(2-fluorophenethyl)-5-nitro-2-phenyl-1*H*-indole (**15**) 23

^{1}H NMR of 1-(3-isopropoxypropyl)-5-nitro-2-phenyl-1*H*-indole (**16**) 24
^{13}C NMR of 1-(3-isopropoxypropyl)-5-nitro-2-phenyl-1*H*-indole (**16**) 24

^{1}H NMR of 1-cyclohexyl-5-nitro-2-phenyl-1*H*-indole (**17**) 25
^{13}C NMR of 1-cyclohexyl-5-nitro-2-phenyl-1*H*-indole (**17**) 25

^{1}H NMR of 1-(4-methoxyphenyl)-5-nitro-2-phenyl-1*H*-indole (**18**) 26
^{13}C NMR of 1-(4-methoxyphenyl)-5-nitro-2-phenyl-1*H*-indole (**18**)........ 26

^{1}H NMR of 5-nitro-1,2-diphenyl-1*H*-indole (**19**) 27
^{13}C NMR of 5-nitro-1,2-diphenyl-1*H*-indole (**19**) 27

^{1}H NMR of 1-(4-fluorophenyl)-5-nitro-2-phenyl-1*H*-indole (**20**) 28
^{13}C NMR of 1-(4-fluorophenyl)-5-nitro-2-phenyl-1*H*-indole (**20**)........ 28
^{19}F NMR of 1-(4-fluorophenyl)-5-nitro-2-phenyl-1*H*-indole (**20**)........ 29

^{1}H NMR of 5-nitro-2-phenylbenzofuran (**20a**) 30
^{13}C NMR of 5-nitro-2-phenylbenzofuran (**20a**) 30

^{1}H NMR of 1-benzyl-2-(4-methylphenyl)-5-nitro-1*H*-indole (**22**) 31
^{13}C NMR of 1-benzyl-2-(4-methylphenyl)-5-nitro-1*H*-indole (**22**) 31

^{1}H NMR of 1-(3-methoxybenzyl)-2-(4-methylphenyl)-5-nitro-1*H*-indole (**23**) 32
^{13}C NMR of 1-(3-methoxybenzyl)-2-(4-methylphenyl)-5-nitro-1*H*-indole (**23**) 32

^{1}H NMR of 2-(4-methylphenyl)-5-nitro-1-phenethyl-1*H*-indole (**24**) 33
^{13}C NMR of 2-(4-methylphenyl)-5-nitro-1-phenethyl-1*H*-indole (**24**) 33

^{1}H NMR of 1-benzyl-2-(4-methoxyphenyl)-5-nitro-1*H*-indole (**25**) 34
^{13}C NMR of 1-benzyl-2-(4-methoxyphenyl)-5-nitro-1*H*-indole (**25**) 34

^{1}H NMR of 1-(3-methoxybenzyl)-2-(4-methoxyphenyl)-5-nitro-1*H*-indole (**26**) 35
^{13}CNMR of 1-(3-methoxybenzyl)-2-(4-methoxyphenyl)-5-nitro-1*H*-indole (**26**) 35

^{1}H NMR of 1-(2-chlorophenyl)-2-(4-methoxyphenyl)-5-nitro-1*H*-indole (**27**) 36
^{13}C NMR of 1-(2-chlorophenyl)-2-(4-methoxyphenyl)-5-nitro-1*H*-indole (**27**) 36

^{1}H NMR of 2-(4-methoxyphenyl)-5-nitro-1-(3-(trifluoromethyl)benzyl)-1*H*-indole (**28**) 37
^{13}C NMR of 2-(4-methoxyphenyl)-5-nitro-1-(3-(trifluoromethyl)benzyl)-1*H*-indole (**28**) 37
^{19}F NMR of 2-(4-methoxyphenyl)-5-nitro-1-(3-(trifluoromethyl)benzyl)-1*H*-indole (**28**)........ 38

^{1}H NMR of 2-(4-Methoxyphenyl)-5-nitro-1-phenethyl-1*H*-indole (**29**) 39
^{13}C NMR of 2-(4-Methoxyphenyl)-5-nitro-1-phenethyl-1*H*-indole (**29**) 39

^{1}H NMR of 1-hexyl-2-(4-methoxyphenyl)-5-nitro-1*H*-indole (**30**) 40
^{13}C NMR of 1-hexyl-2-(4-methoxyphenyl)-5-nitro-1*H*-indole (**30**) 40

^{1}H NMR of 1-benzyl-2-(4-fluorophenyl)-5-nitro-1*H*-indole (**31**) 41
^{13}C NMR of 1-benzyl-2-(4-fluorophenyl)-5-nitro-1*H*-indole (**31**) 41

^{19}F NMR of 1-benzyl-2-(4-fluorophenyl)-5-nitro-1*H*-indole (**31**) 42

^{1}H NMR of 2-(4-fluorophenyl)-5-nitro-1-(3-(trifluoromethyl)benzyl)-1*H*-indole (**32**) 43
^{13}C NMR of 2-(4-fluorophenyl)-5-nitro-1-(3-(trifluoromethyl)benzyl)-1*H*-indole (**32**) 43
^{19}F NMR of 2-(4-fluorophenyl)-5-nitro-1-(3-(trifluoromethyl)benzyl)-1*H*-indole (**32**) 44

^{1}H NMR of 2-(4-fluorophenyl)-5-nitro-1-phenethyl-1*H*-indole (**33**) 45
^{13}C NMR of 2-(4-fluorophenyl)-5-nitro-1-phenethyl-1*H*-indole (**33**) 45
^{19}F NMR of 2-(4-fluorophenyl)-5-nitro-1-phenethyl-1*H*-indole (**33**) 46

^{1}H NMR of 1-isobutyl-2-(4-fluorophenyl)-5-nitro-1*H*-indole (**34**) 47
^{13}C NMR of 1-isobutyl-2-(4-fluorophenyl)-5-nitro-1*H*-indole (**34**) 47
^{19}F NMR of 1-isobutyl-2-(4-fluorophenyl)-5-nitro-1*H*-indole (**34**) 48

^{1}H NMR of 1-benzyl-2-(4-fluoro-3-methylphenyl)-5-nitro-1*H*-indole (**35**) 49
^{13}C NMR of 1-benzyl-2-(4-fluoro-3-methylphenyl)-5-nitro-1*H*-indole (**35**) 49
^{19}F NMR of 1-benzyl-2-(4-fluoro-3-methylphenyl)-5-nitro-1*H*-indole (**35**) 50

^{1}H NMR of 2-(4-fluoro-3-methylphenyl)-5-nitro-1-phenethyl-1*H*-indole (**36**) 51
^{13}C NMR of 2-(4-fluoro-3-methylphenyl)-5-nitro-1-phenethyl-1*H*-indole (**36**) 51
^{19}F NMR of 2-(4-fluoro-3-methylphenyl)-5-nitro-1-phenethyl-1*H*-indole (**36**) 52

^{1}H NMR of 2-(4-fluoro-3-methylphenyl)-1-(2-fluorophenethyl)-5-nitro-1H-indole (**37**) 53
^{13}C NMR of 2-(4-fluoro-3-methylphenyl)-1-(2-fluorophenethyl)-5-nitro-1H-indole (**37**) 53
^{19}F NMR of 2-(4-fluoro-3-methylphenyl)-1-(2-fluorophenethyl)-5-nitro-1H-indole (**37**) 54

^{1}H NMR of 1-Benzyl-2-(4-chlorophenyl)-5-nitro-1*H*-indole (**38**) 55
^{13}C NMR of 1-Benzyl-2-(4-chlorophenyl)-5-nitro-1*H*-indole (**38**) 55

^{1}H NMR of 2-(4-chlorophenyl)-5-nitro-1-(3-(trifluoromethyl)benzyl)-1*H*-indole (**39**) 56
^{13}C NMR of 2-(4-chlorophenyl)-5-nitro-1-(3-(trifluoromethyl)benzyl)-1*H*-indole (**39**) 56
^{19}F NMR of 2-(4-chlorophenyl)-5-nitro-1-(3-(trifluoromethyl)benzyl)-1*H*-indole (**39**) 57

^{1}H NMR of 2-(4-chlorophenyl)-5-nitro-1-phenethyl-1*H*-indole (**40**) 58
^{13}C NMR of 2-(4-chlorophenyl)-5-nitro-1-phenethyl-1*H*-indole (**40**) 58

^{1}H NMR of 1-benzyl-2-(2,3-dihydrobenzo[*b*][1,4]dioxin-6-yl-)-5-nitro-1*H*-indole (**41**) 59
^{13}C NMR of 1-benzyl-2-(2,3-dihydrobenzo[*b*][1,4]dioxin-6-yl-)-5-nitro-1*H*-indole (**41**) 59

^{1}H NMR of 2-(2,3-dihydobenzo[*b*][1,4]dioxin-6-yl)-1-(2-fluorophenyl)-5-nitro-1*H*-indole (**42**) 60
^{13}C NMR of 2-(2,3-dihydobenzo[*b*][1,4]dioxin-6-yl)-1-(2-fluorophenyl)-5-nitro-1*H*-indole (**42**) 60
^{19}F NMR of 2-(2,3-dihydobenzo[*b*][1,4]dioxin-6-yl)-1-(2-fluorophenyl)-5-nitro-1*H*-indole (**42**) 61

^{1}H NMR of 2-(2,3-dihydobenzo[*b*][1,4]dioxin-6-yl)-5-nitro-1-phenethyl-1*H*-indole (**43**) 62
^{13}C NMR of 2-(2,3-dihydobenzo[*b*][1,4]dioxin-6-yl)-5-nitro-1-phenethyl-1*H*-indole (**43**) 62

^{1}H NMR of 2-(2-fluoro-5-nitrophenyl)-1-phenylethan-1-one (**5**)

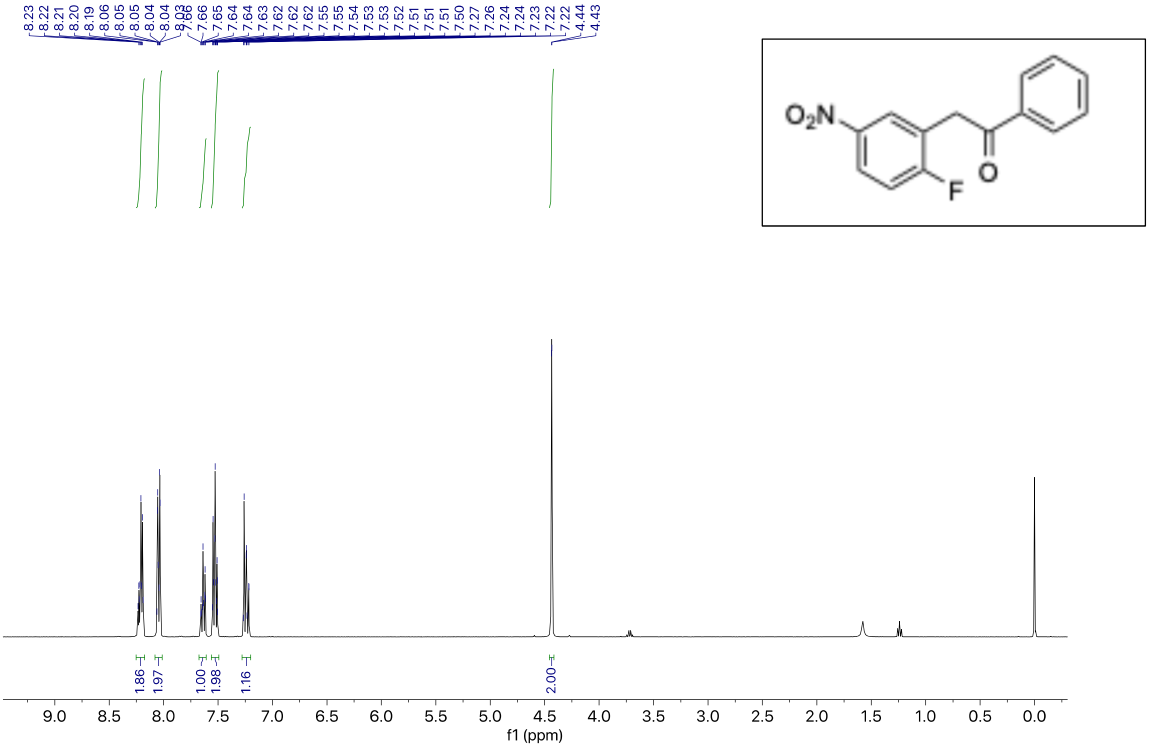

^{13}C NMR of 2-(2-fluoro-5-nitrophenyl)-1-phenylethan-1-one (**5**)

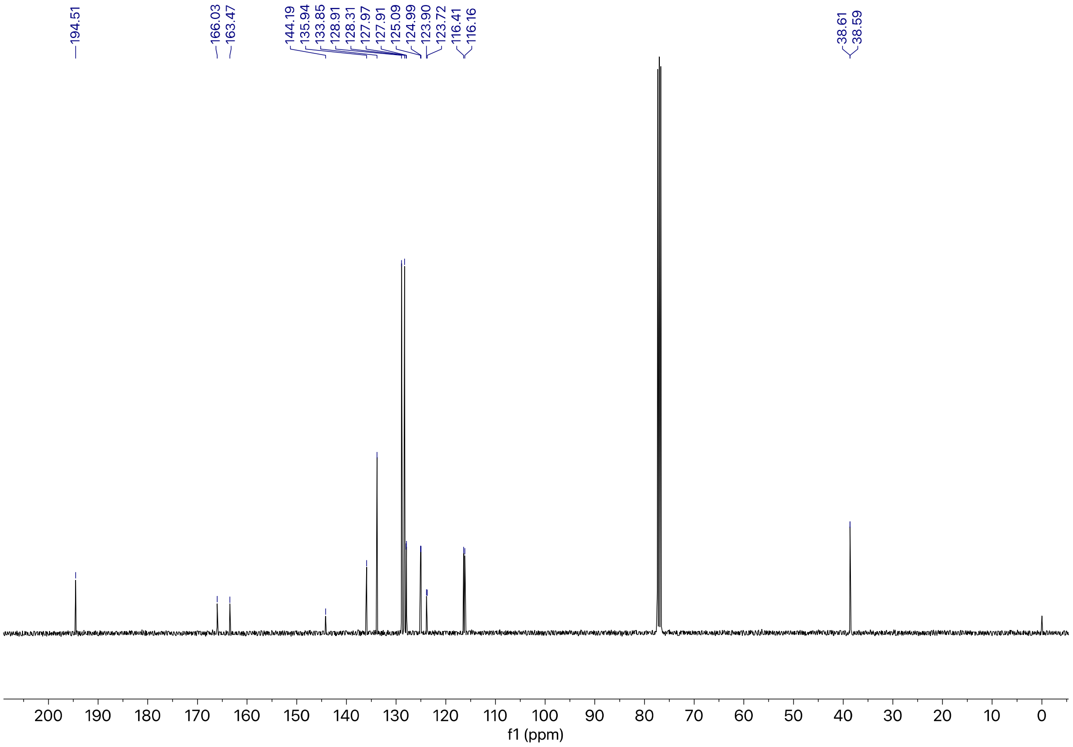

^{19}F NMR of 2-(2-fluoro-5-nitrophenyl)-1-phenylethan-1-one (**5**)

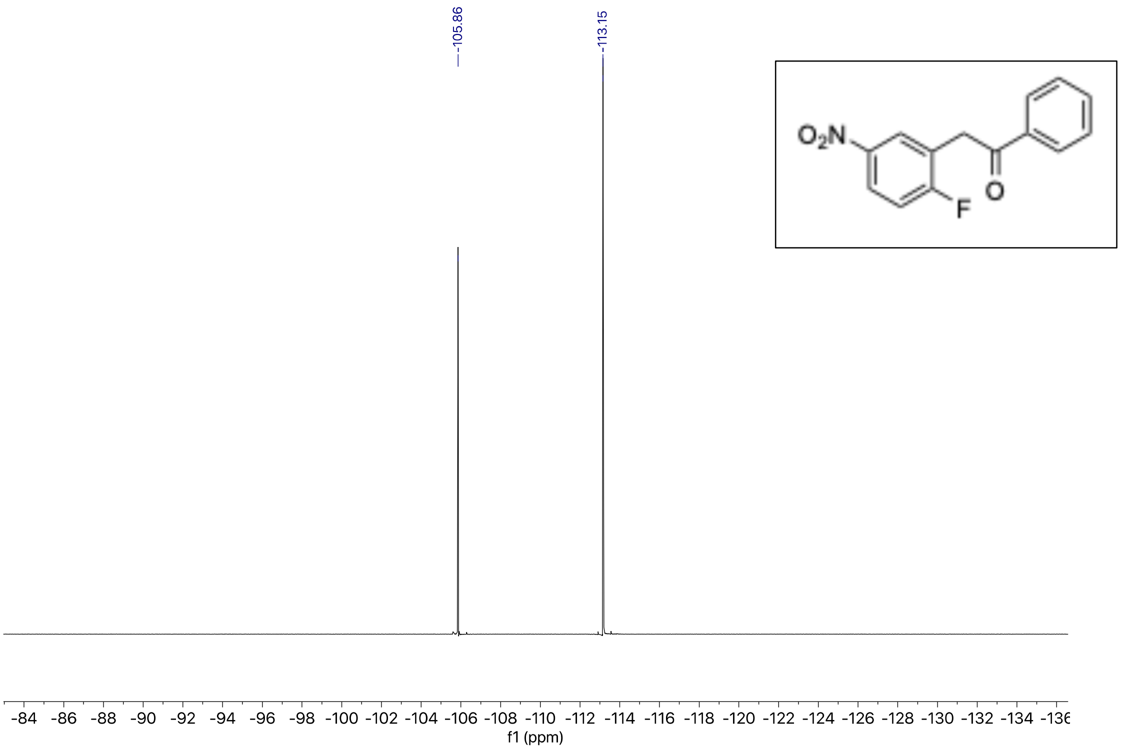

^{1}H NMR of 2-(2-fluoro-5-nitrophenyl)-1-(4-methylphenyl)ethan-1-one (**6**)

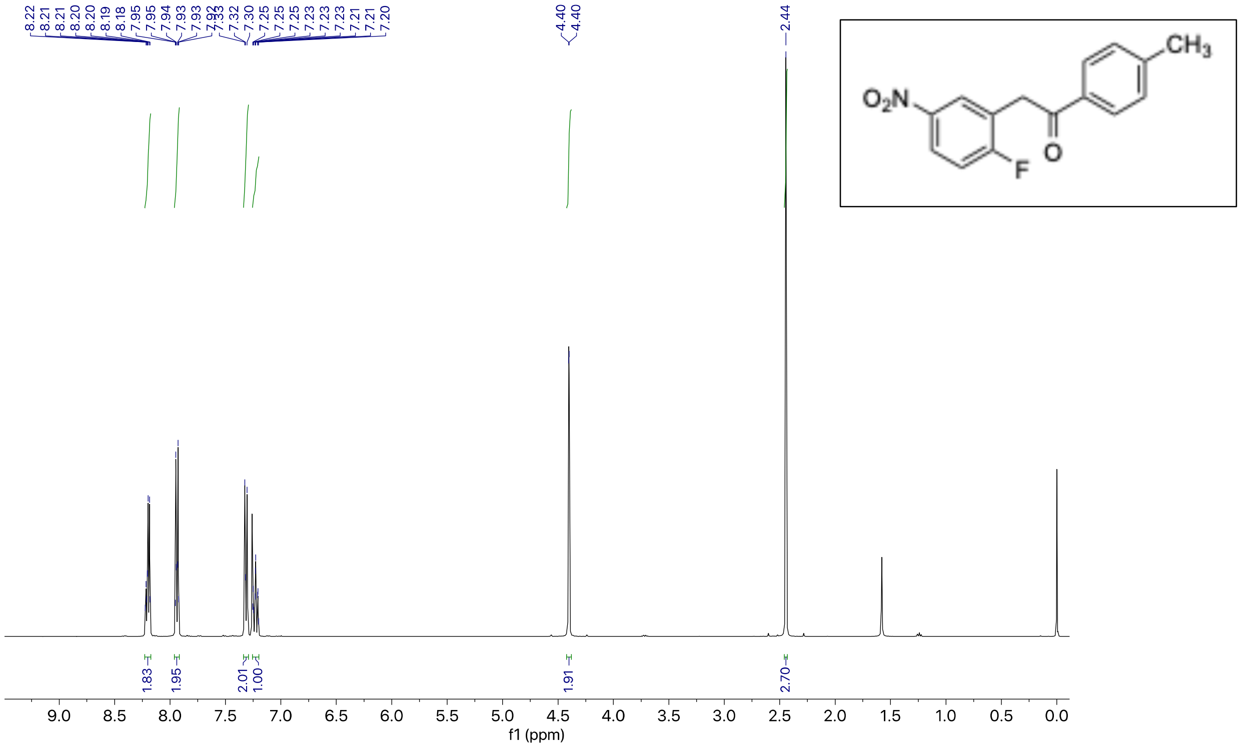

^{13}C NMR of 2-(2-fluoro-5-nitrophenyl)-1-(4-methylphenyl)ethan-1-one (**6**)

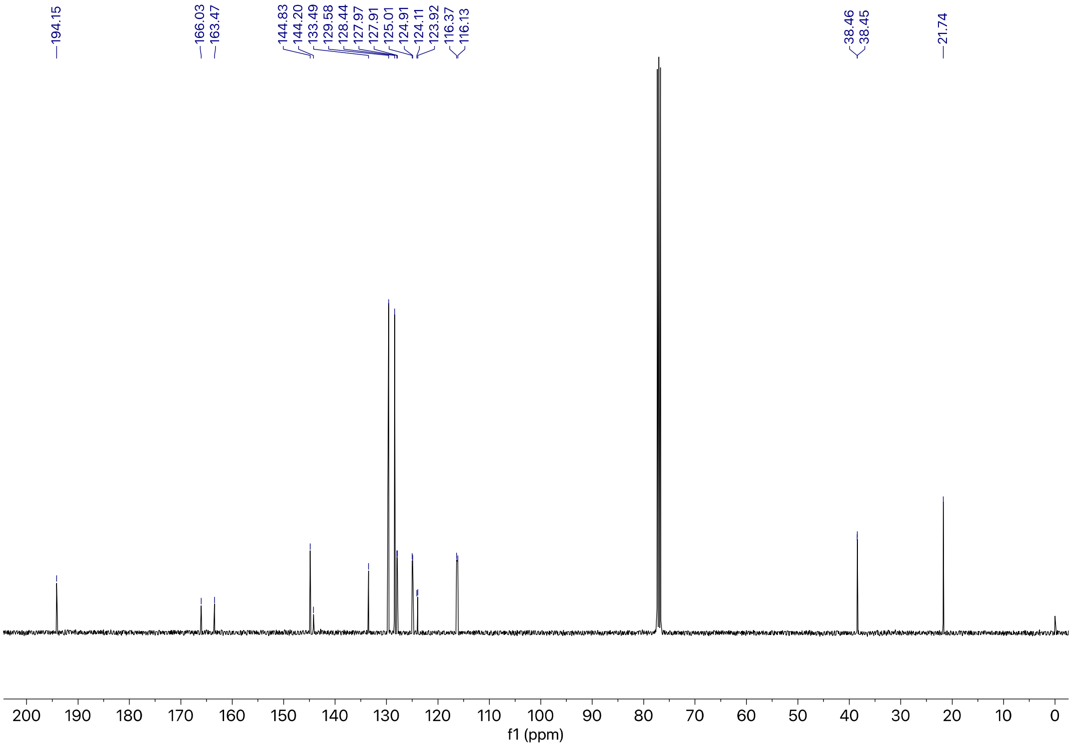

^{19}F NMR of 2-(2-fluoro-5-nitrophenyl)-1-(4-methylphenyl)ethan-1-one (**6**)

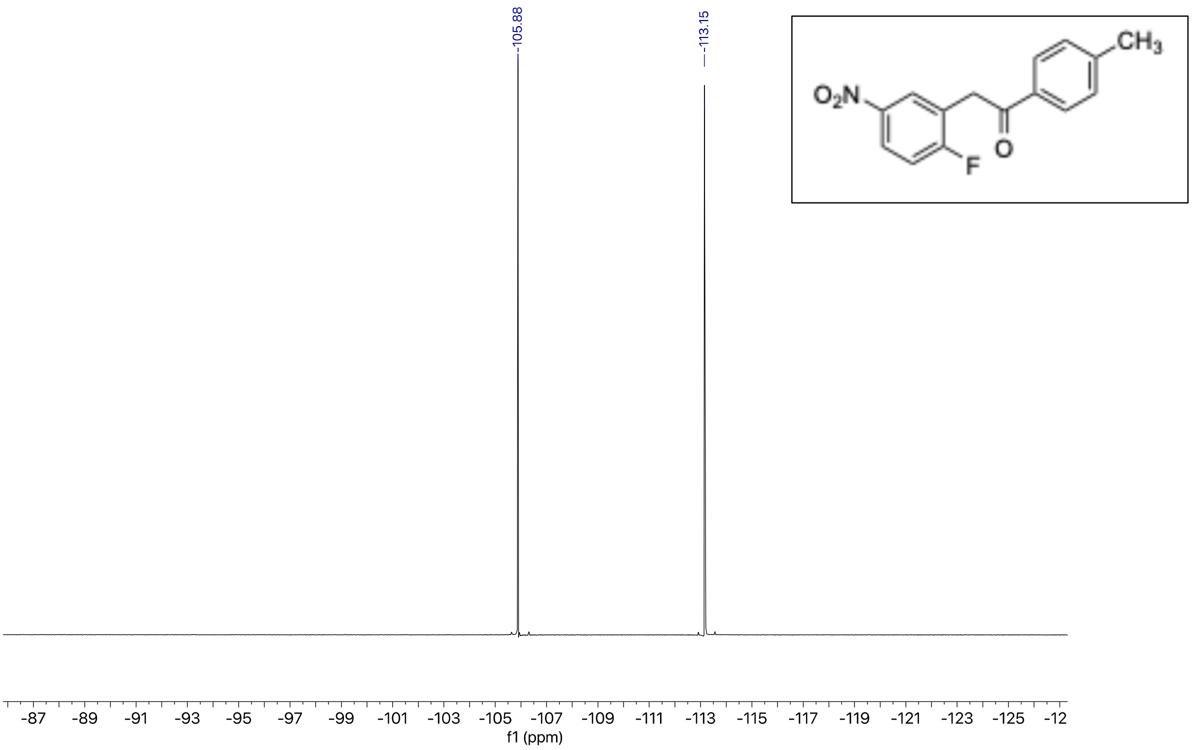

^{1}H NMR of 2-(2-fluoro-5-nitrophenyl)-1-(4-methoxyphenyl)ethan-1-one (**7**)

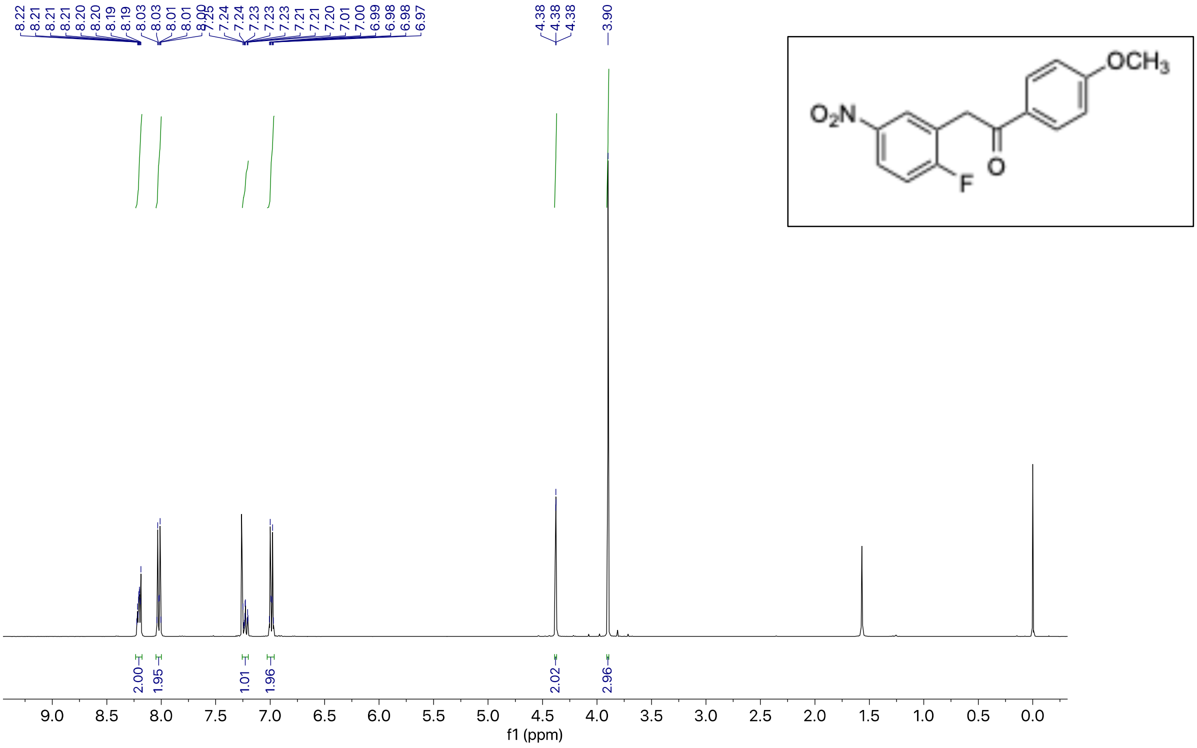

^{13}C NMR of 2-(2-fluoro-5-nitrophenyl)-1-(4-methoxyphenyl)ethan-1-one (**7**)

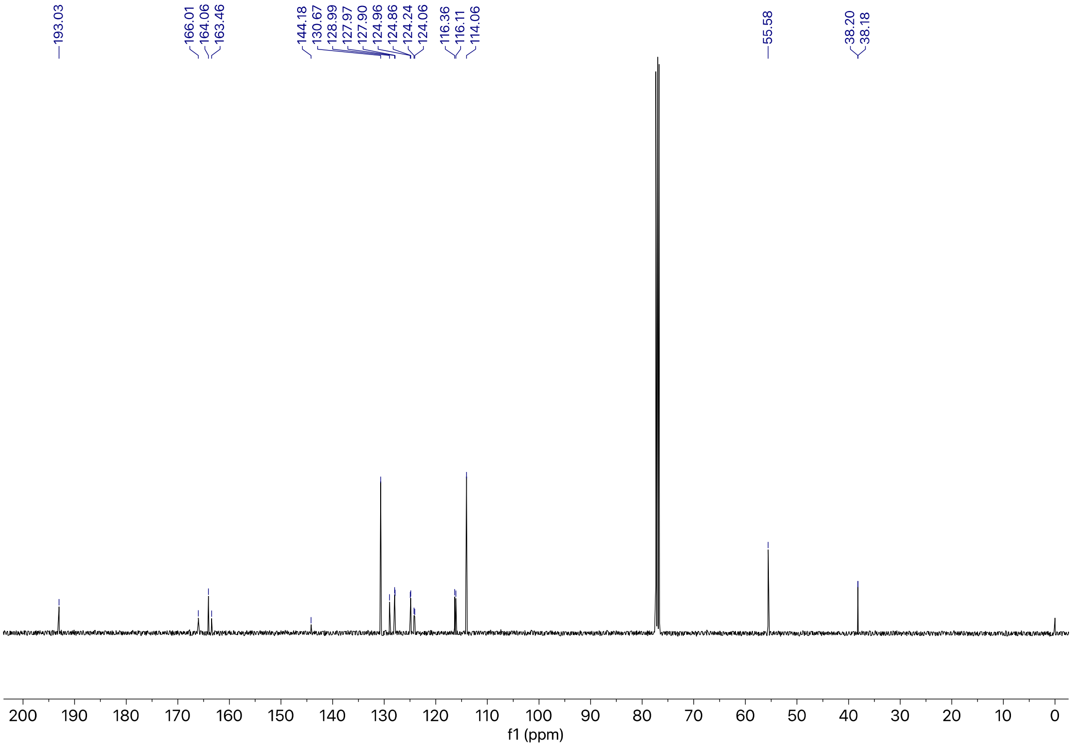

^{19}F NMR of 2-(2-fluoro-5-nitrophenyl)-1-(4-methoxyphenyl)ethan-1-one (**7**)

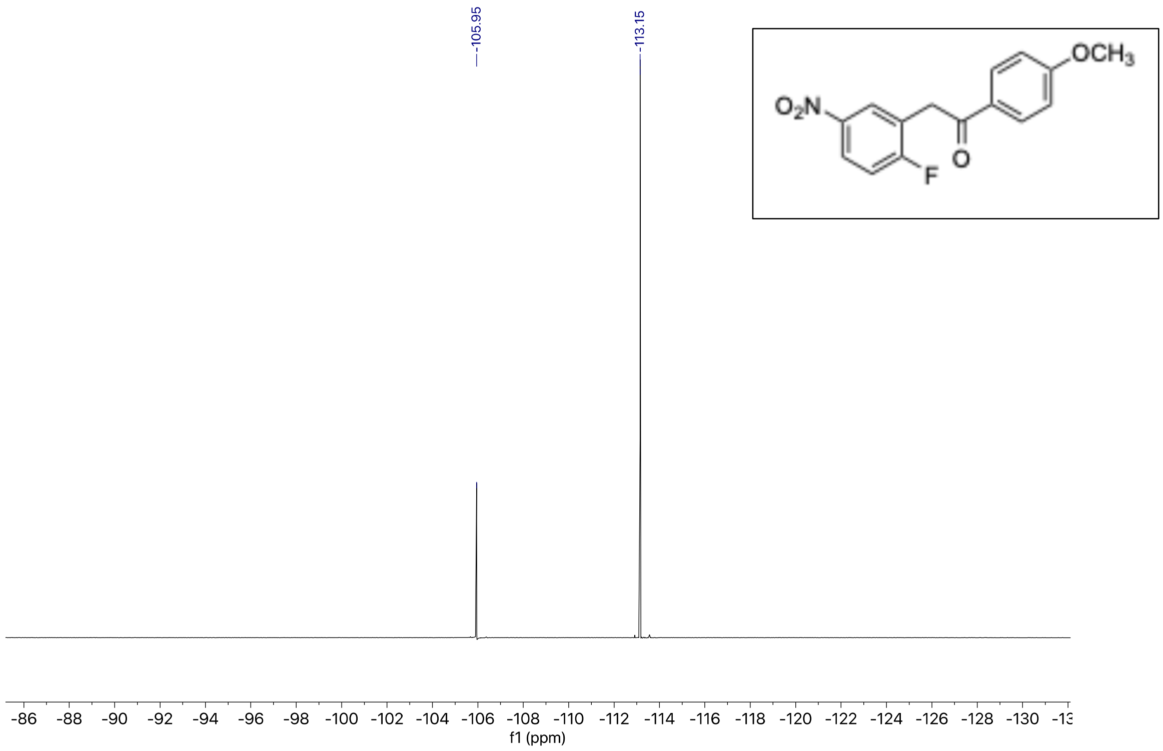

^{1}H NMR of 2-(2-fluoro-5-nitrophenyl)-1-(4-fluorophenyl)ethan-1-one (**8**)

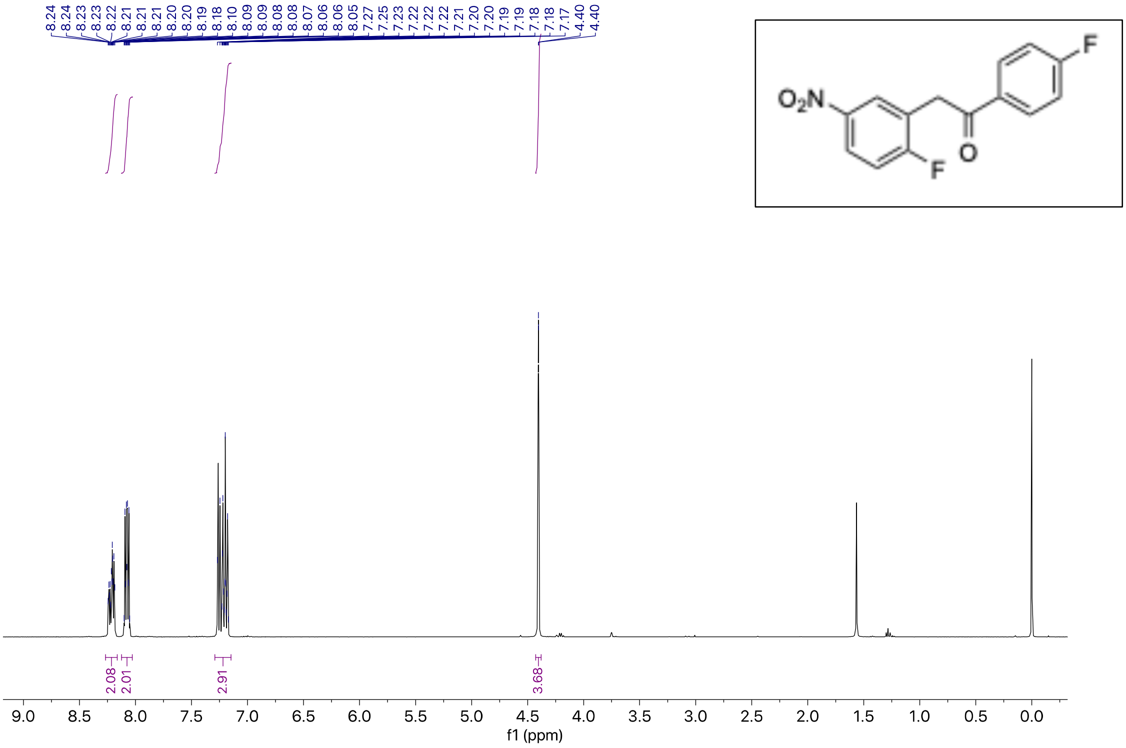

^{13}C NMR of 2-(2-fluoro-5-nitrophenyl)-1-(4-fluorophenyl)ethan-1-one (**8**)

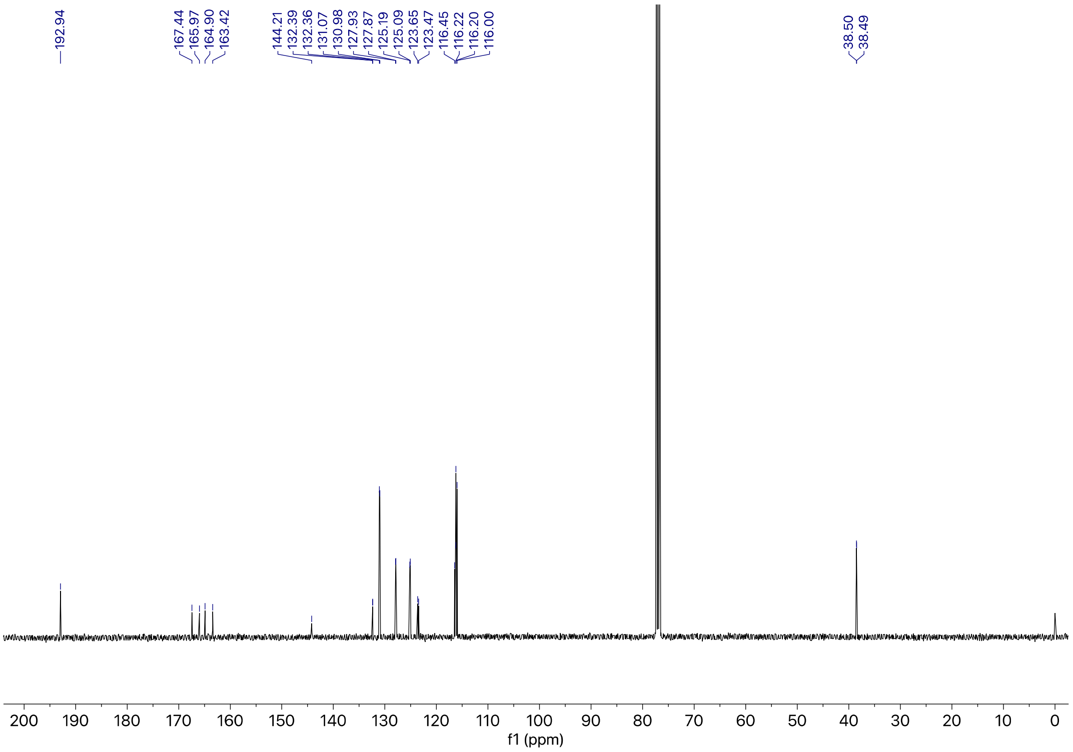

^{19}F NMR of 2-(2-fluoro-5-nitrophenyl)-1-(4-fluorophenyl)ethan-1-one (**8**)

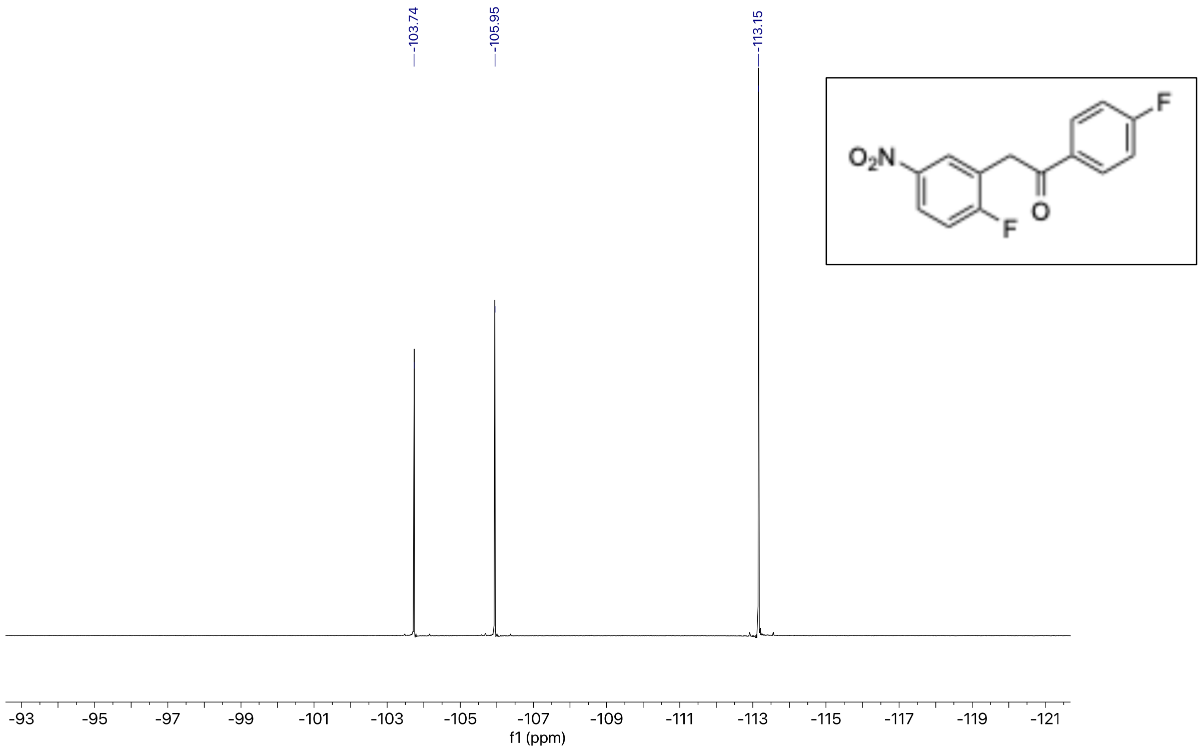

^{1}H NMR of 1-(4-fluoro-3-methylphenyl)- 2-(2-fluoro-5-nitrophenyl)ethan-1-one (**9**)

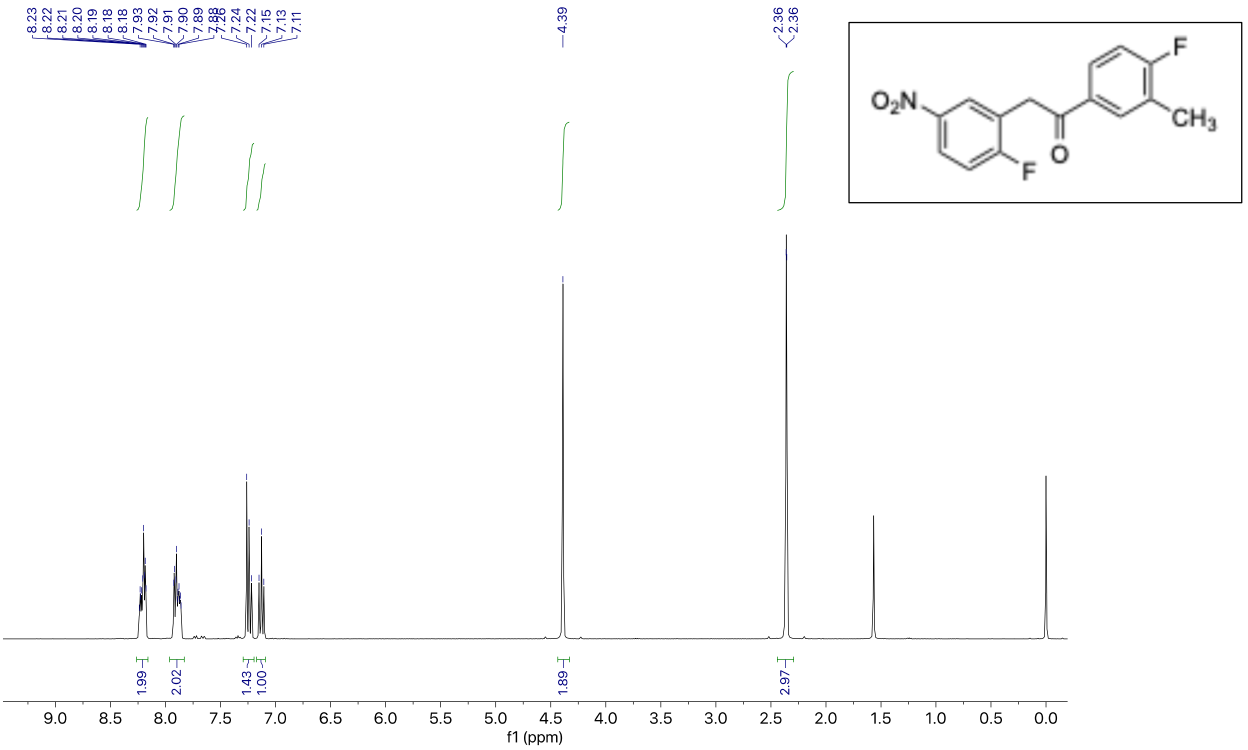

101 MHz ^{13}C NMR of 1-(4-fluoro-3-methylphenyl)- 2-(2-fluoro-5-nitrophenyl)ethan-1-one (**9**)

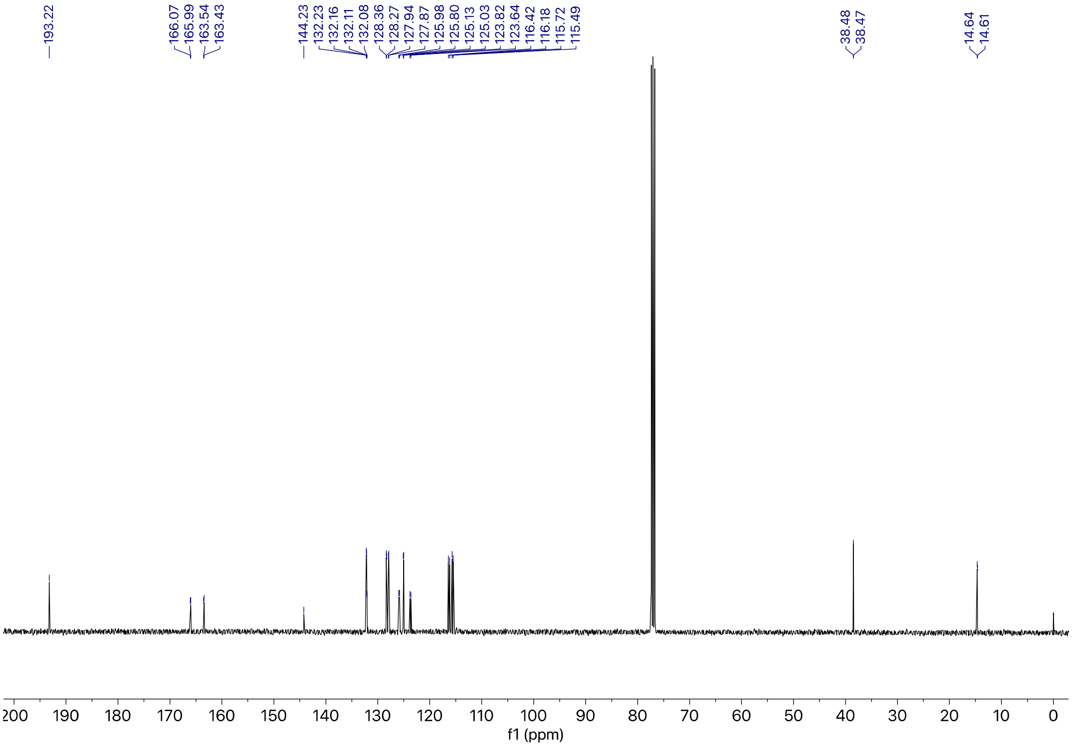

^{19}F NMR of 1-(4-fluoro-3-methylphenyl)- 2-(2-fluoro-5-nitrophenyl)ethan-1-one (**9**)

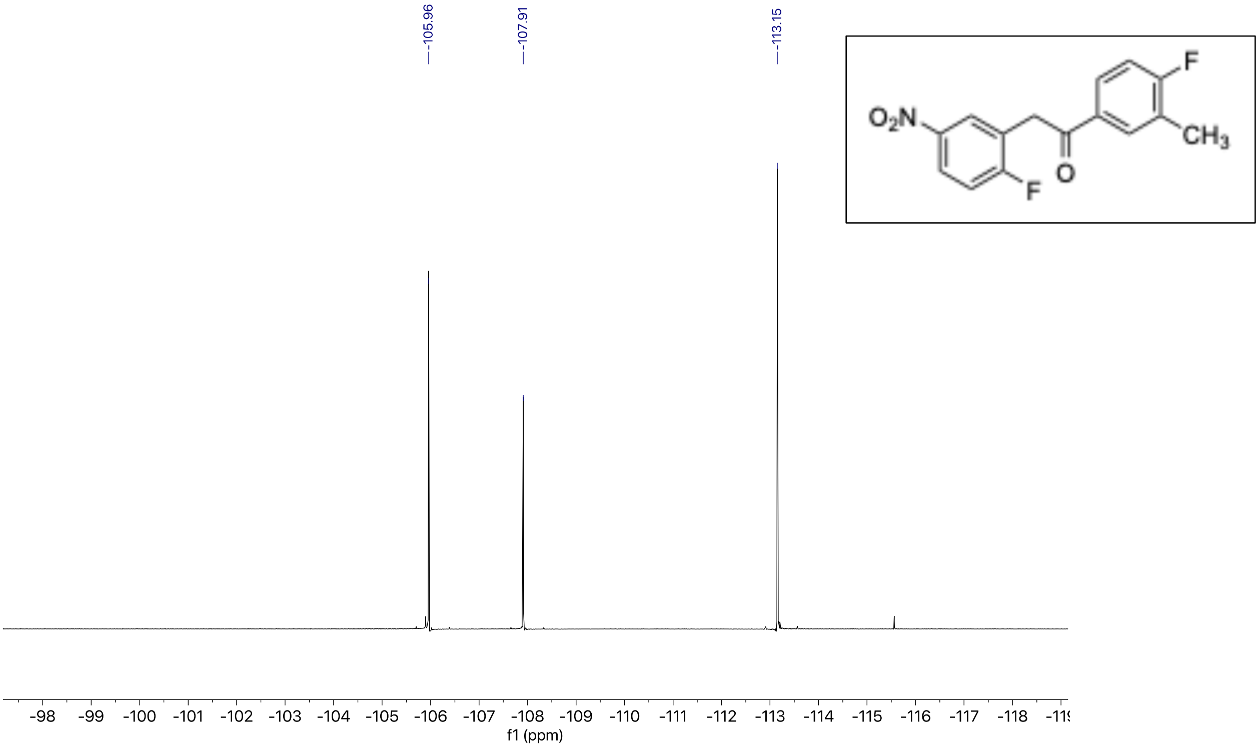

^{1}H NMR of 1-(4-chlorophenyl)-2-(2-fluoro-5-nitrophenyl)ethan-1-one (**10**)

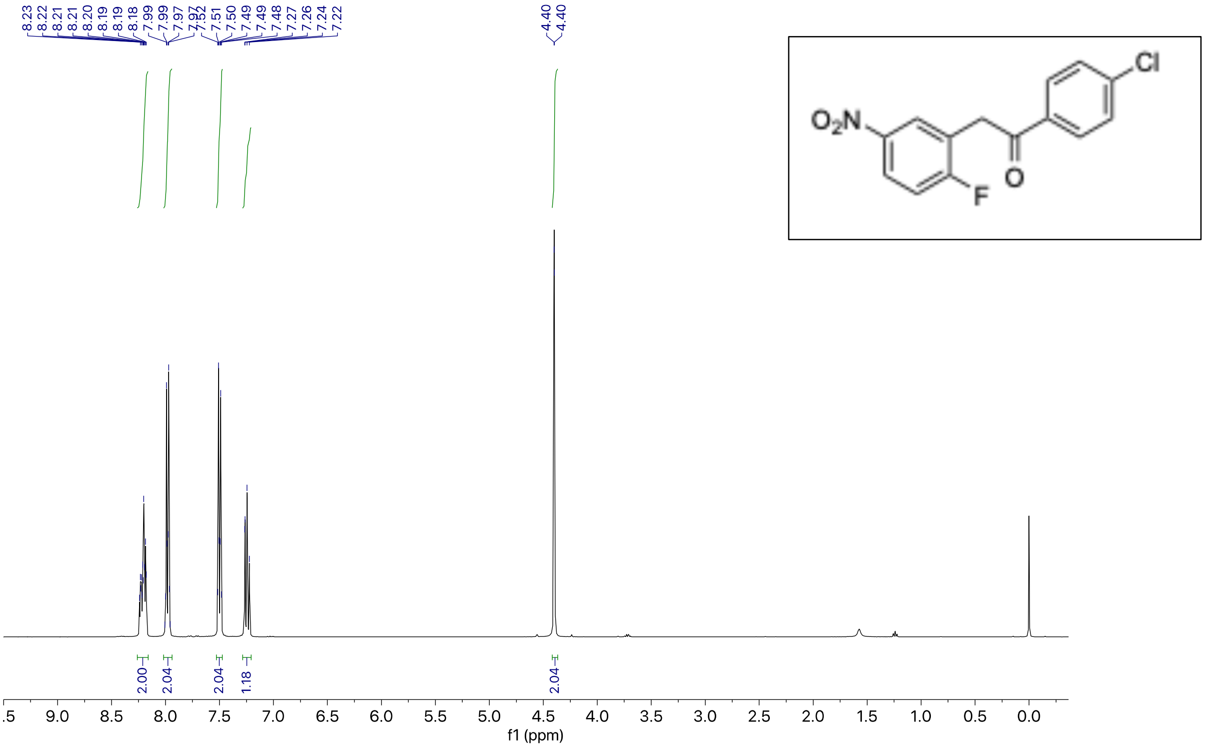

^{13}C NMR of 1-(4-chlorophenyl)-2-(2-fluoro-5-nitrophenyl)ethan-1-one (**10**)

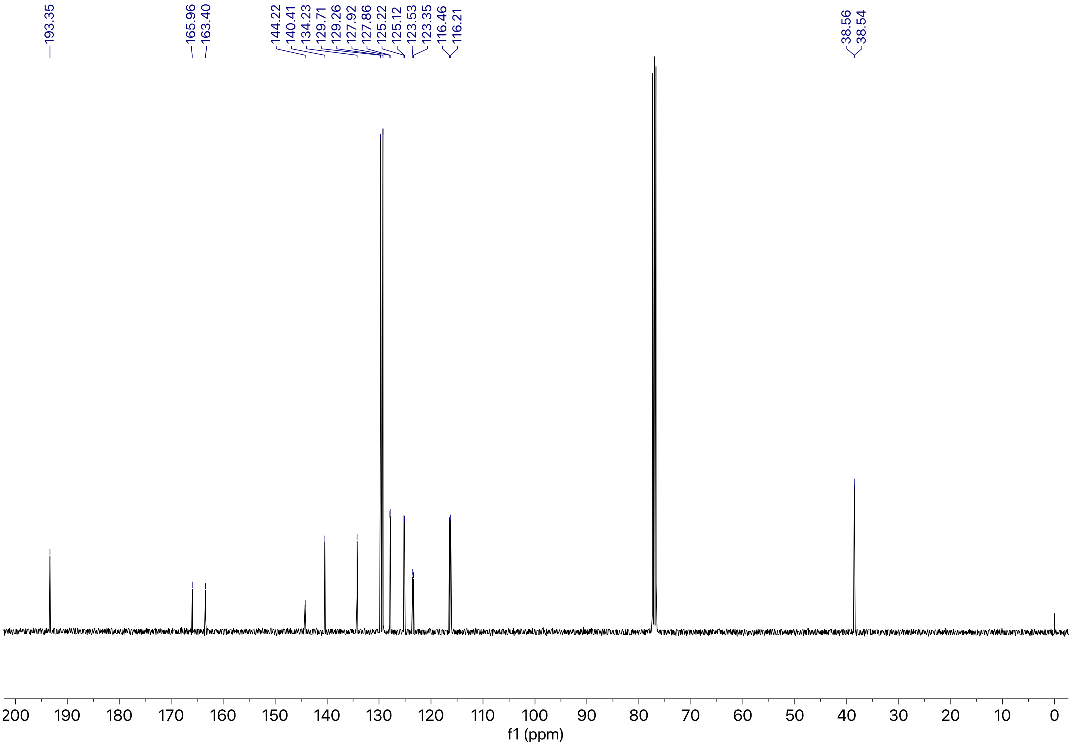

^{19}F NMR of 1-(4-chlorophenyl)-2-(2-fluoro-5-nitrophenyl)ethan-1-one (**10**)

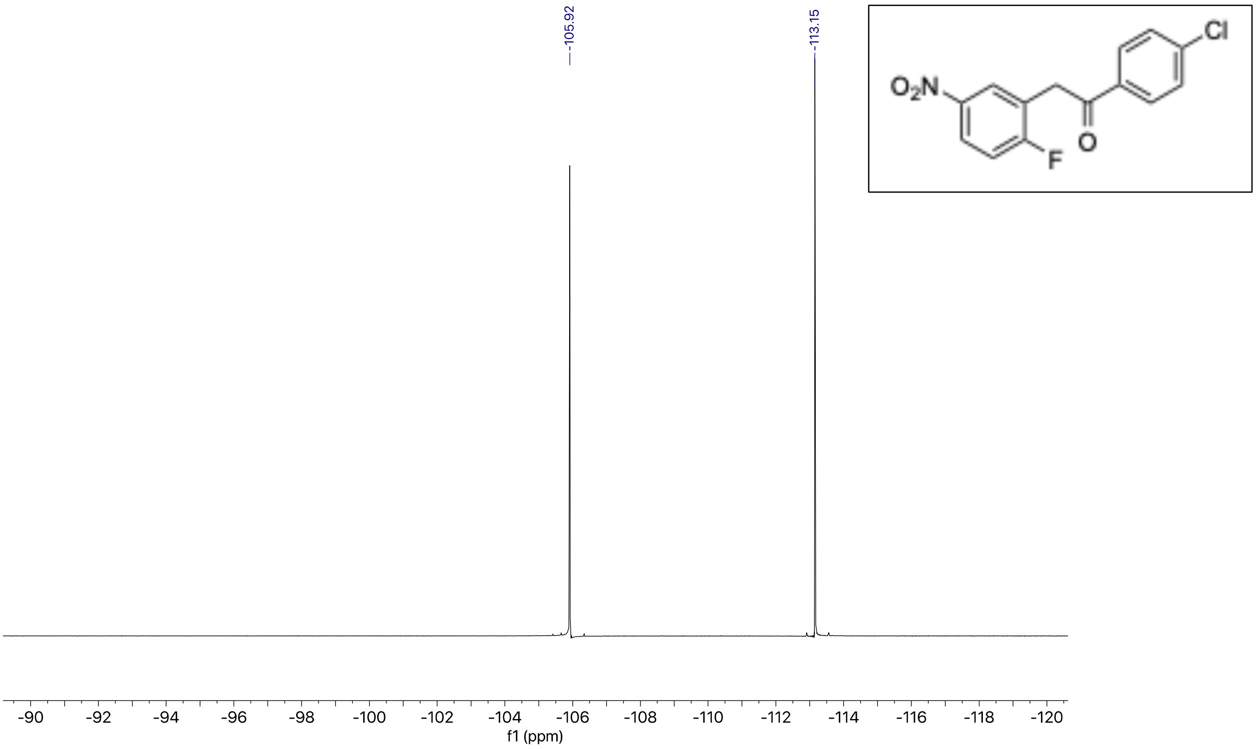

^{1}H NMR of 1-(2,3-dihydrobenzo[*b*][1,4]dioxin-6-yl)-2-(2-fluoro-5-nitrophenyl)ethan-1-one (**11**)

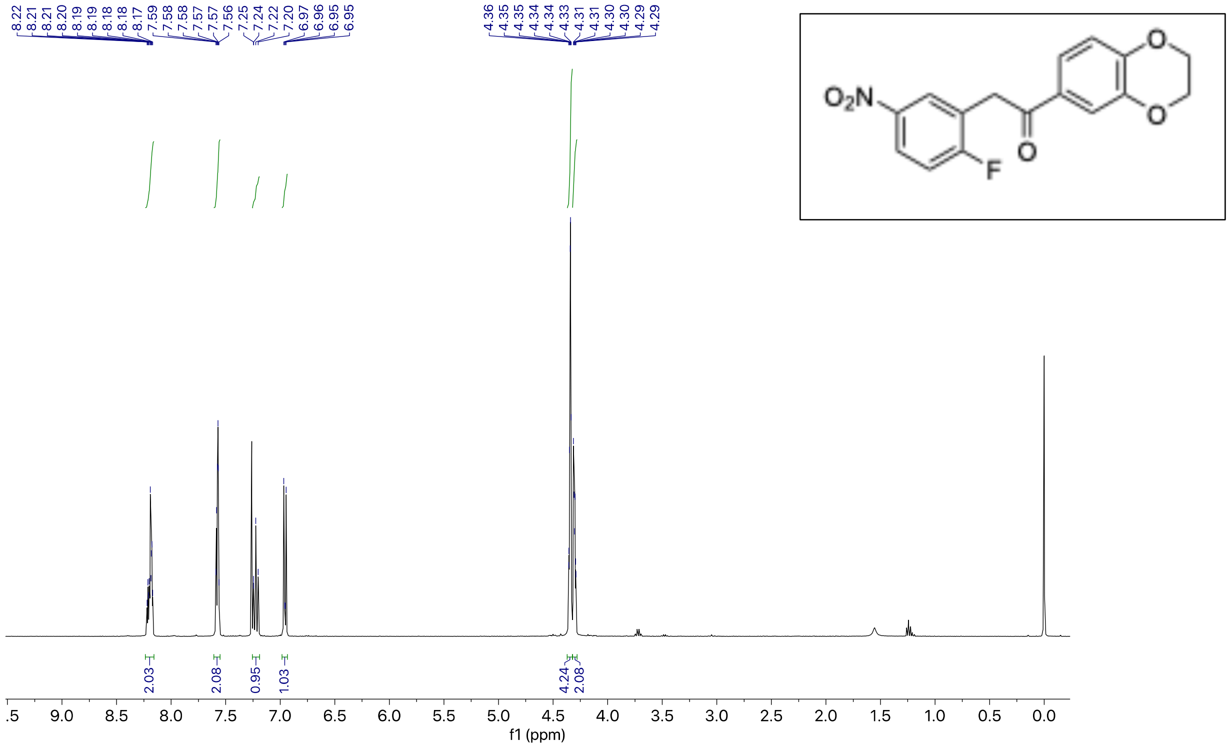

^{13}C NMR of 1-(2,3-dihydrobenzo[*b*][1,4]dioxin-6-yl)-2-(2-fluoro-5-nitrophenyl)ethan-1-one (**11**)

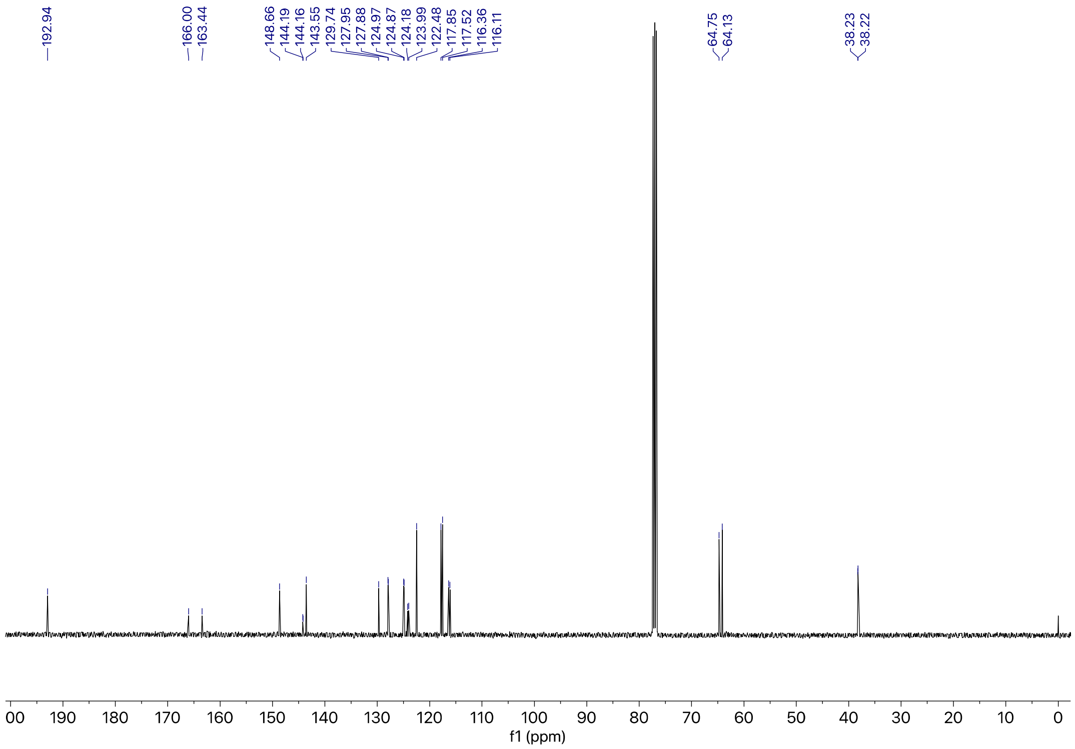

^{19}F NMR of 1-(2,3-dihydrobenzo[*b*][1,4]dioxin-6-yl)-2-(2-fluoro-5-nitrophenyl)ethan-1-one (**11**)

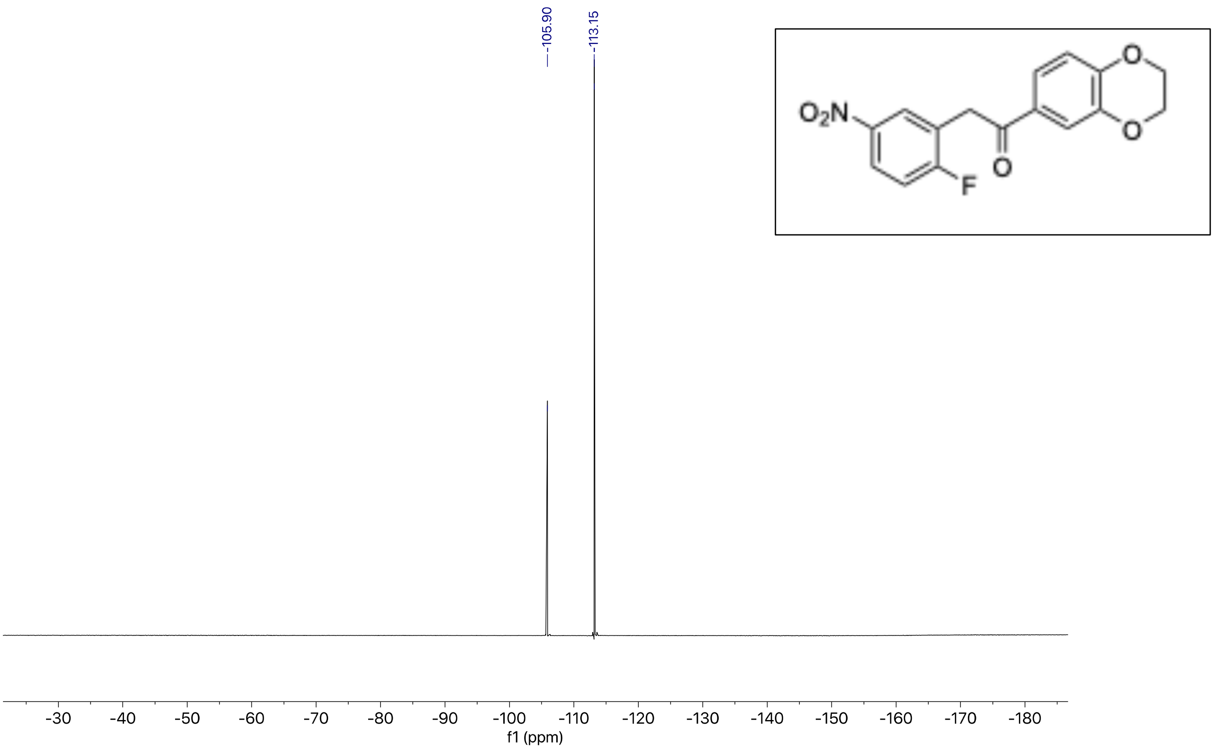

^{1}H NMR of 1-benzyl-5-nitro-2-phenyl-1*H*-indole (**12**)

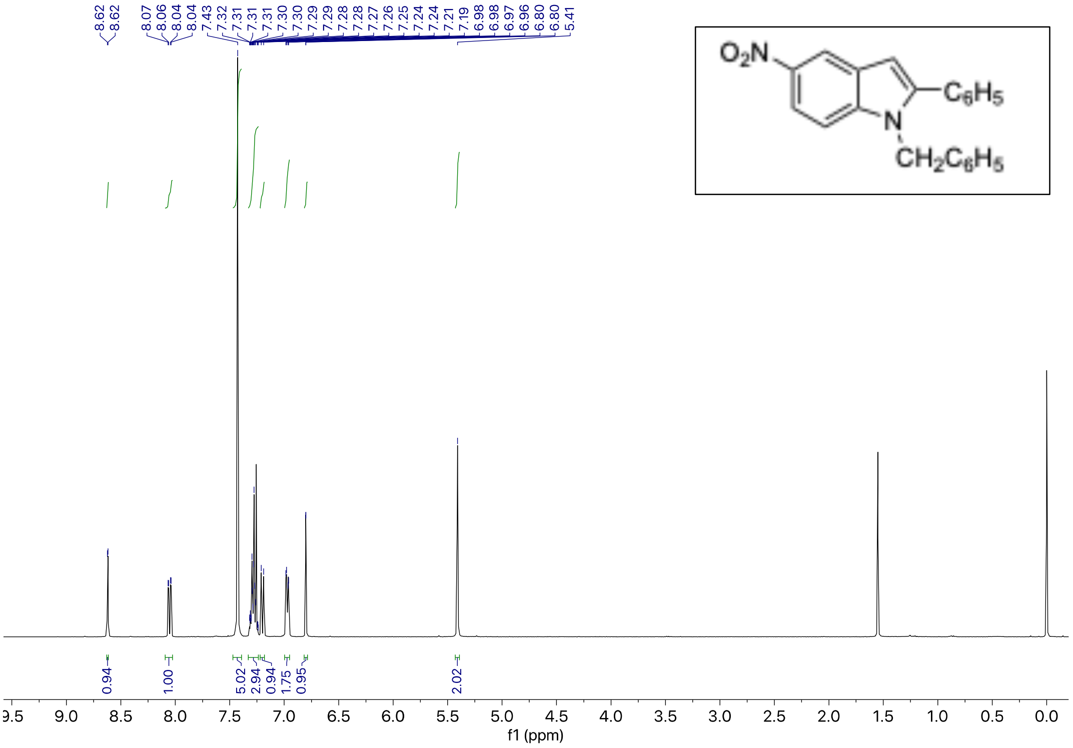

^{13}C NMR of 1-benzyl-5-nitro-2-phenyl-1*H*-indole (**12**)

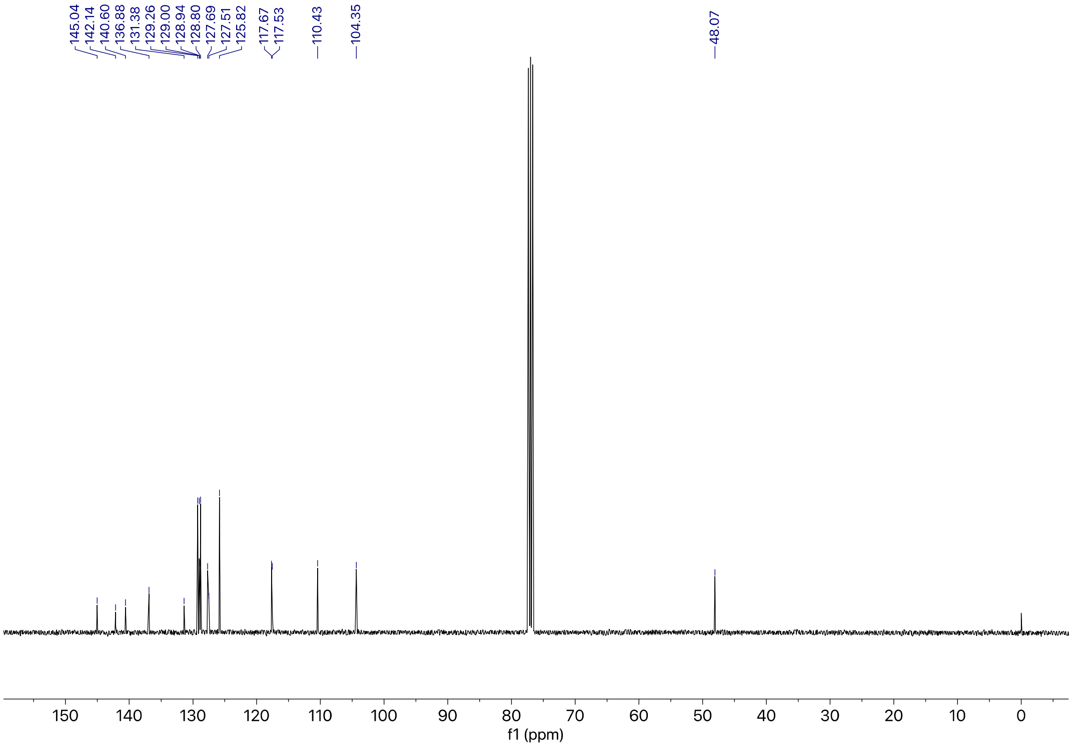

^{1}H NMR of 1-(3-methoxybenzyl)-5-nitro-2-phenyl-1*H*-indole (**13**)

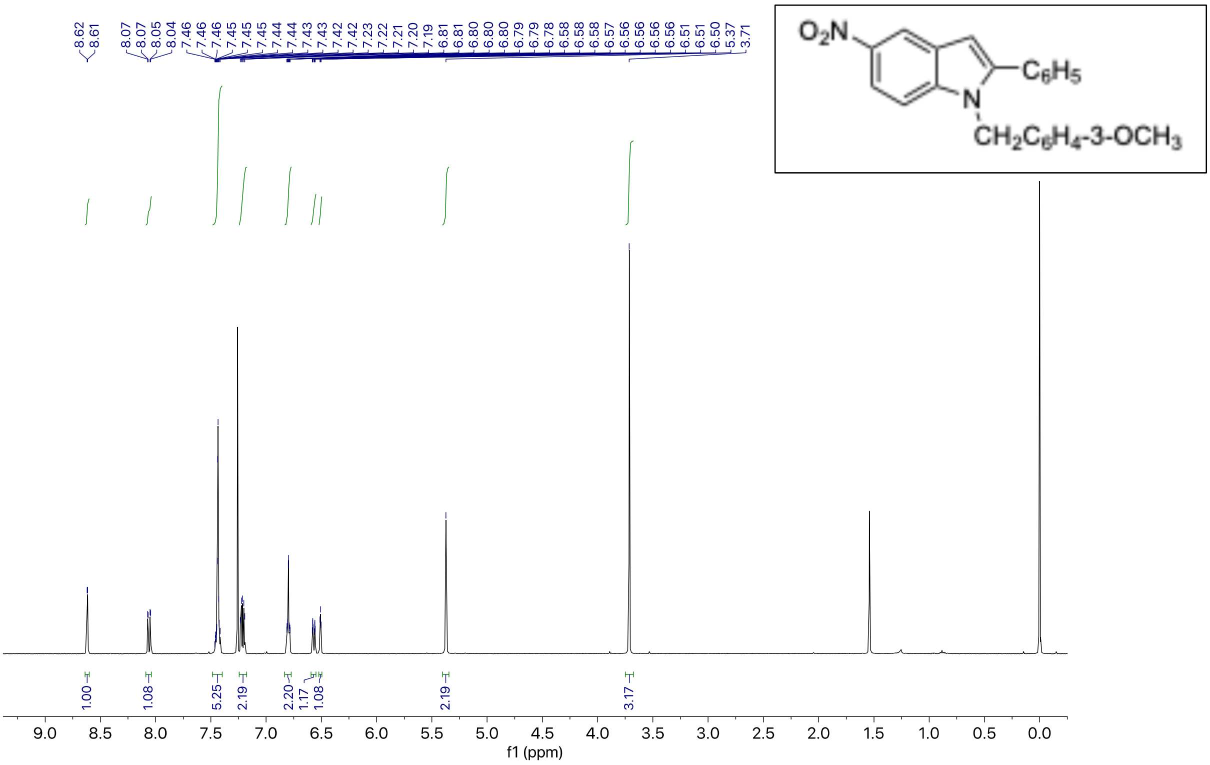

^{13}C NMR of 1-(3-methoxybenzyl)-5-nitro-2-phenyl-1*H*-indole (**13**)

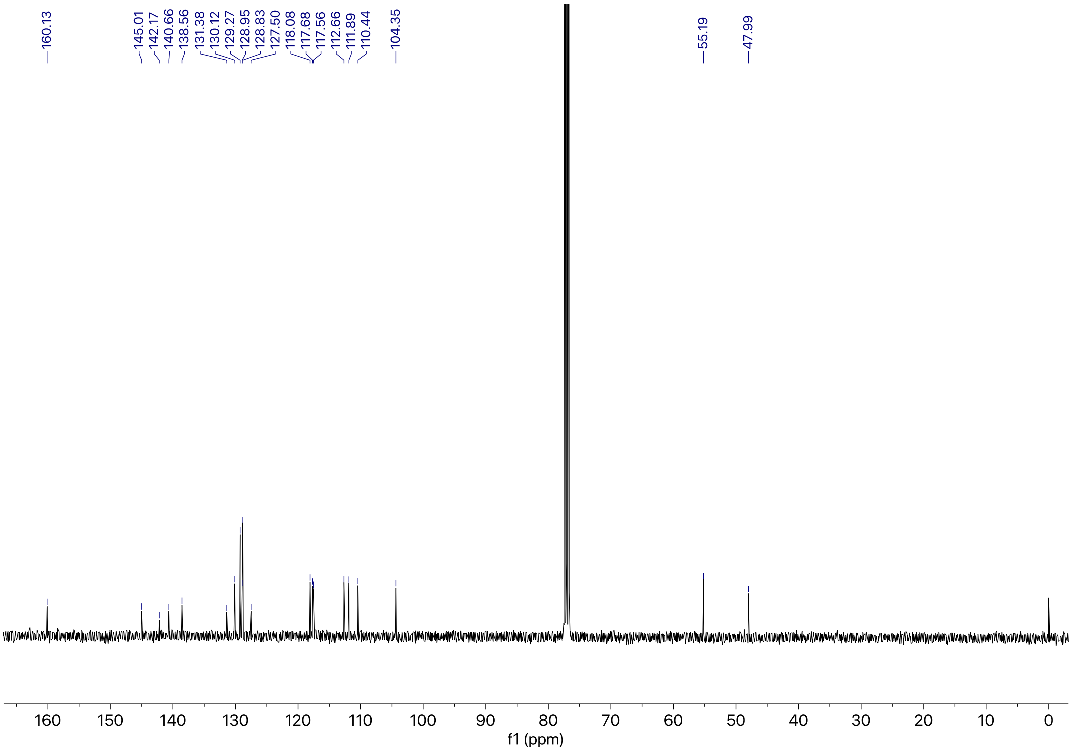

^{1}H NMR of 5-nitro-2-phenyl-1-(3-(trifluoromethyl)phenyl)-1*H*-indole (**14**)

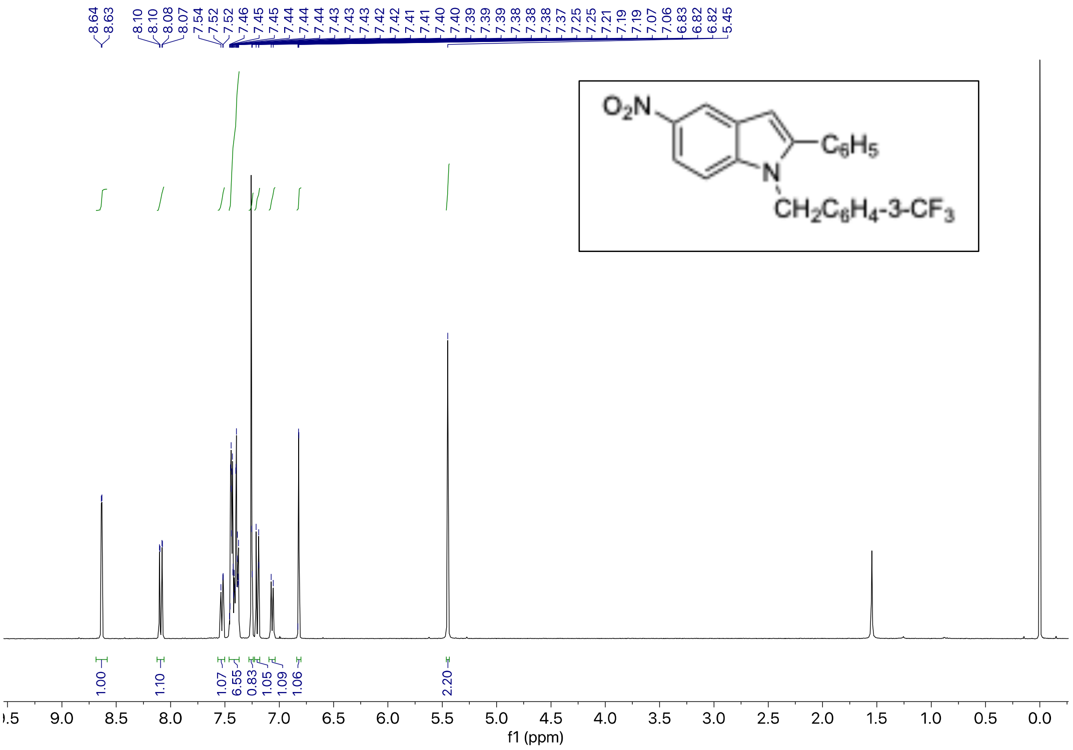

^{13}C NMR of 5-nitro-2-phenyl-1-(3-(trifluoromethyl)phenyl)-1*H*-indole (**14**)

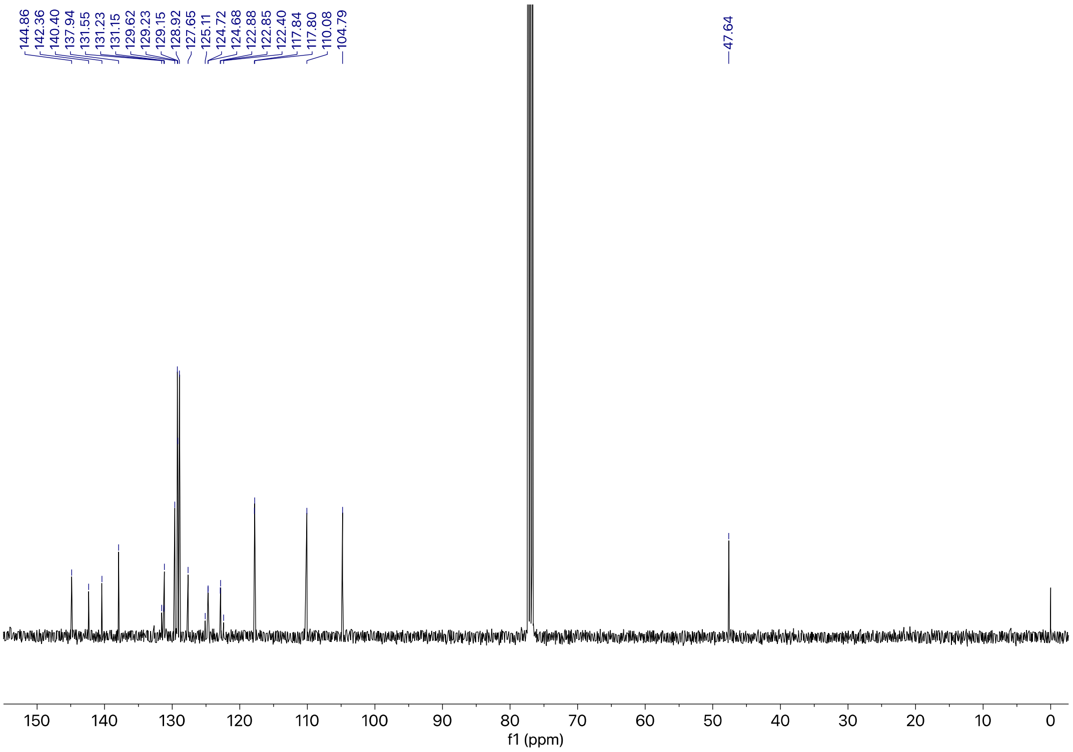

^{19}F NMR of 5-nitro-2-phenyl-1-(3-(trifluoromethyl)phenyl)-1*H*-indole (**14**)

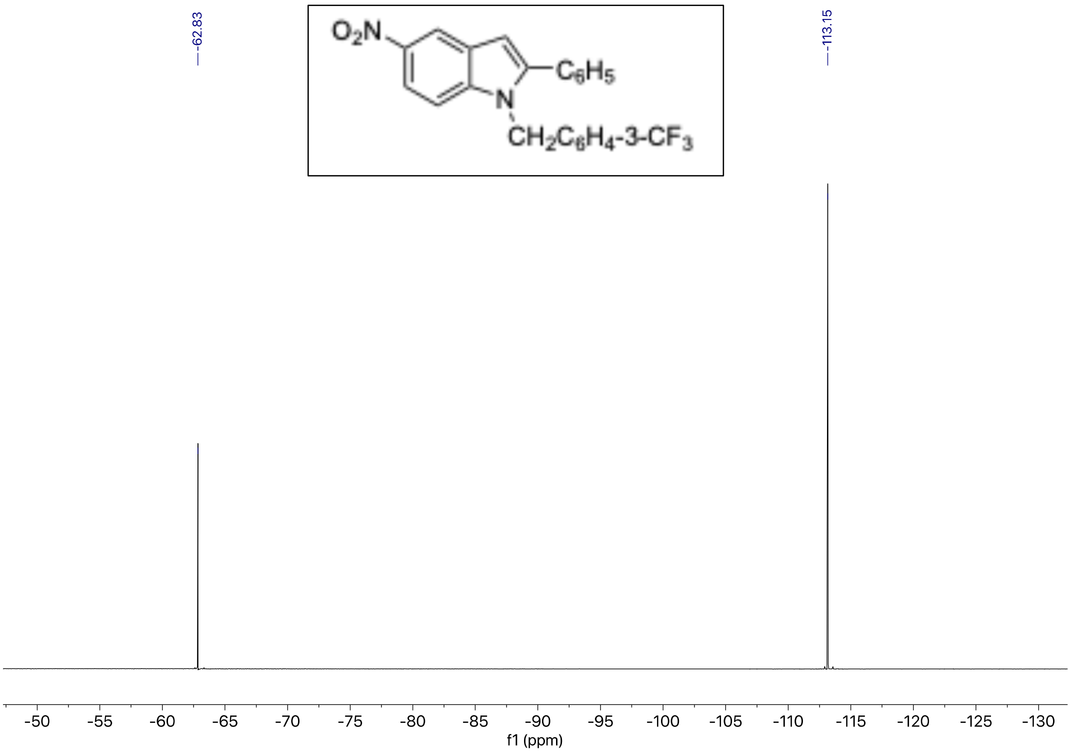

^{1}H NMR of 1-(2-fluorophenethyl)-5-nitro-2-phenyl-1*H*-indole (**15**)

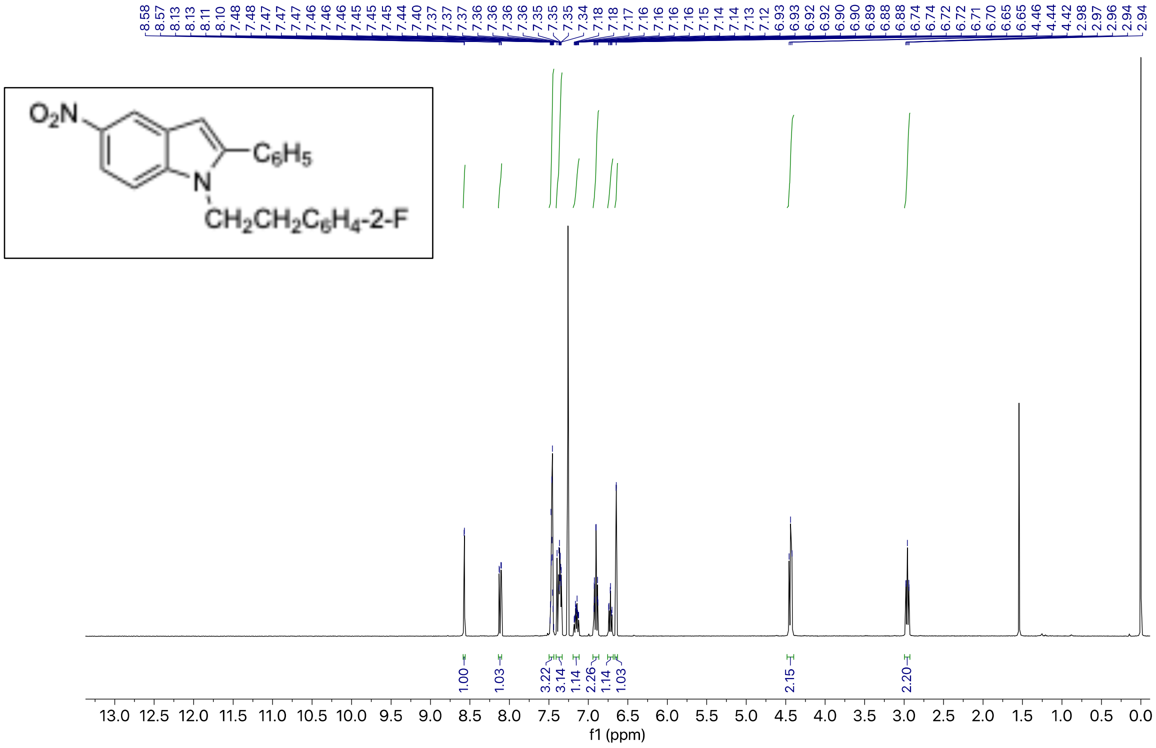

^{13}C NMR of 1-(2-fluorophenethyl)-5-nitro-2-phenyl-1*H*-indole (**15**)

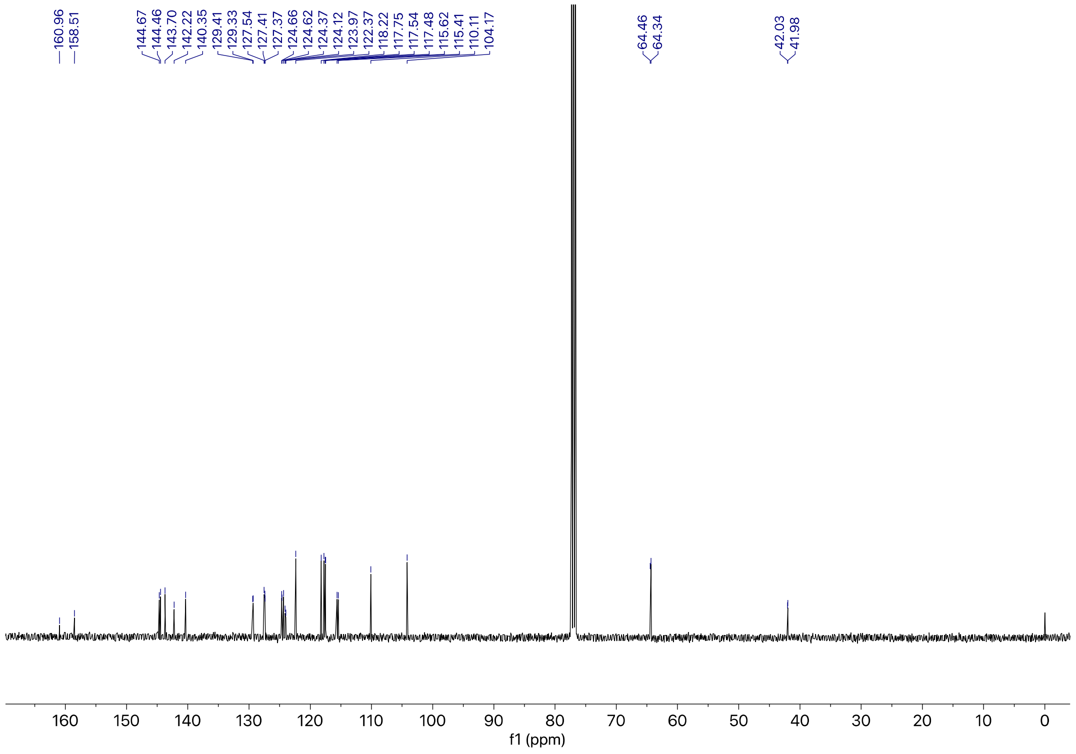

^{19}F NMR of 1-(2-fluorophenethyl)-5-nitro-2-phenyl-1*H*-indole (**15**)

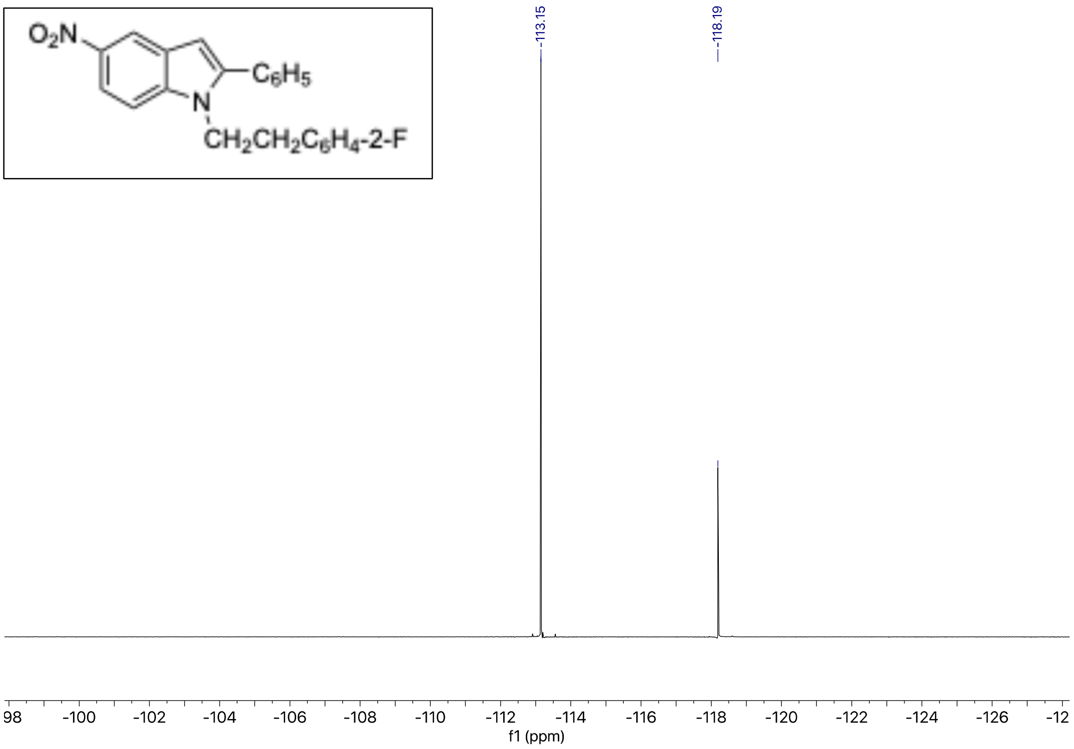

^{1}H NMR of 1-(3-isopropoxypropyl)-5-nitro-2-phenyl-1*H*-indole (**16**)

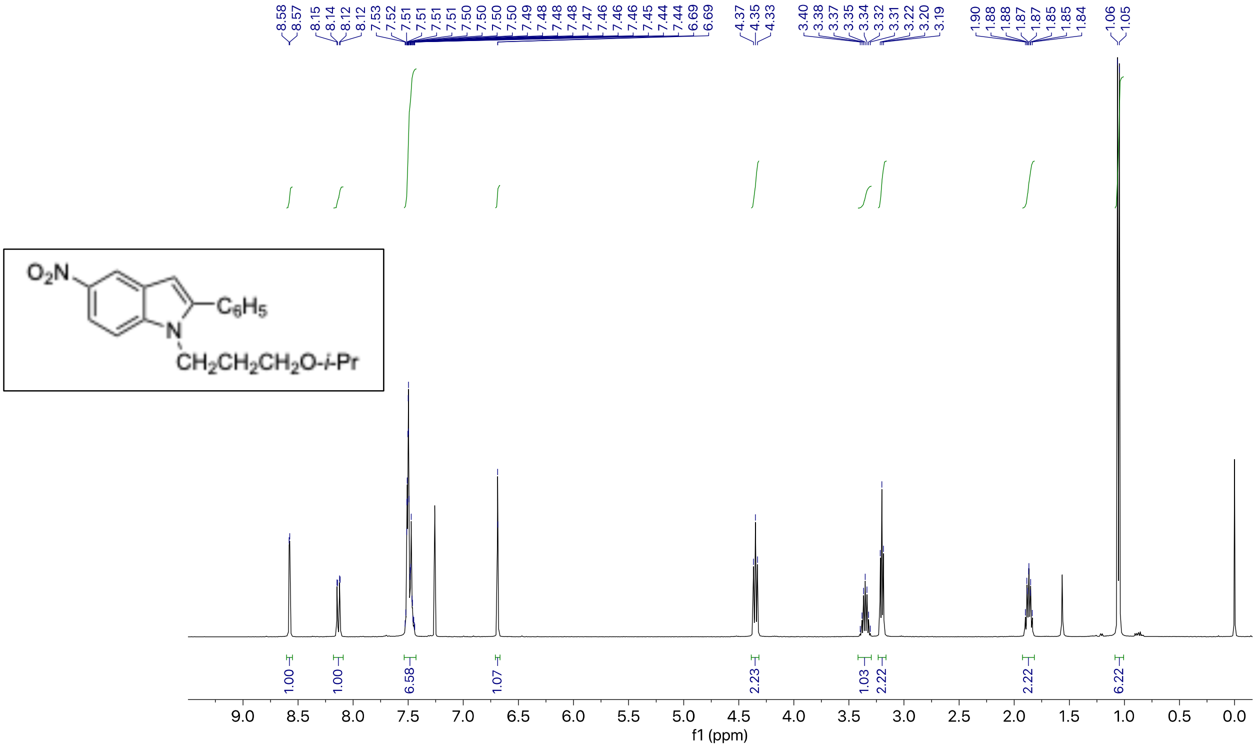

^{13}C NMR of 1-(3-isopropoxypropyl)-5-nitro-2-phenyl-1*H*-indole (**16**)

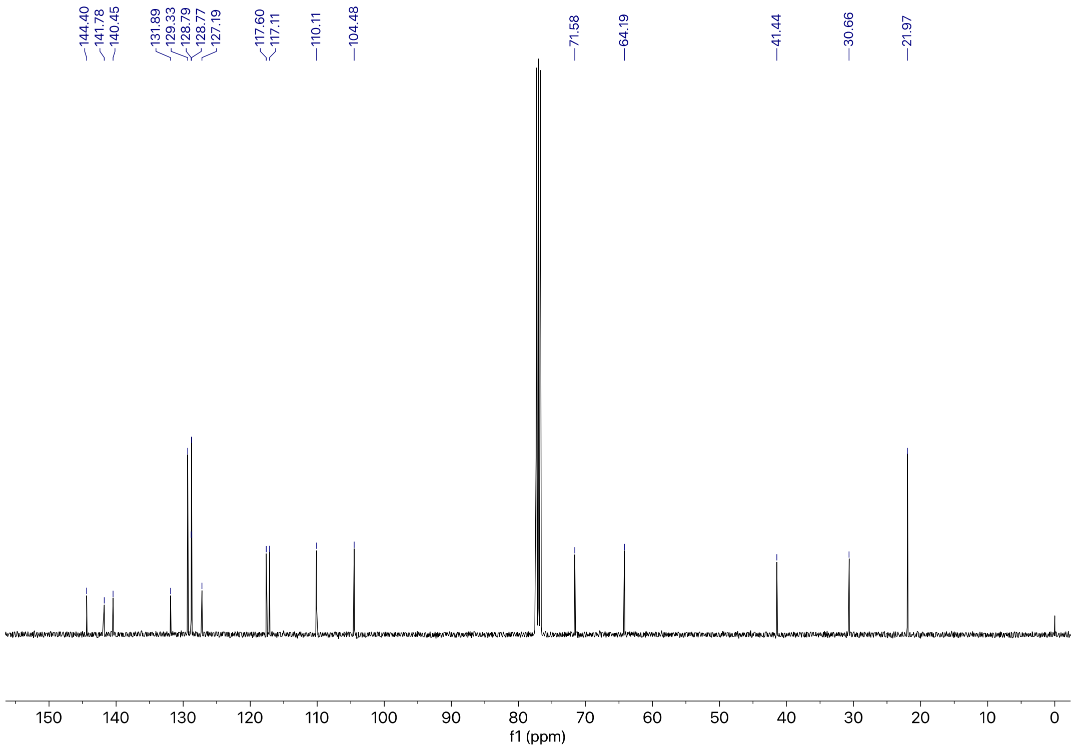

^{1}H NMR of 1-cyclohexyl-5-nitro-2-phenyl-1*H*-indole (**17**)

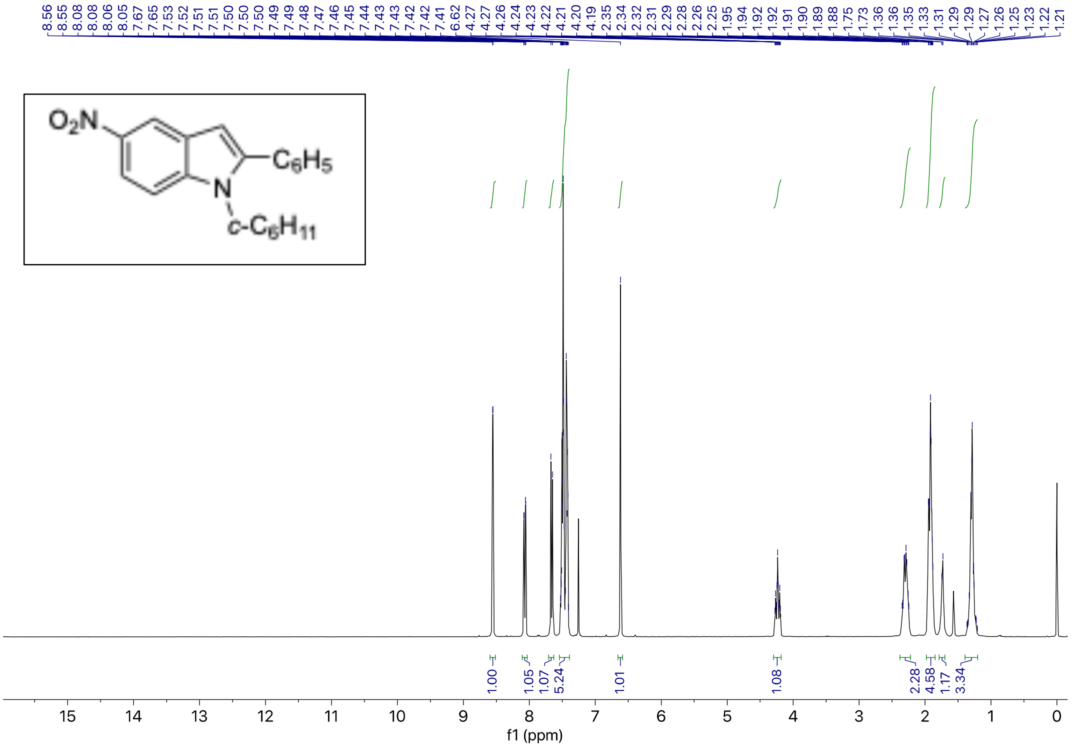

^{13}C NMR of 1-cyclohexyl-5-nitro-2-phenyl-1*H*-indole (**17**)

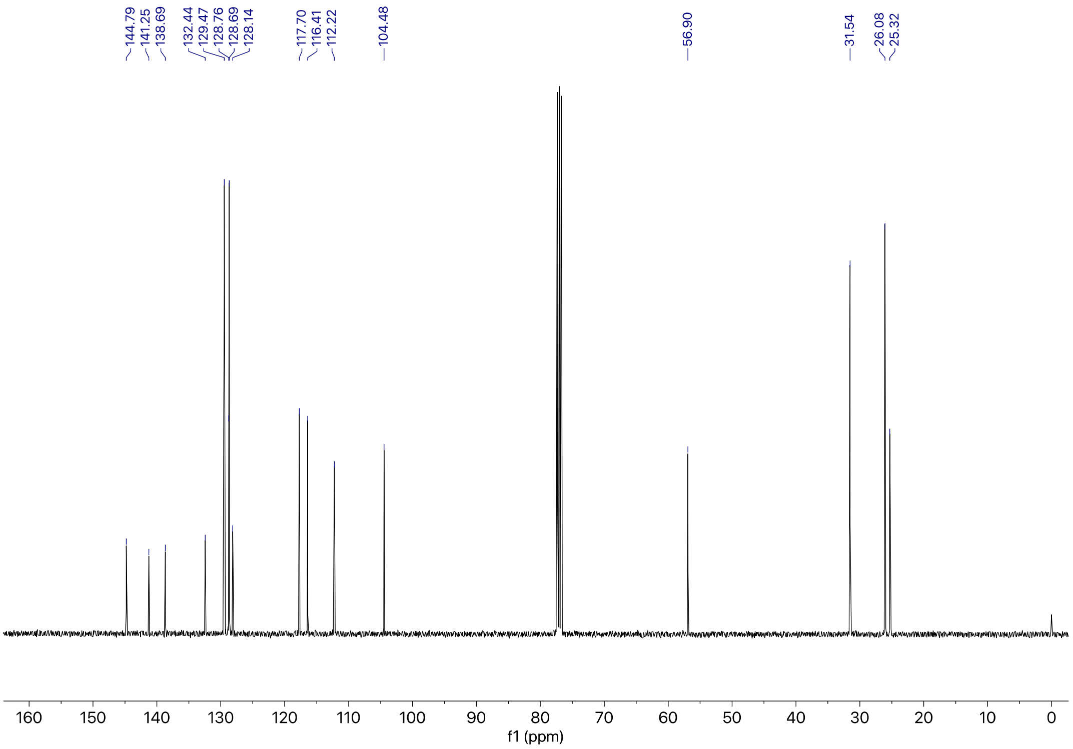

^{1}H NMR of 1-(4-methoxyphenyl)-5-nitro-2-phenyl-1*H*-indole (**18**)

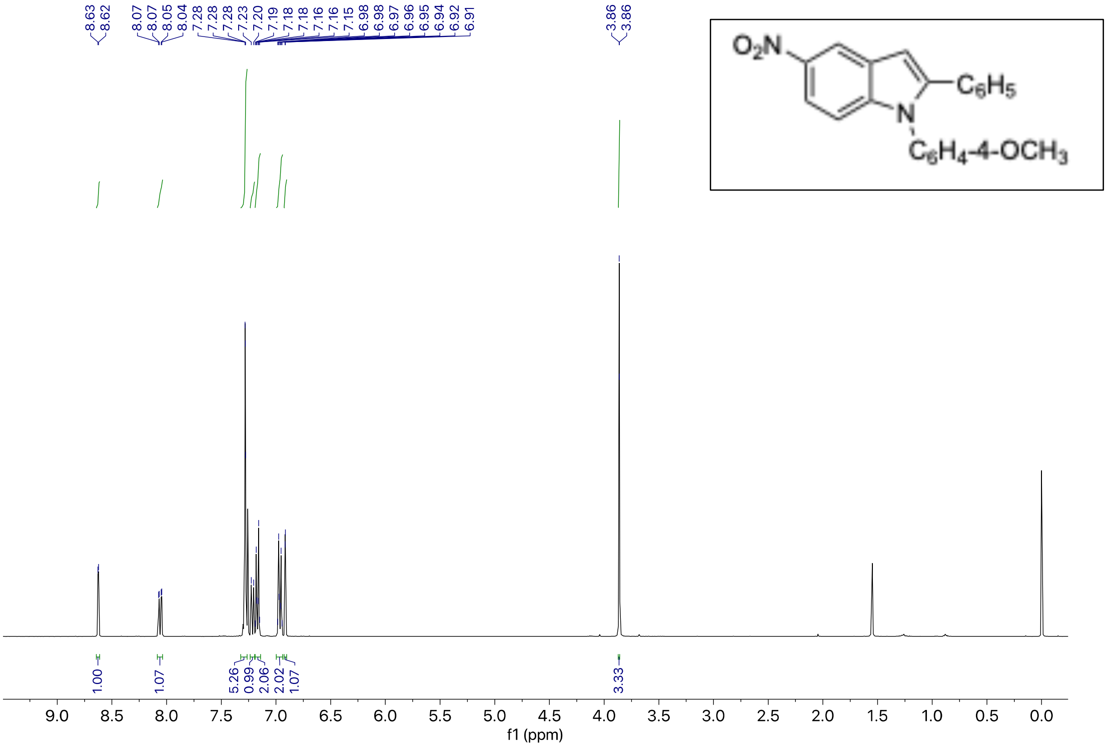

^{13}C NMR of 1-(4-methoxyphenyl)-5-nitro-2-phenyl-1*H*-indole (**18**)

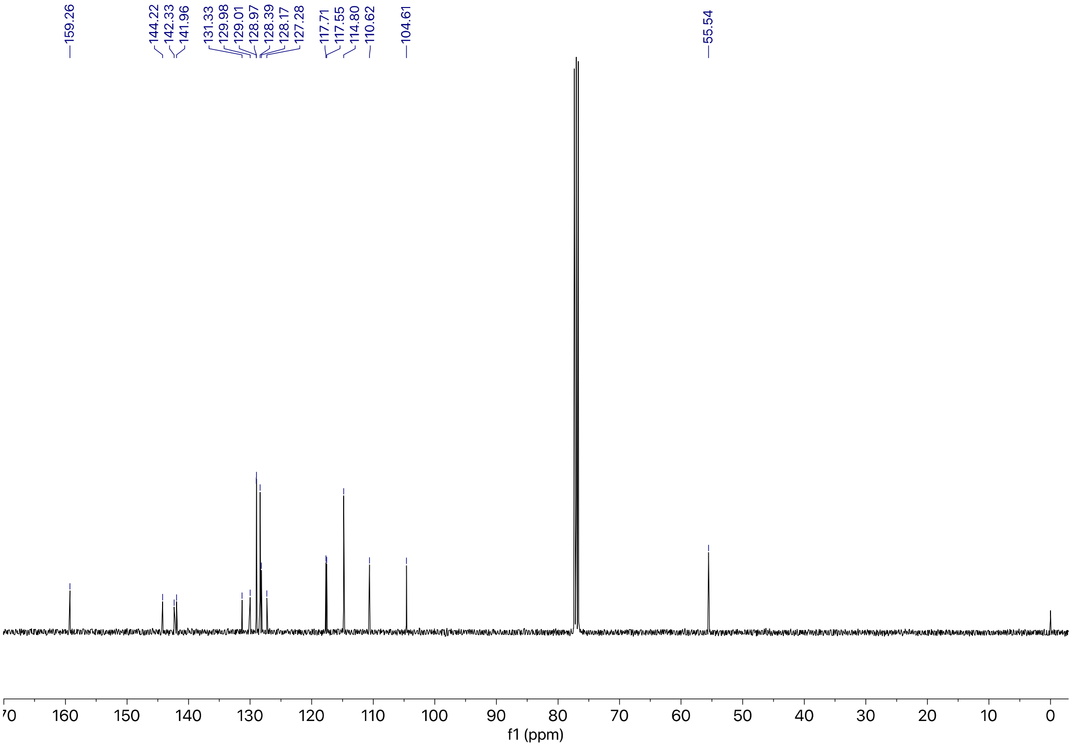

^{1}H NMR of 5-nitro-1,2-diphenyl-1*H*-indole (**19**)

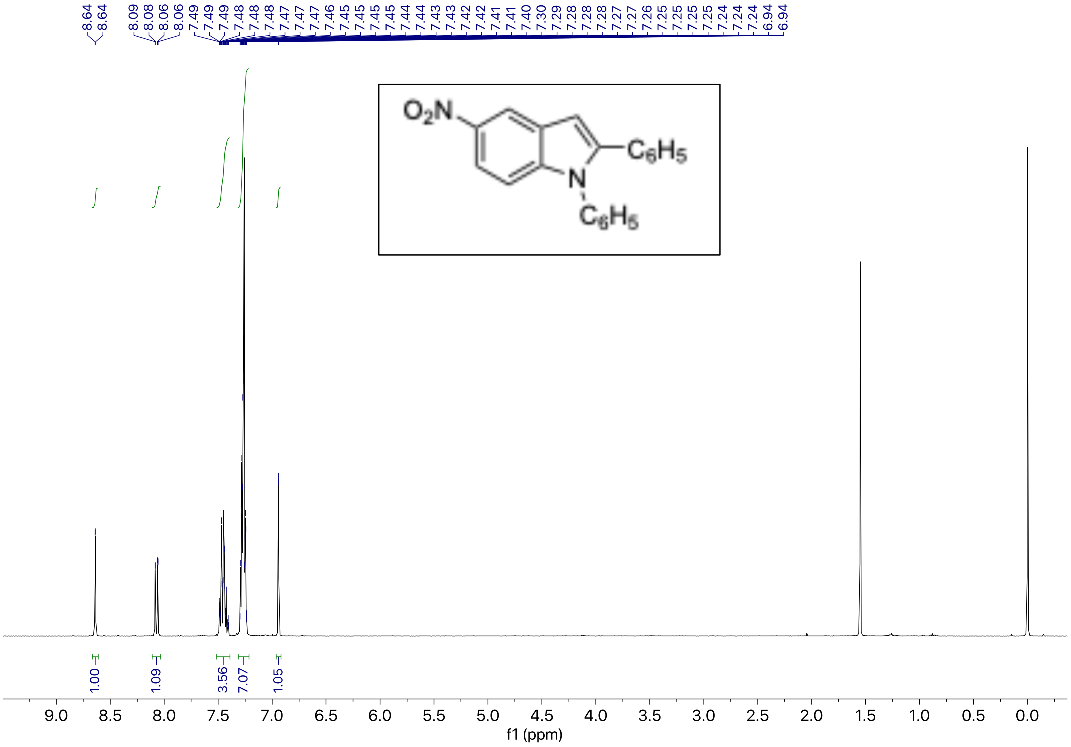

^{13}C NMR of 5-nitro-1,2-diphenyl-1*H*-indole (**19**)

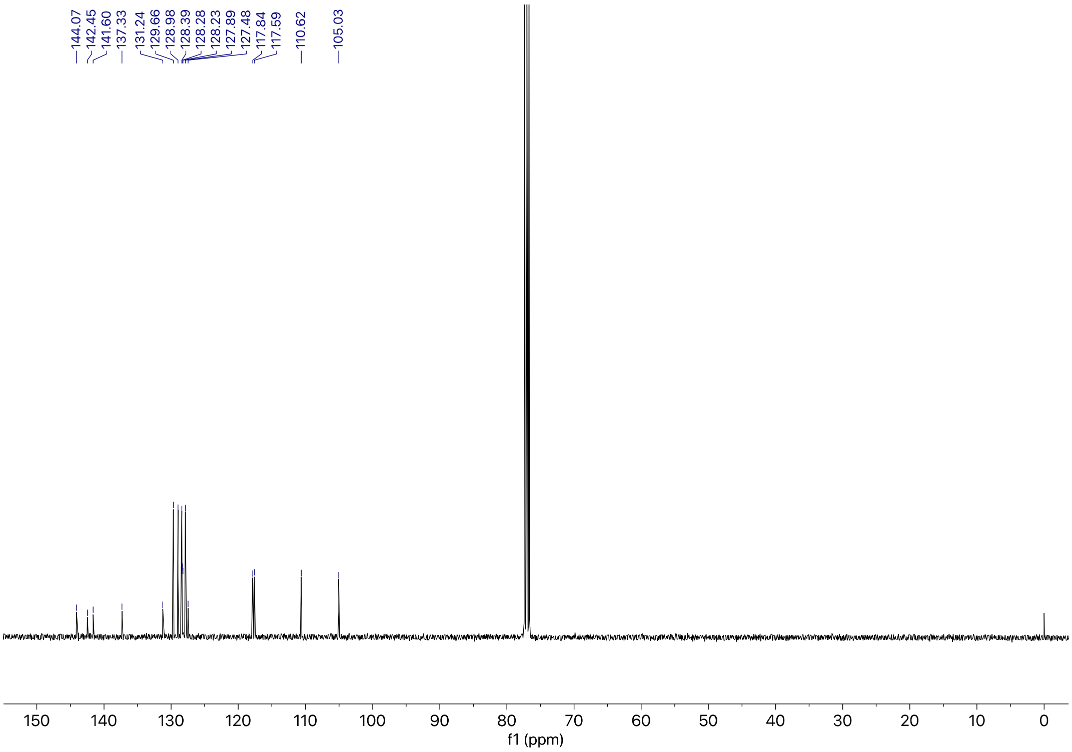

^{1}H NMR of 1-(4-fluorophenyl)-5-nitro-2-phenyl-1*H*-indole (**20**)

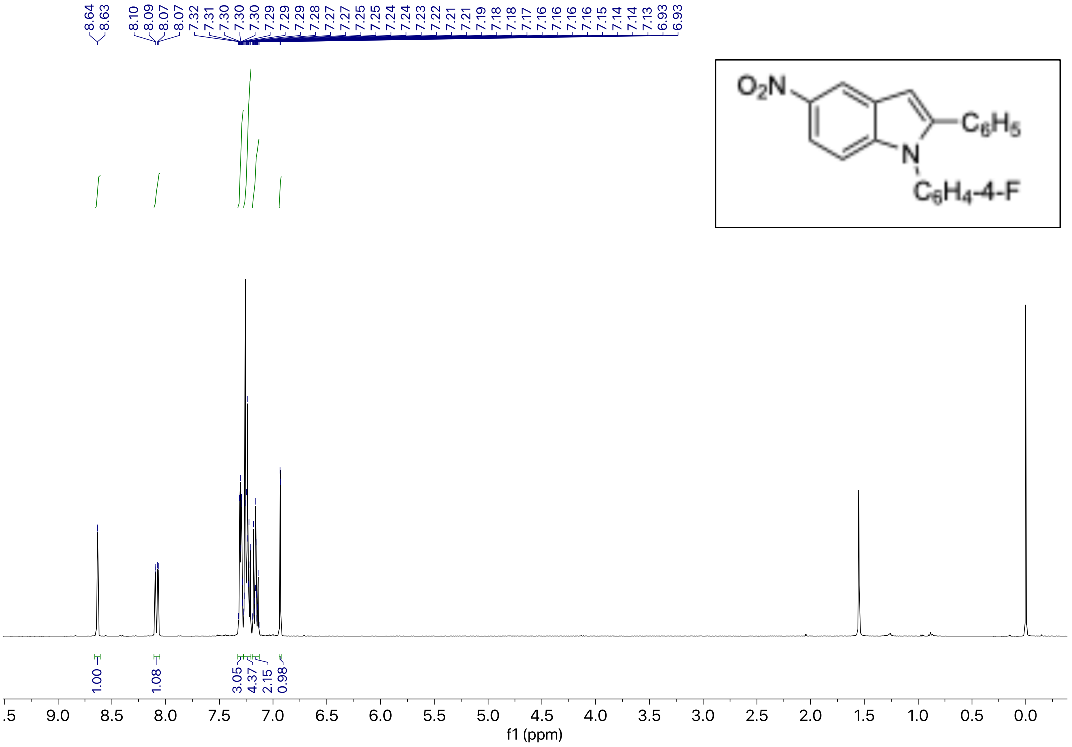

^{13}C NMR of 1-(4-fluorophenyl)-5-nitro-2-phenyl-1*H*-indole (**20**)

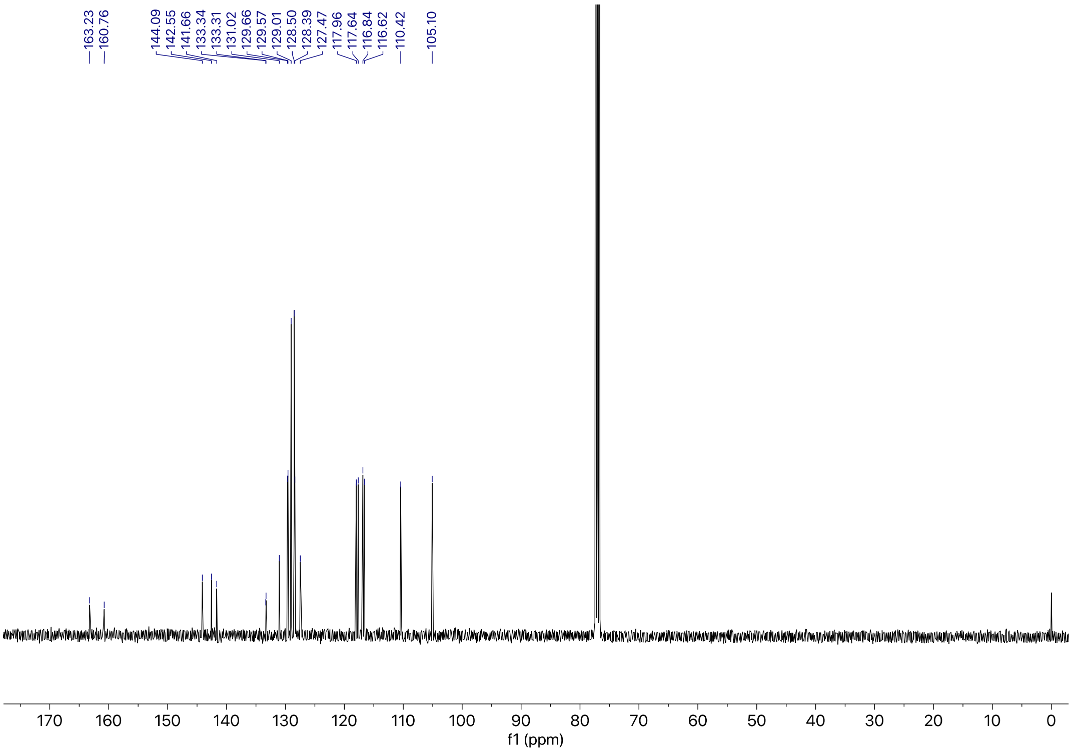

^{19}F NMR of 1-(4-fluorophenyl)-5-nitro-2-phenyl-1*H*-indole (**20**)

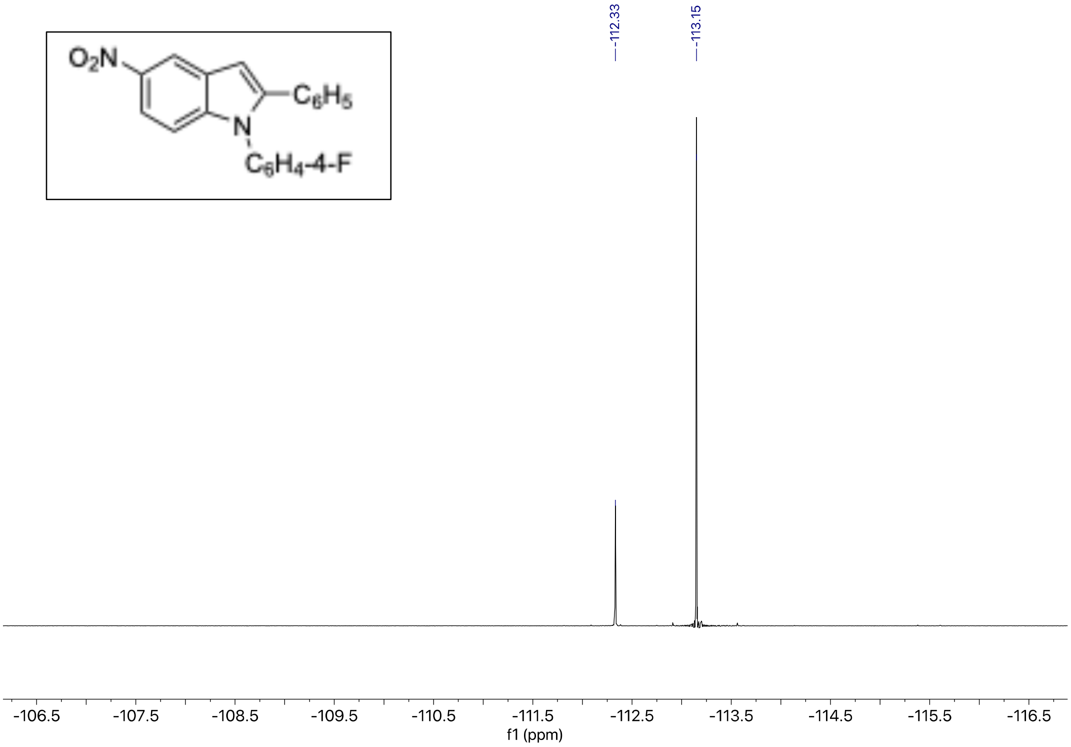

^{1}H NMR of 5-nitro-2-phenyl-1*H*-indole (**20a**)

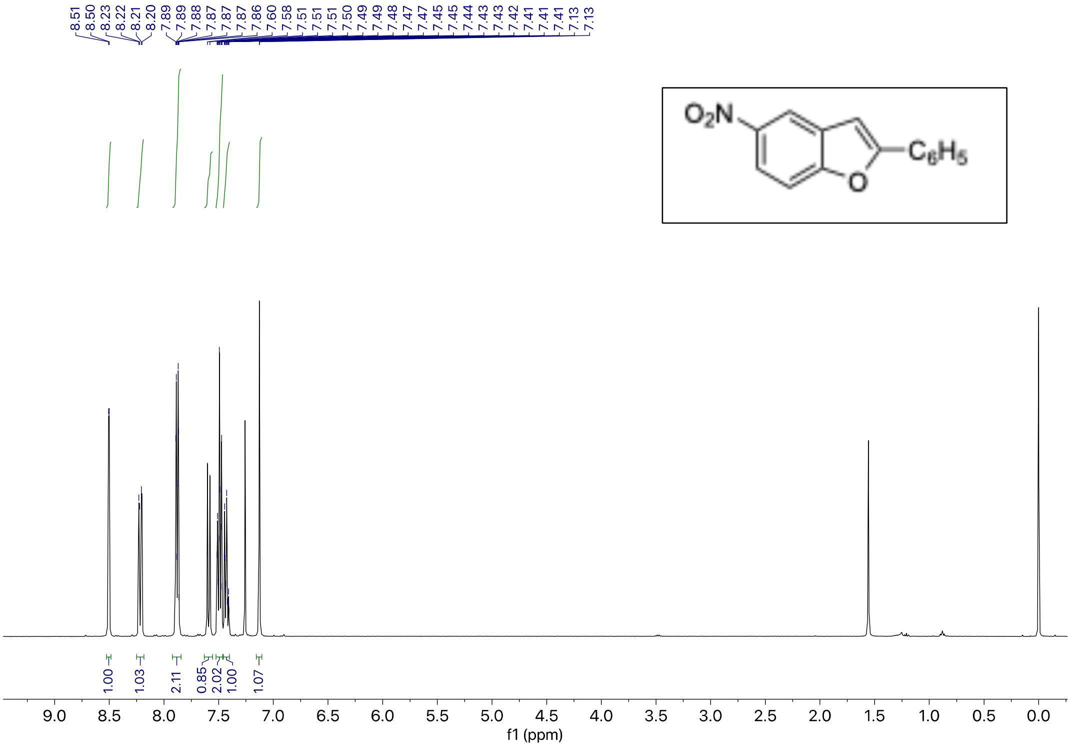

^{13}C NMR of 5-nitro-2-phenyl-1*H*-indole (**20a**)

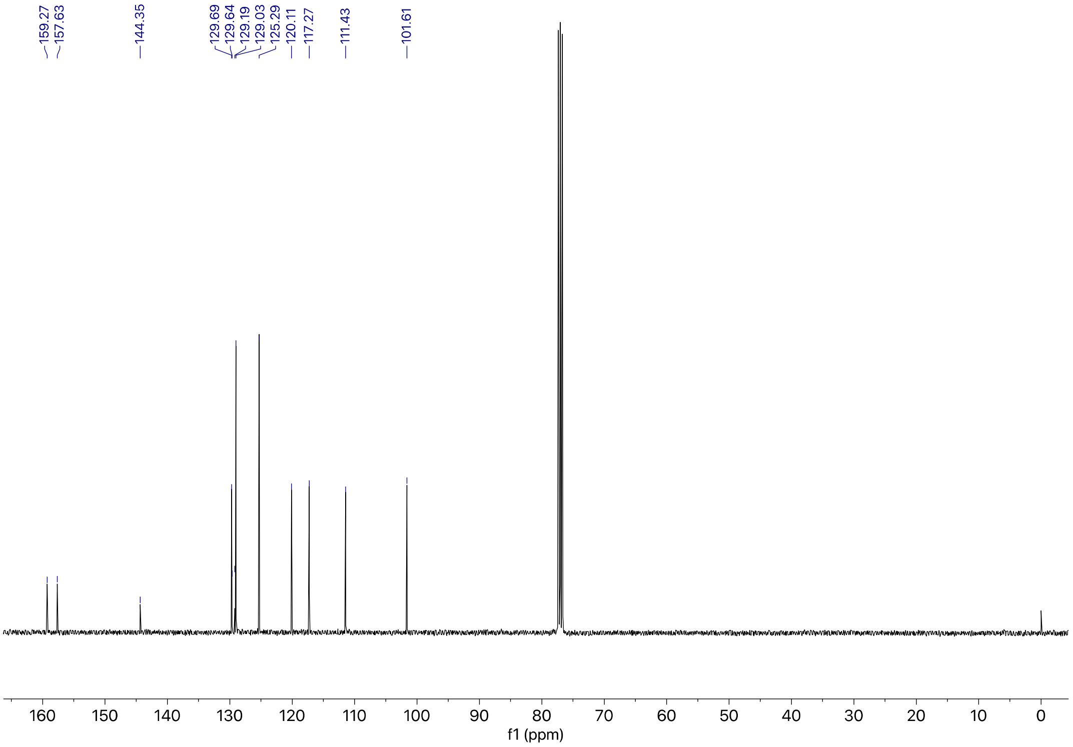

^{1}H NMR of 1-benzyl-2-(4-methylphenyl)-5-nitro-1*H*-indole (**22**)

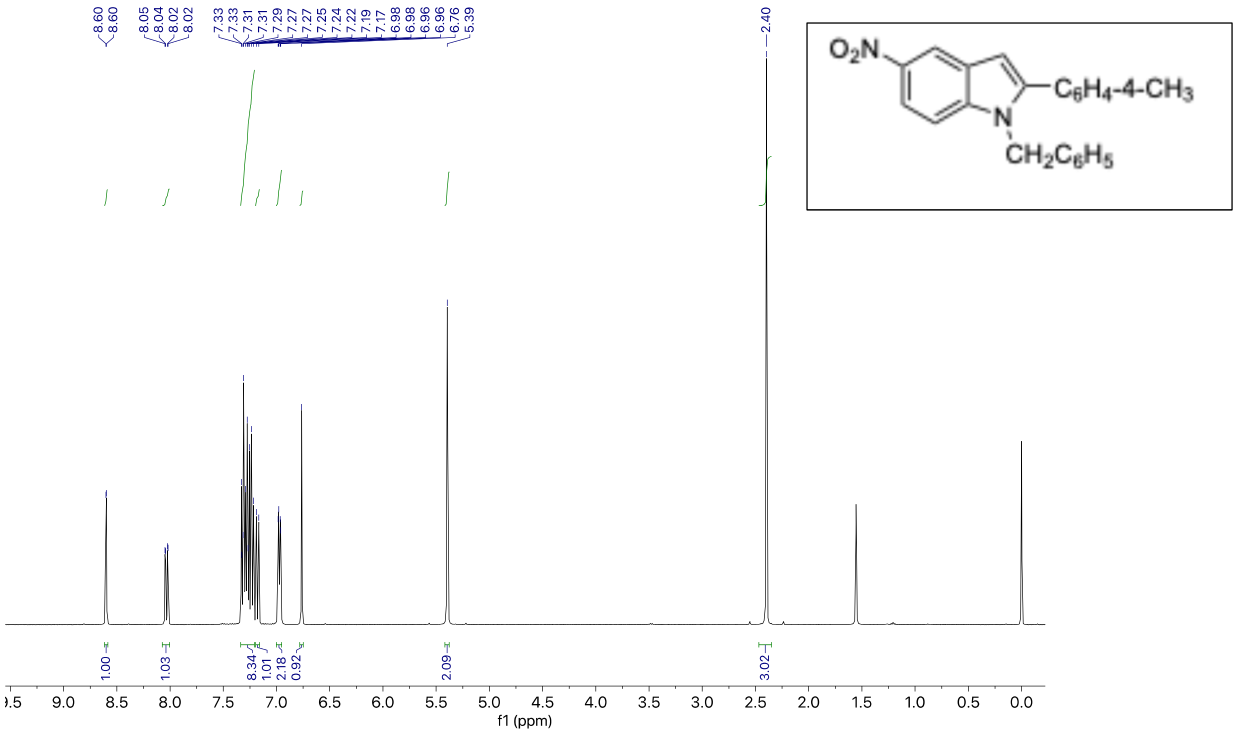

¹³C NMR of 1-benzyl-2-(4-methylphenyl)-5-nitro-1*H*-indole (**22**)

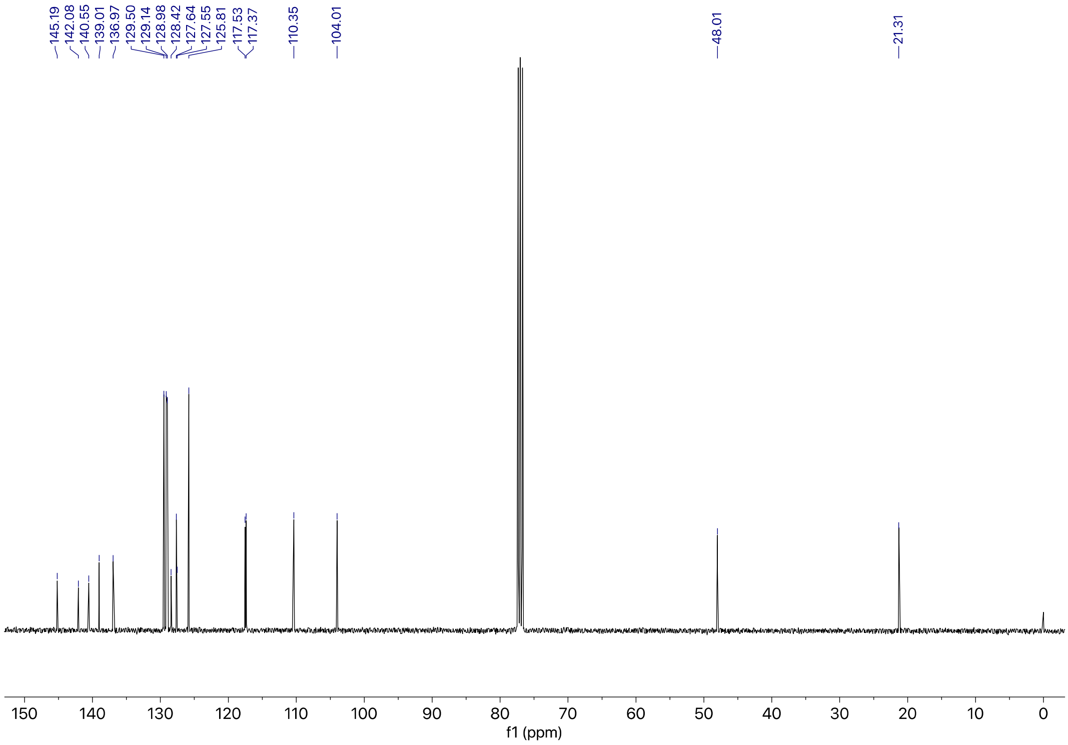

¹H NMR of 1-(3-methoxybenzyl)-2-(4-methylphenyl)-5-nitro-1*H*-indole (**23**)

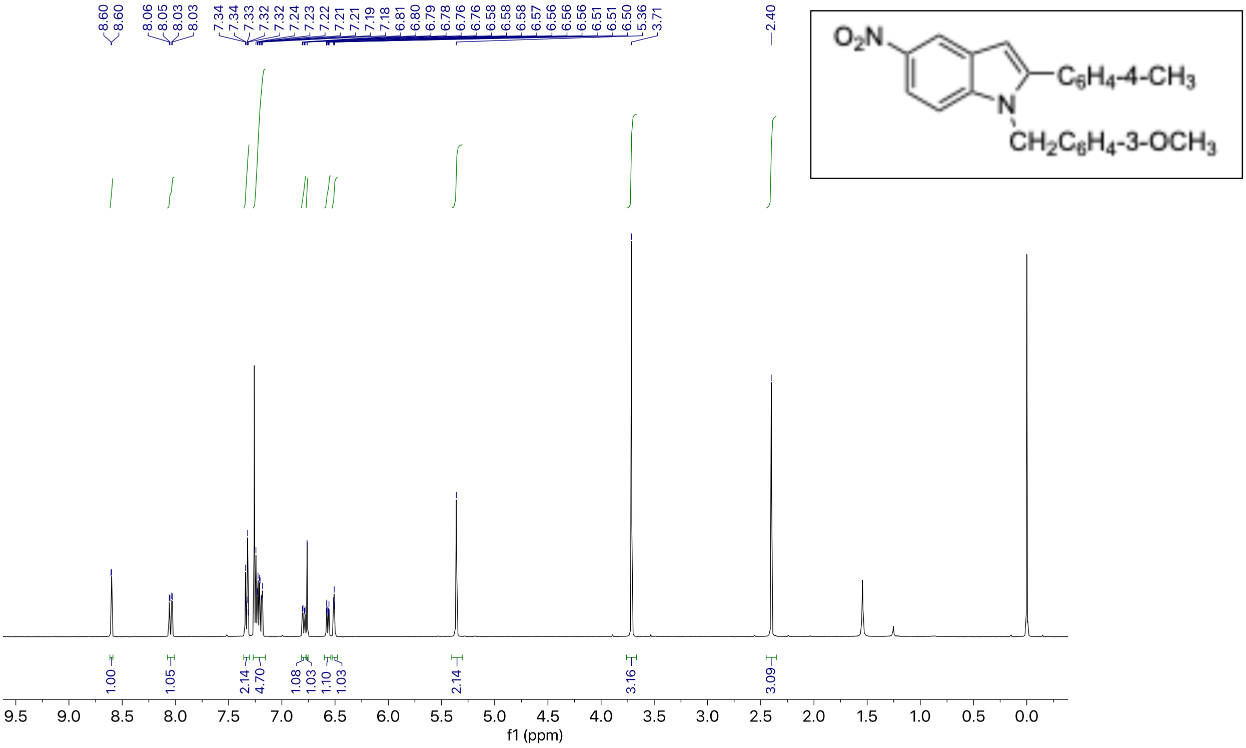

^{13}C NMR of 1-(3-methoxybenzyl)-2-(4-methylphenyl)-5-nitro-1*H*-indole (**23**)

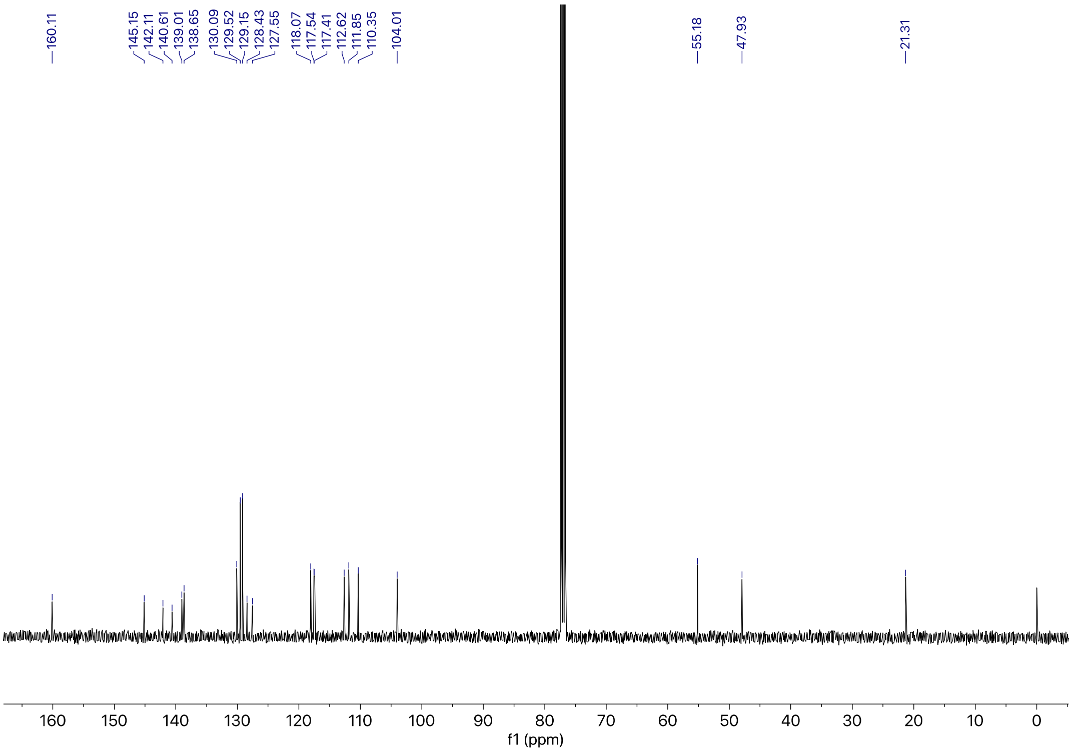

^{1}H NMR of 2-(4-methylphenyl)-5-nitro-1-phenethyl-1*H*-indole (**24**)

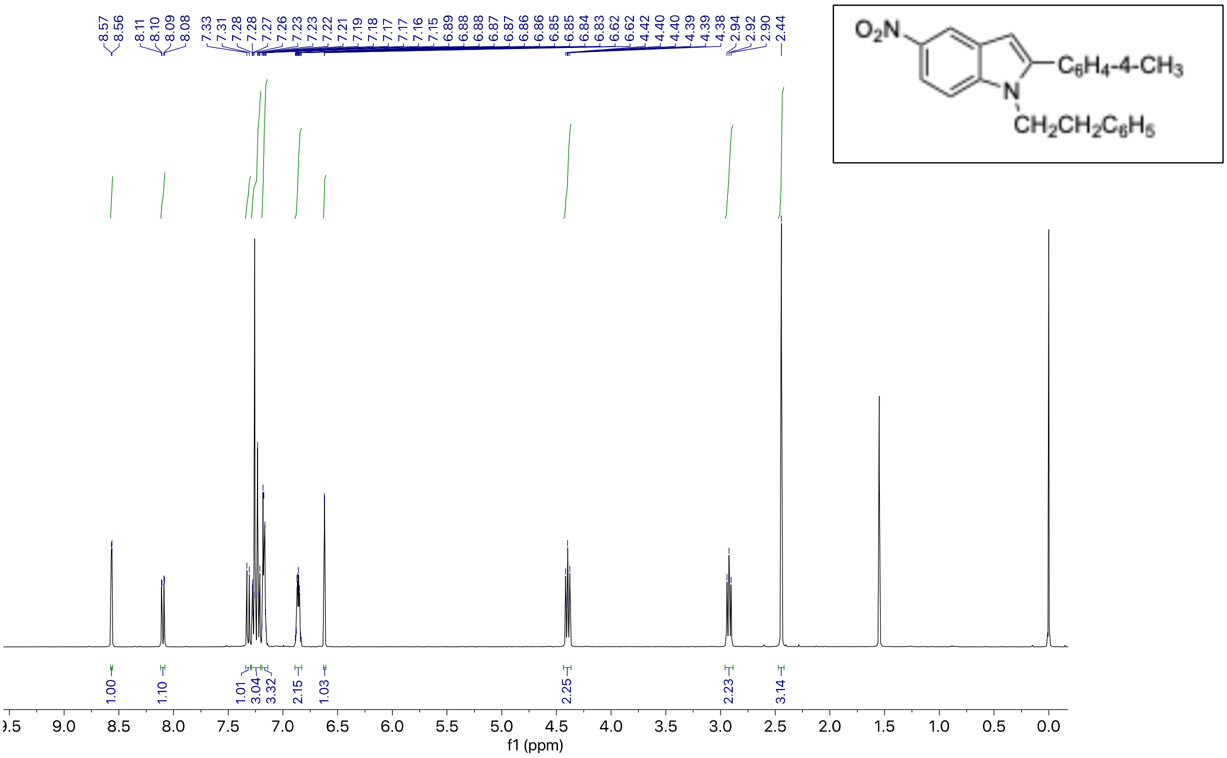

^{13}C NMR of 2-(4-methylphenyl)-5-nitro-1-phenethyl-1*H*-indole (**24**)

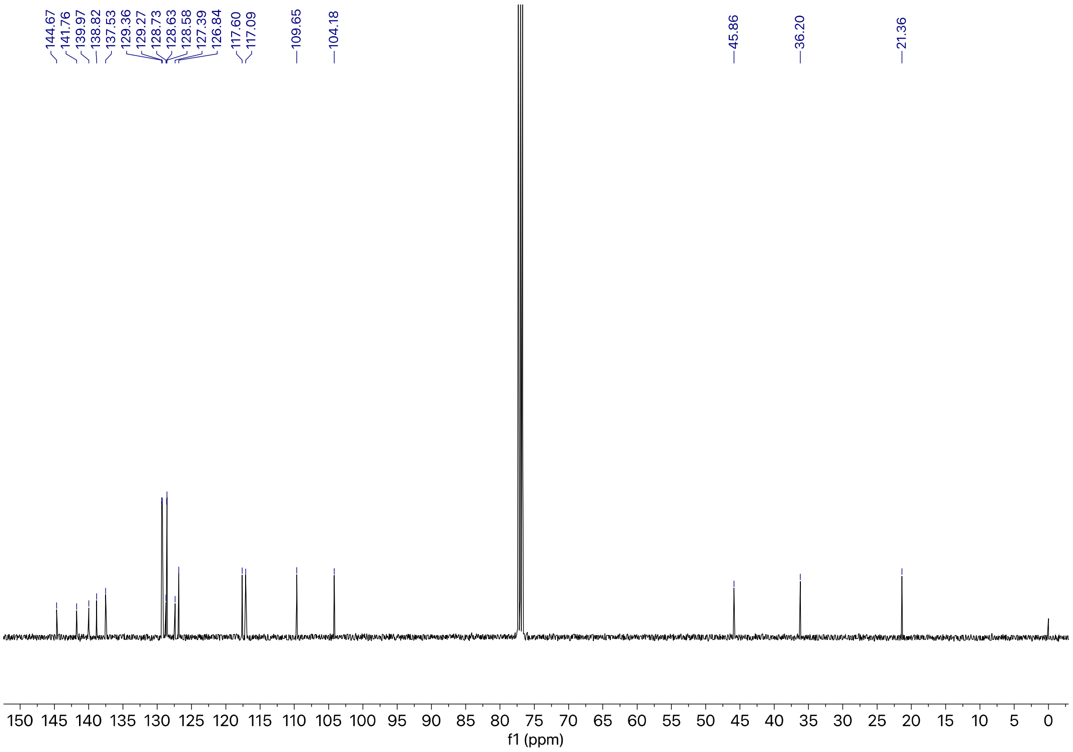

^{1}H NMR of 1-benzyl-2-(4-methoxyphenyl)-5-nitro-1*H*-indole (**25**)

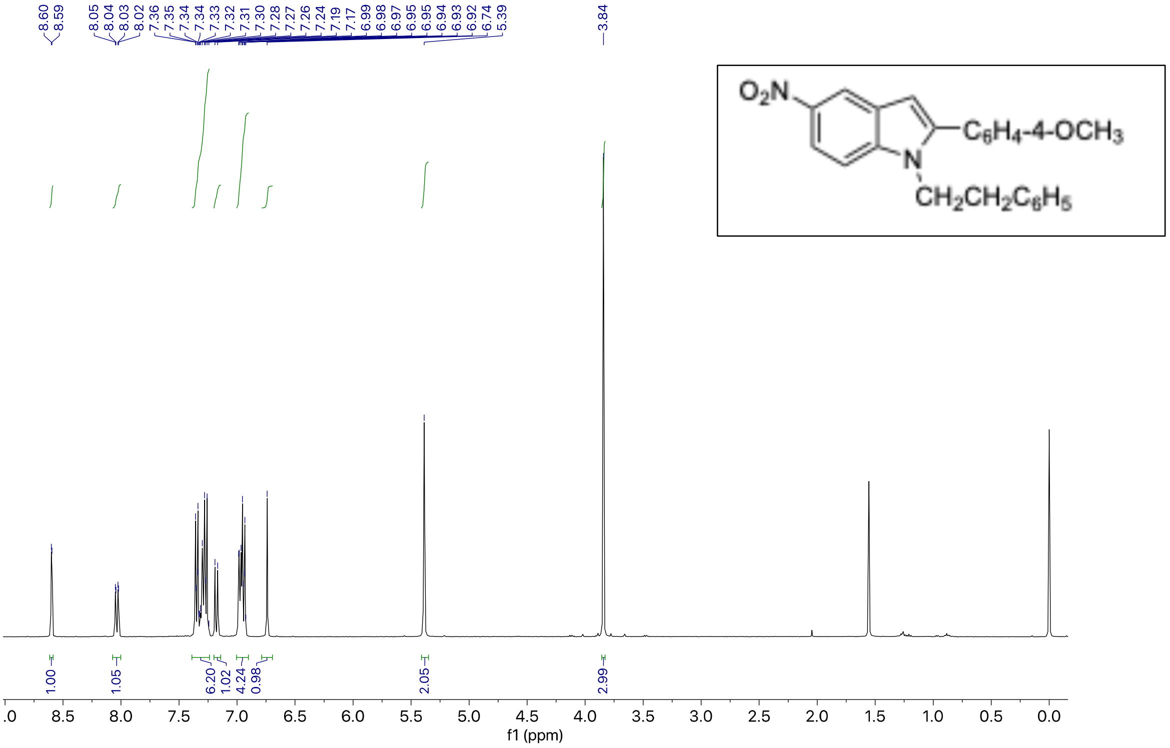

^{13}C NMR of 1-benzyl-2-(4-methoxyphenyl)-5-nitro-1*H*-indole (**25**)

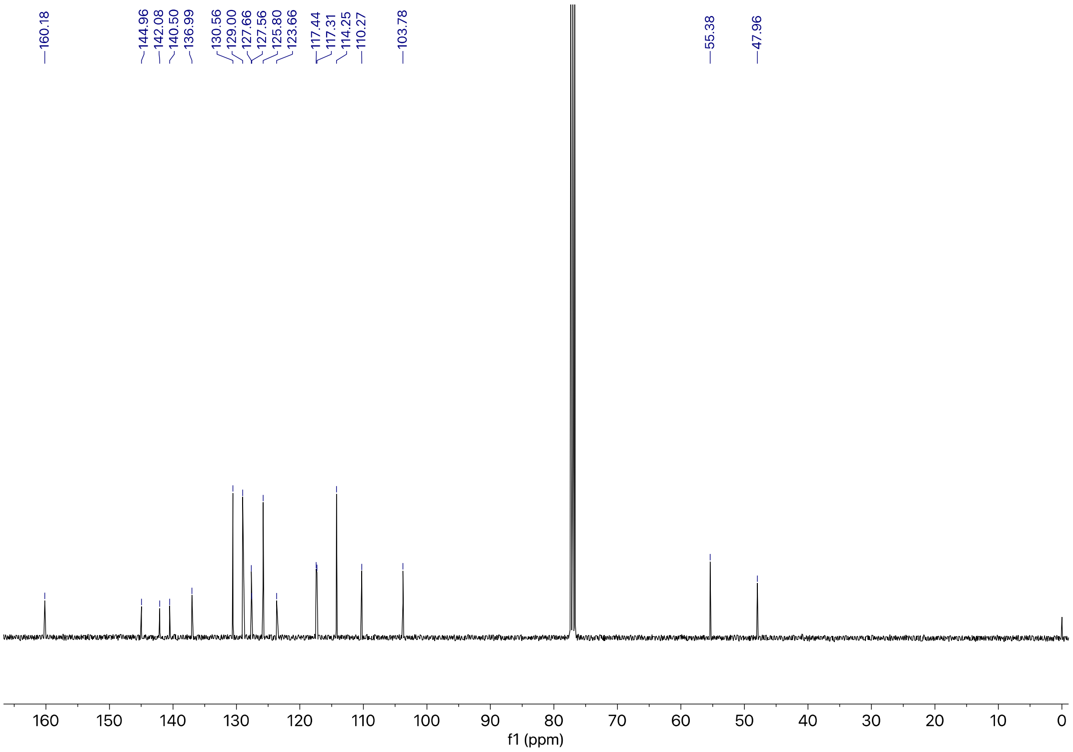

^{1}H NMR of 1-(3-methoxybenzyl)-2-(4-methoxyphenyl)-5-nitro-1*H*-indole (**26**)

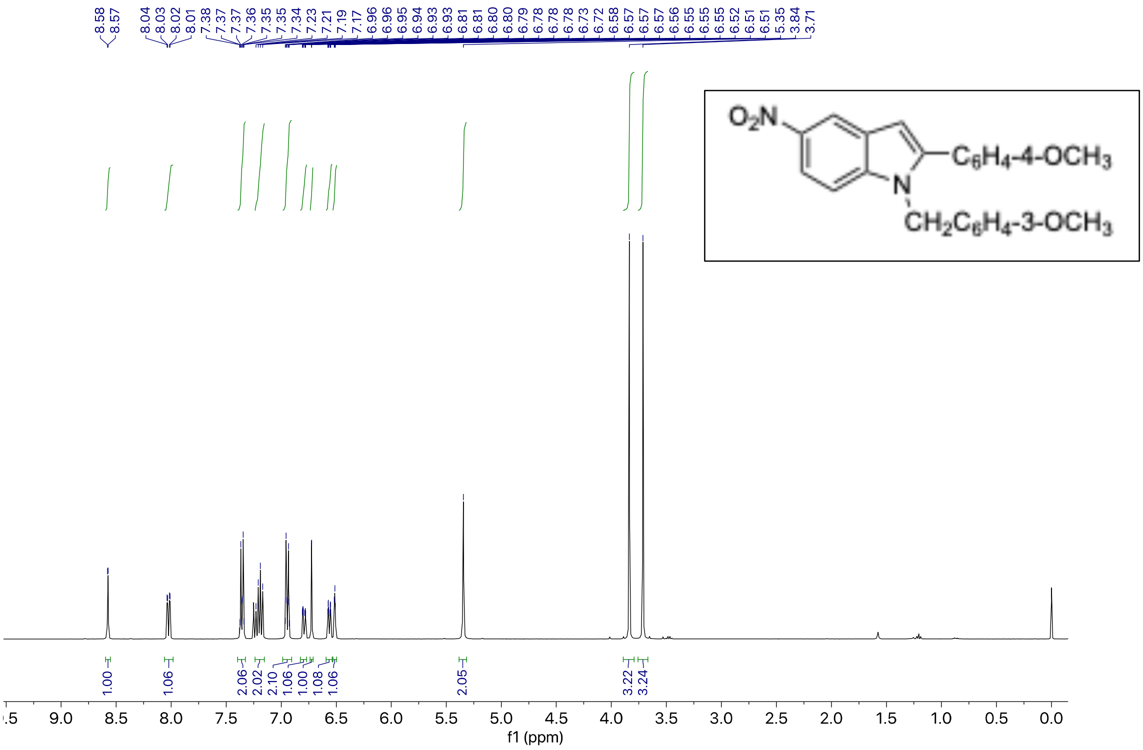

^{13}C NMR of 1-(3-methoxybenzyl)-2-(4-methoxyphenyl)-5-nitro-1*H*-indole (**26**)

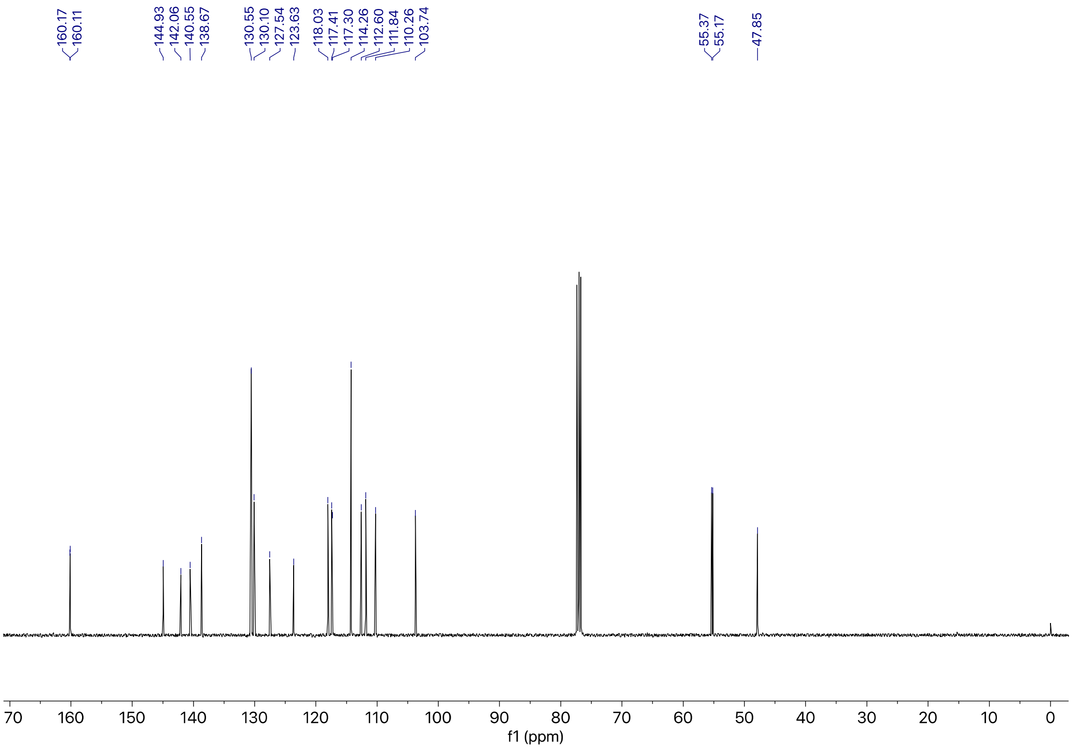

^{1}H NMR of 1-(2-chlorophenyl)-2-(4-methoxyphenyl)-5-nitro-1*H*-indole (**27**)

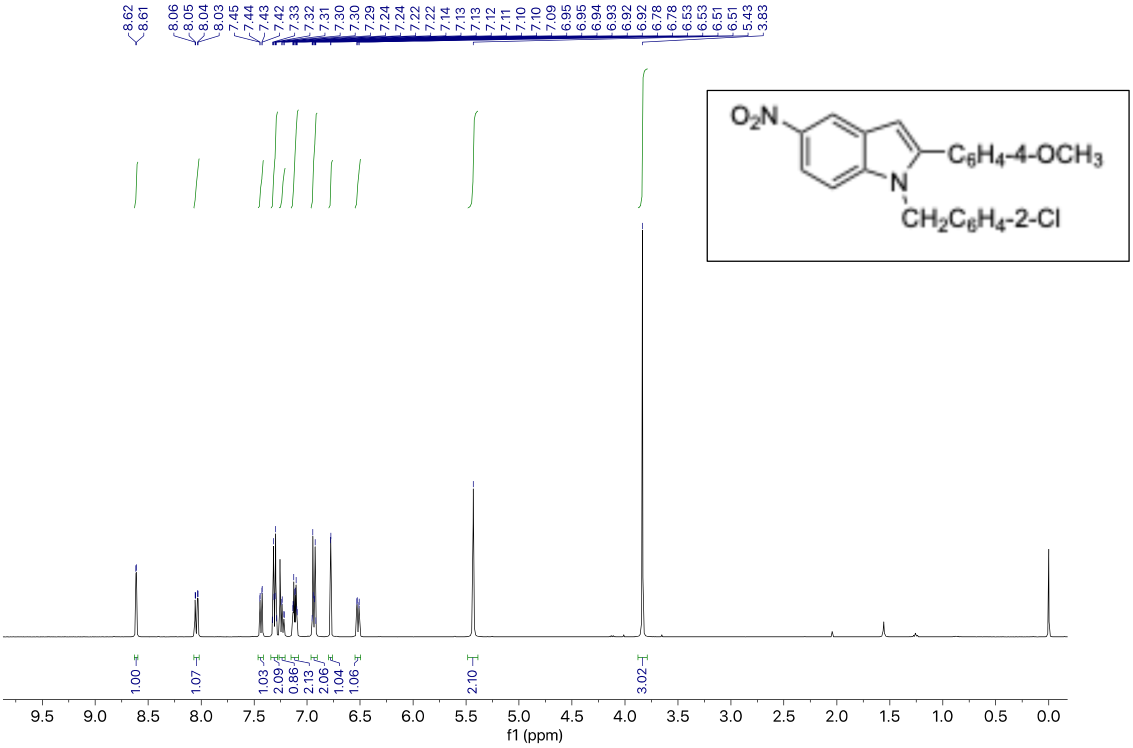

^{13}C NMR of 1-(2-chlorophenyl)-2-(4-methoxyphenyl)-5-nitro-1*H*-indole (**27**)

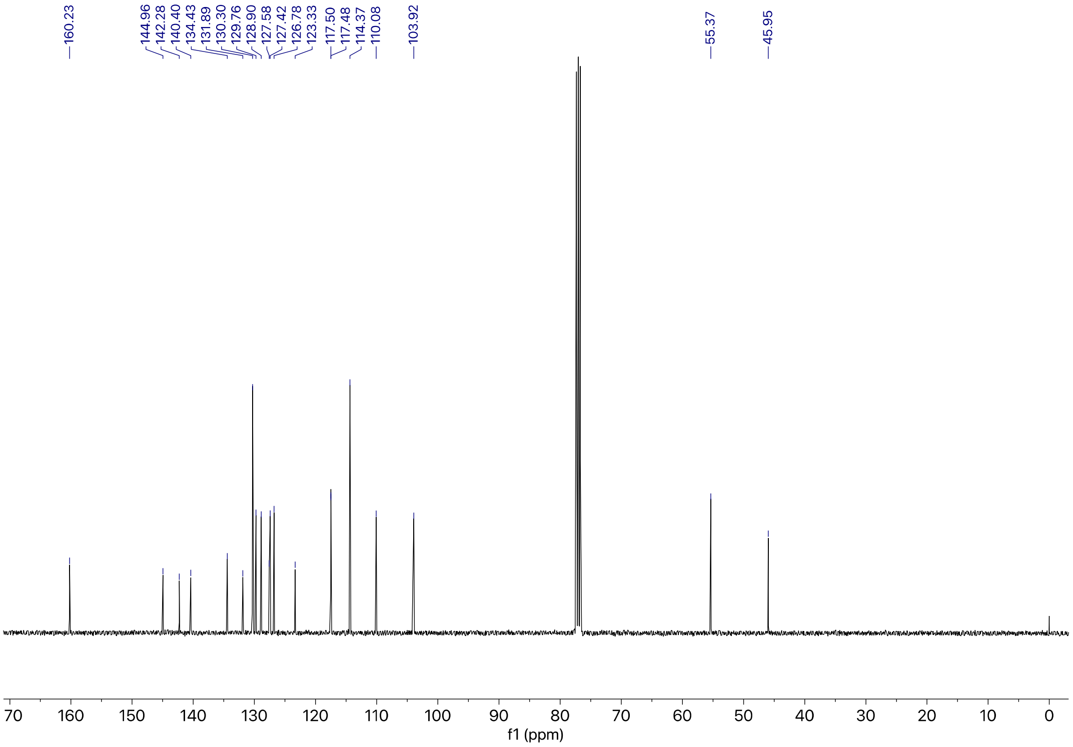

^{1}H NMR of 2-(4-methoxyphenyl)-5-nitro-1-(3-(trifluoromethyl)benzyl)-1*H*-indole (**28**)

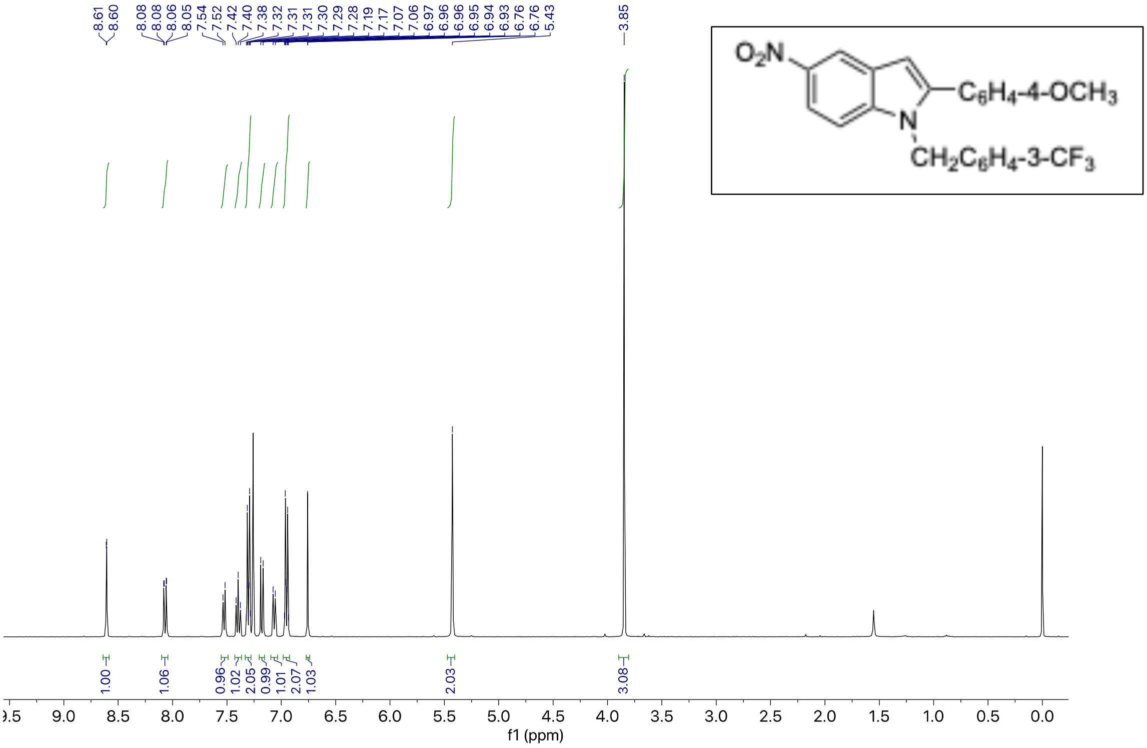

^{13}C NMR of 2-(4-methoxyphenyl)-5-nitro-1-(3-(trifluoromethyl)benzyl)-1*H*-indole (**28**)

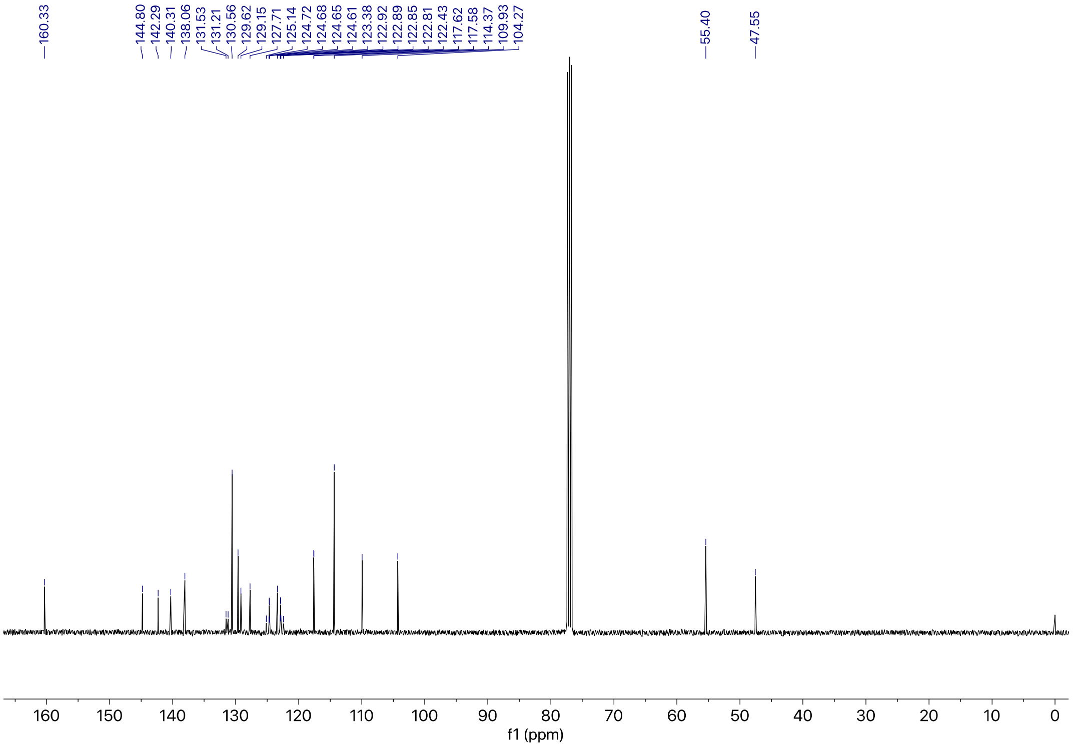

^{19}F NMR of 2-(4-methoxyphenyl)-5-nitro-1-(3-(trifluoromethyl)benzyl)-1*H*-indole (**28**)

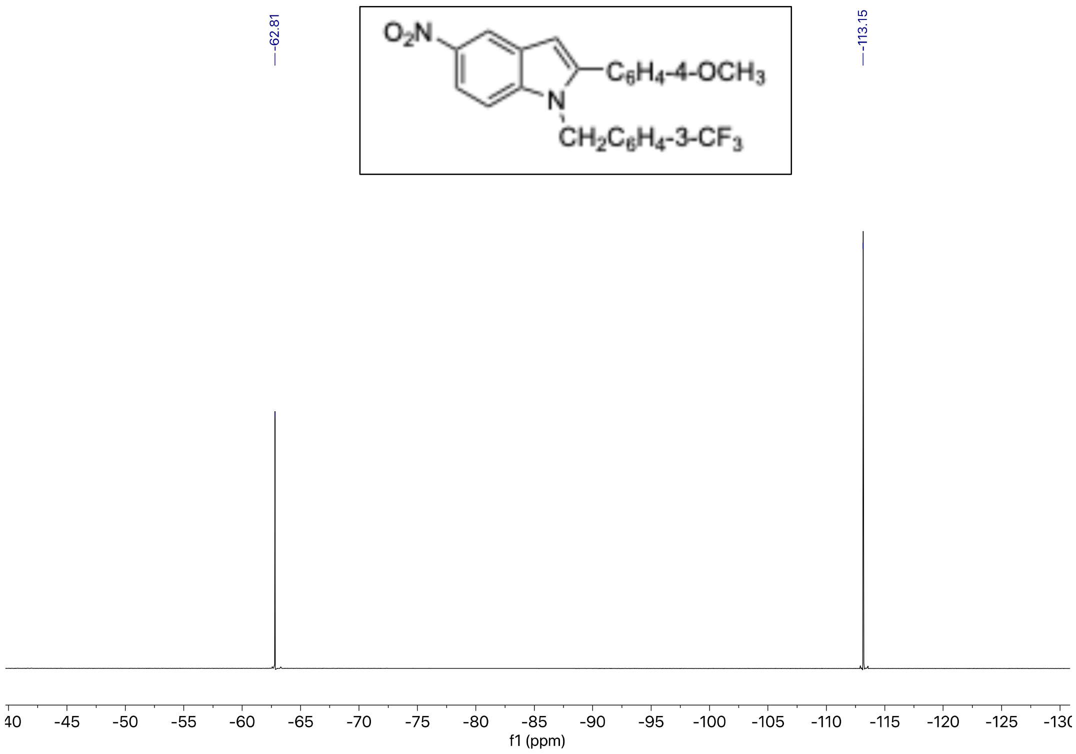

^{1}H NMR of 2-(4-methoxyphenyl)-5-nitro-1-phenethyl-1*H*-indole (**29**)

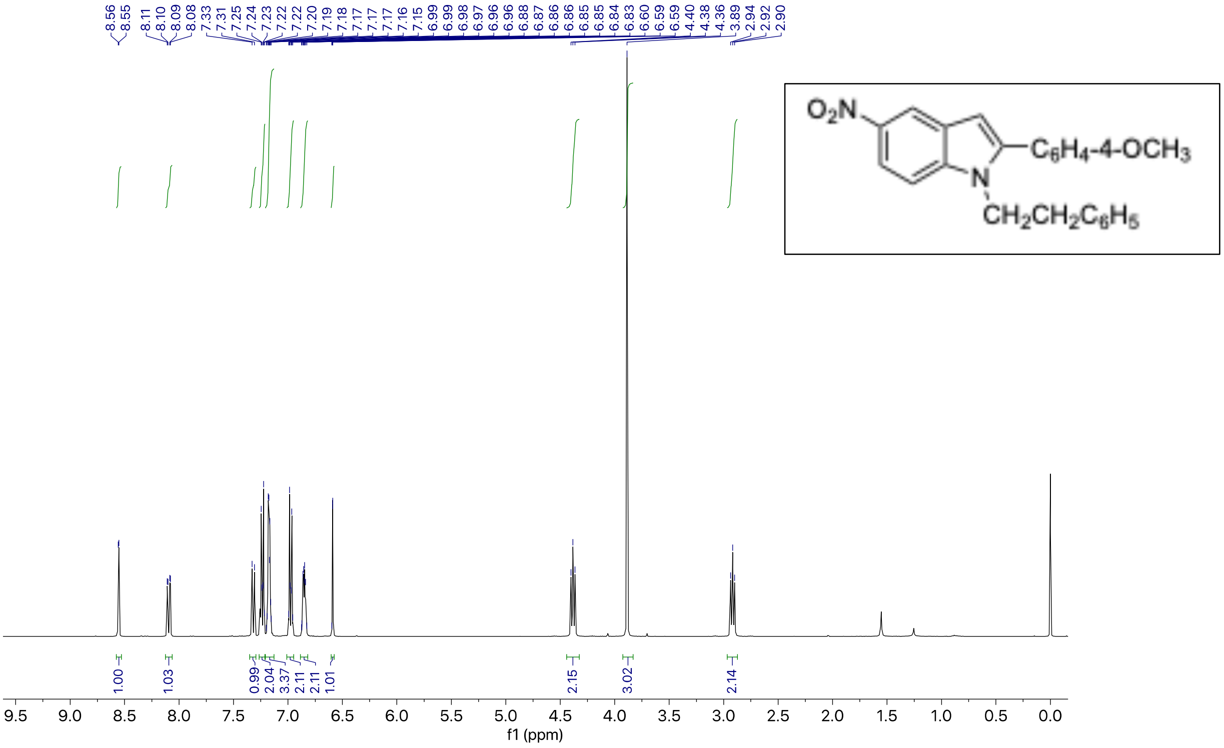

^{13}C NMR of 2-(4-methoxyphenyl)-5-nitro-1-phenethyl-1*H*-indole (**29**)

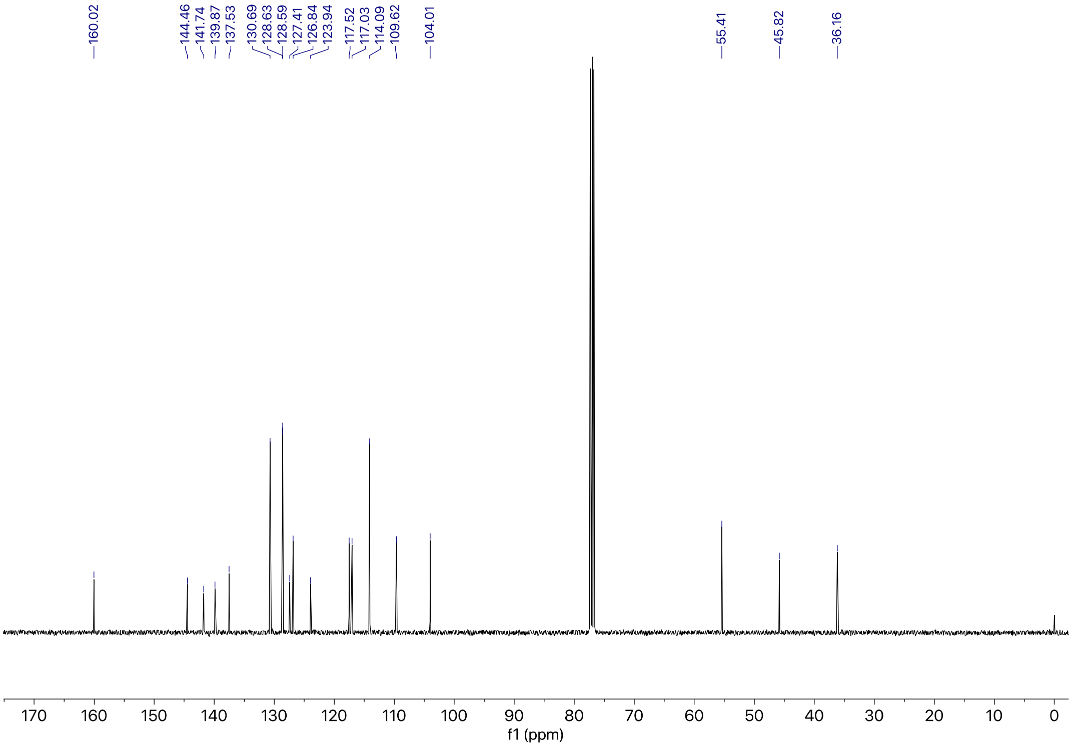

^{1}H NMR of ^{13}C NMR of 1-hexyl-2-(4-methoxyphenyl)-5-nitro-1*H*-indole (**30**)

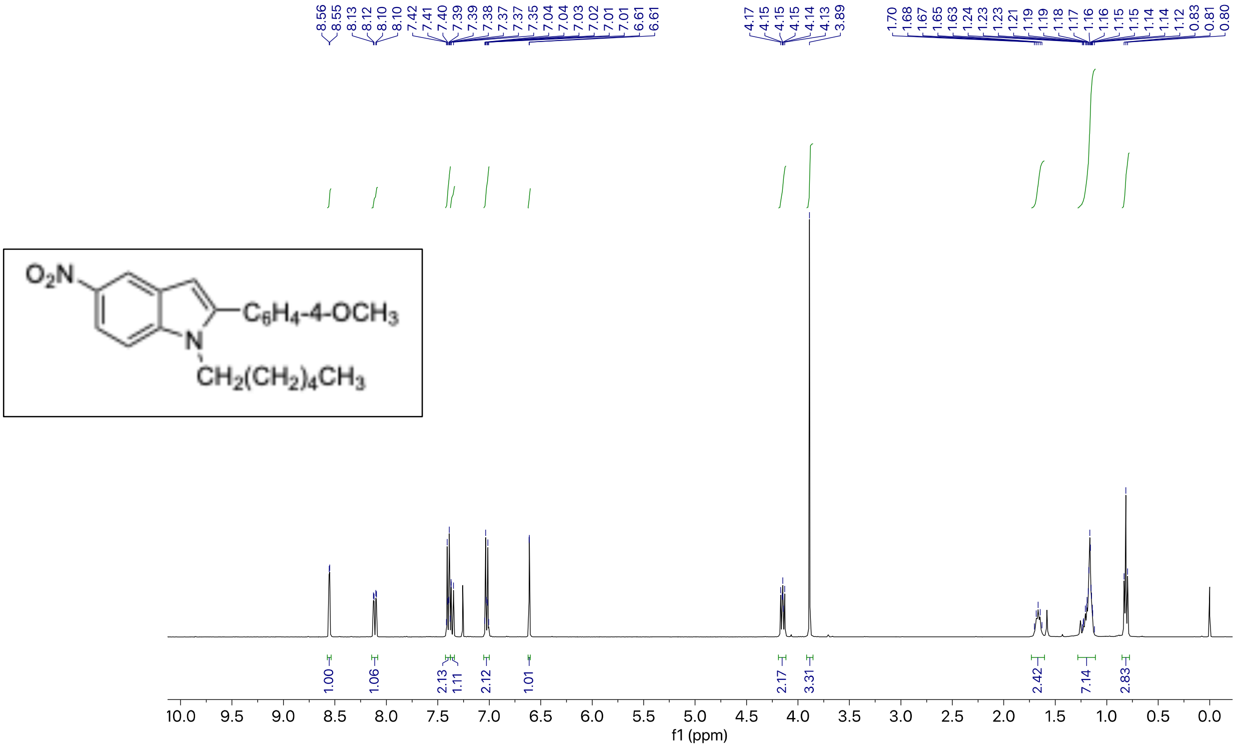

^{13}C NMR of 1-hexyl-2-(4-methoxyphenyl)-5-nitro-1*H*-indole (**30**)

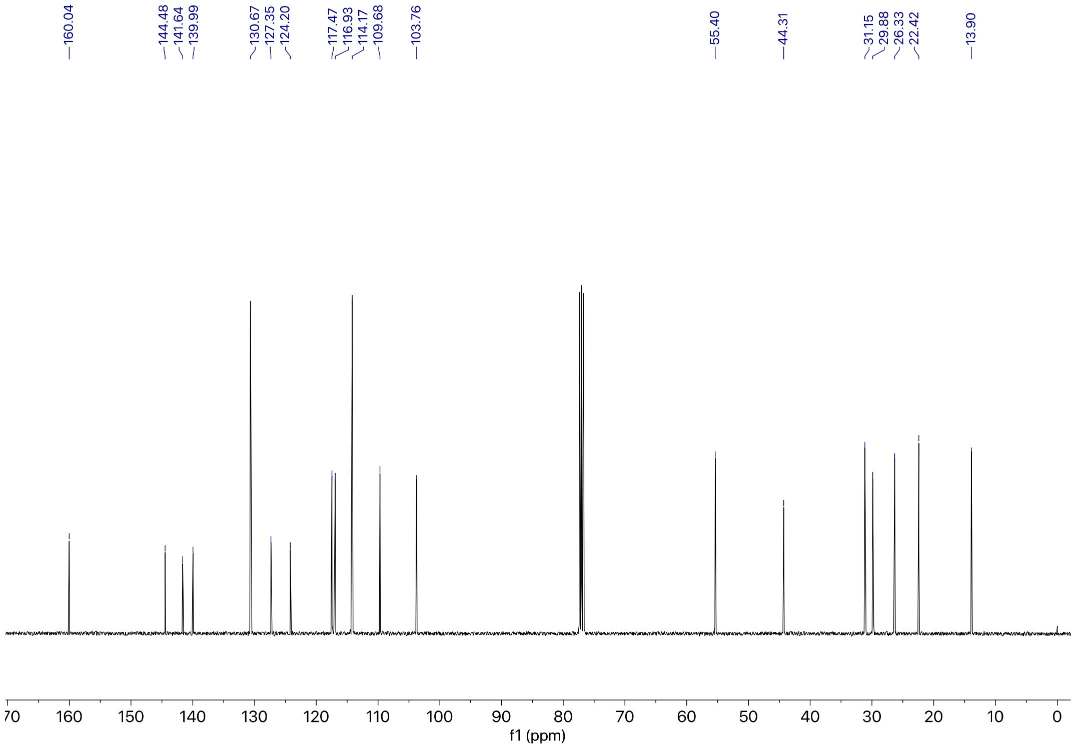

^{1}H NMR of 1-benzyl-2-(4-fluorophenyl)-5-nitro-1*H*-indole (**31**)

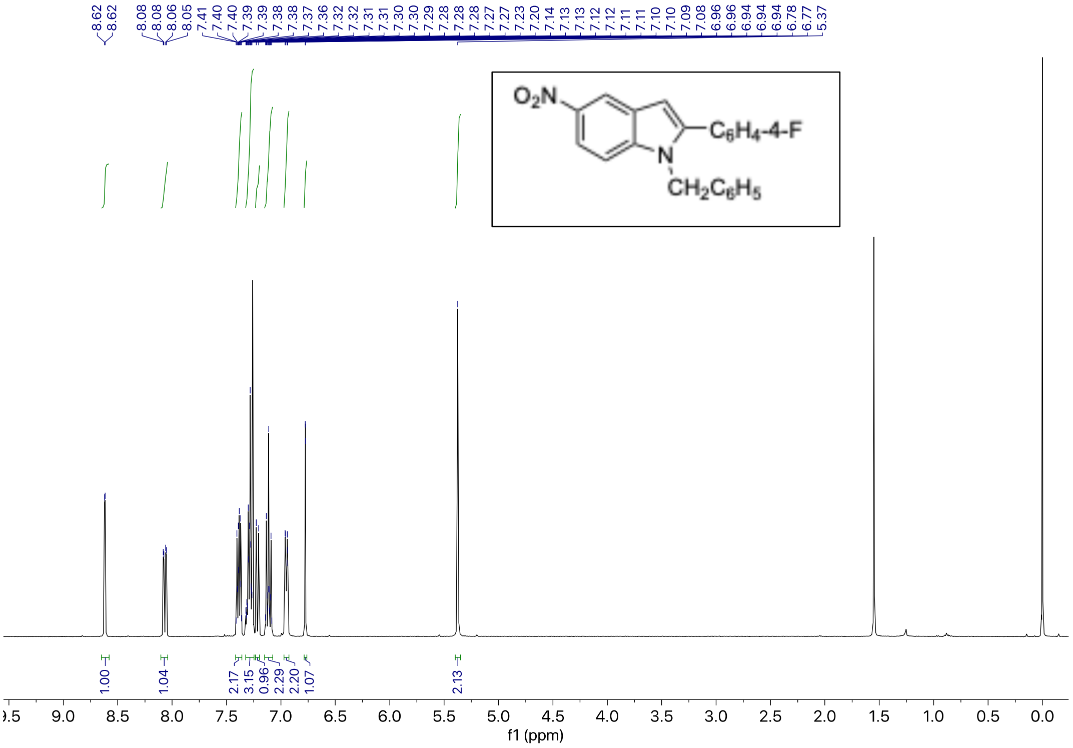

^{13}C NMR of 1-benzyl-2-(4-fluorophenyl)-5-nitro-1*H*-indole (**31**)

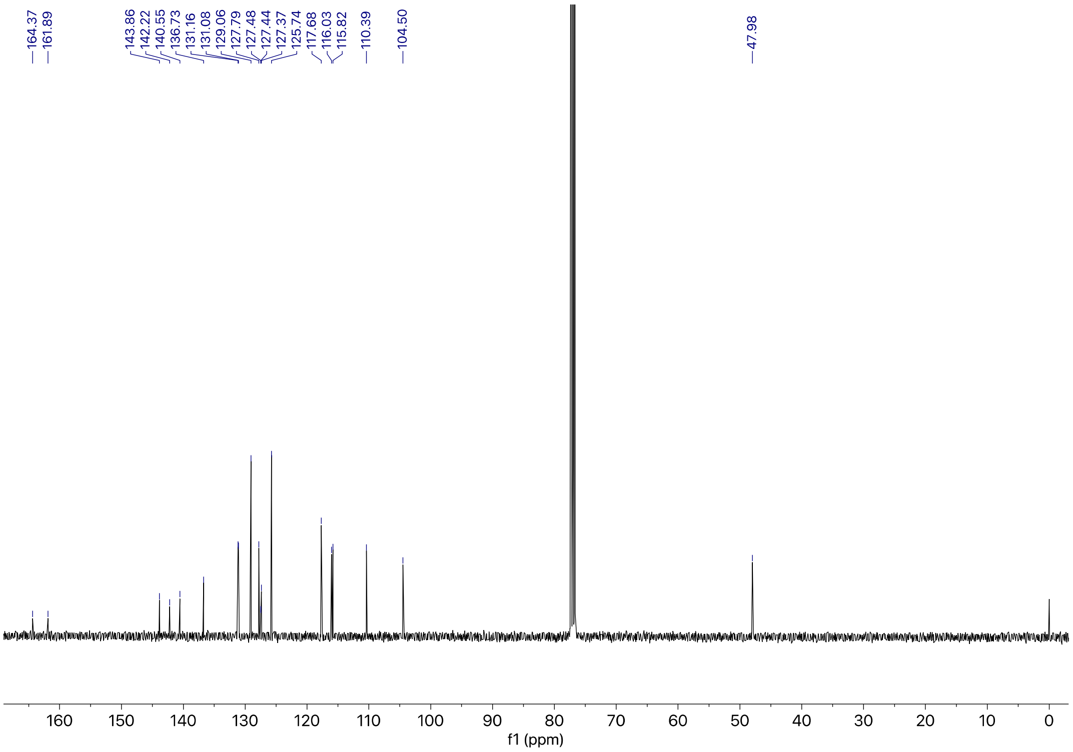

^{19}F NMR of 1-benzyl-2-(4-fluorophenyl)-5-nitro-1*H*-indole (**31**)

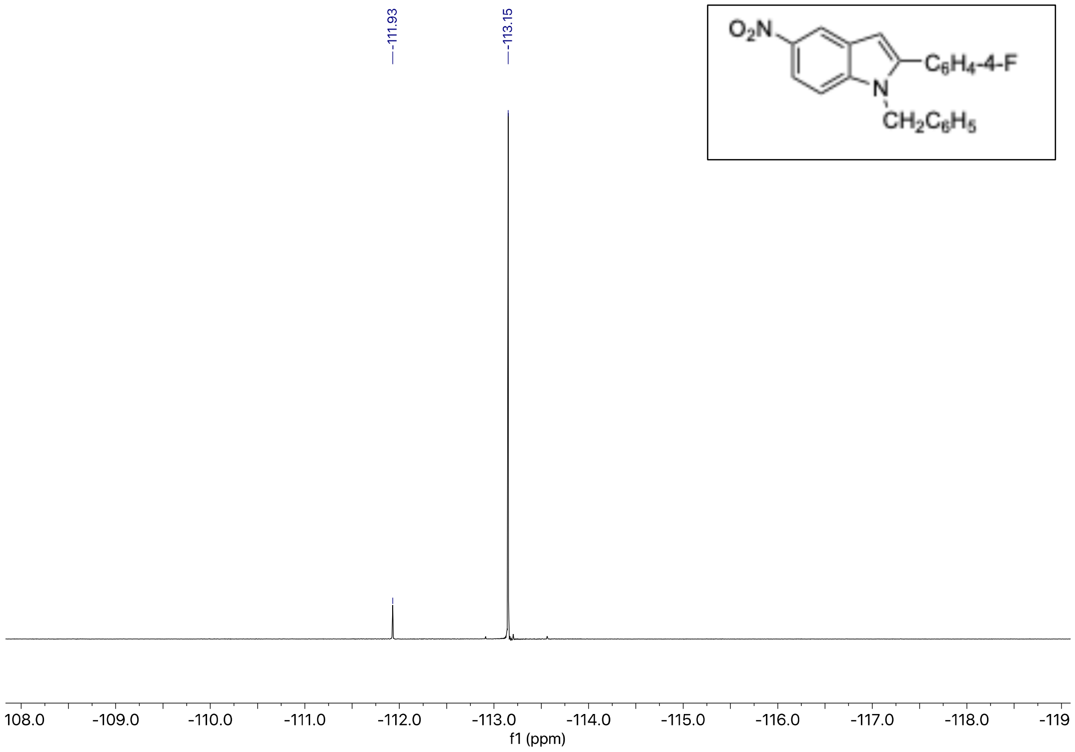

^{1}H NMR of 2-(4-fluorophenyl)-5-nitro-1-(3-(trifluoromethyl)benzyl)-1*H*-indole (**32**)

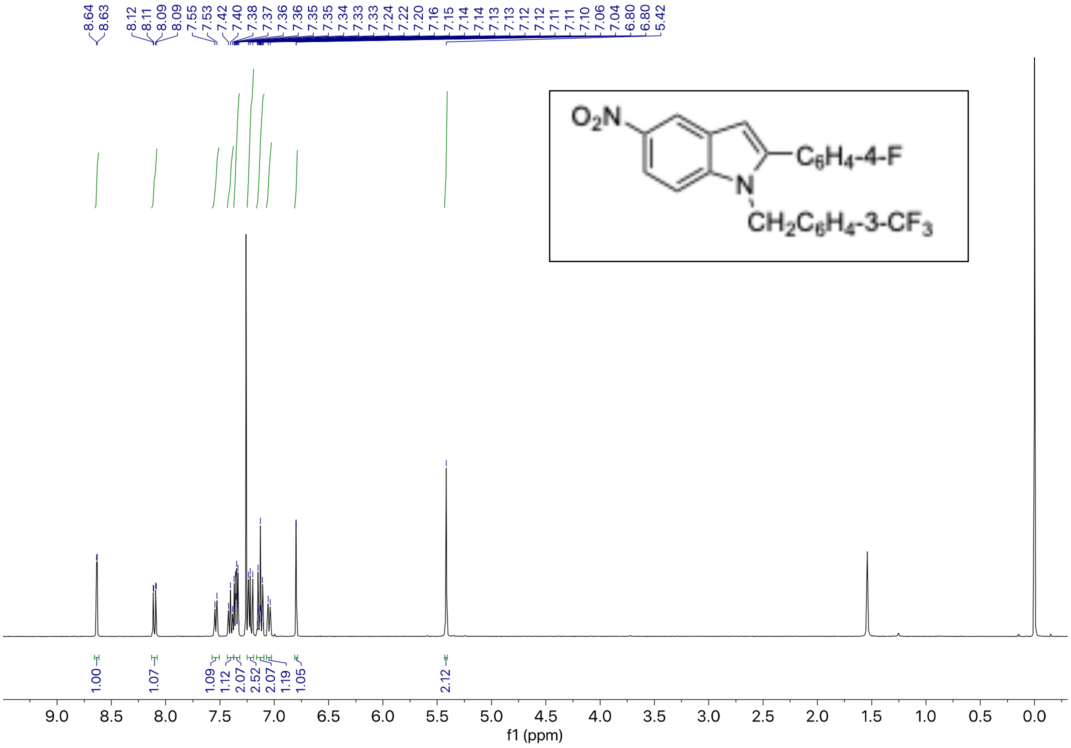

^{13}C NMR of 2-(4-fluorophenyl)-5-nitro-1-(3-(trifluoromethyl)benzyl)-1*H*-indole (**32**)

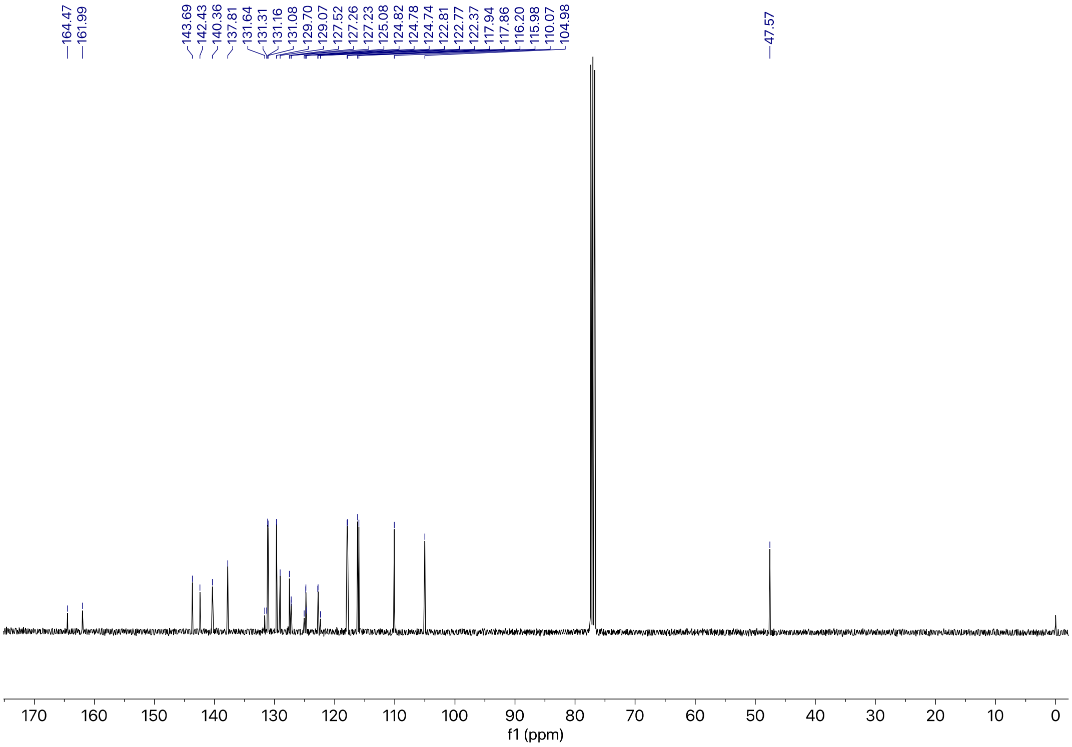

^{19}F NMR of 2-(4-fluorophenyl)-5-nitro-1-(3-(trifluoromethyl)benzyl)-1*H*-indole (**32**)

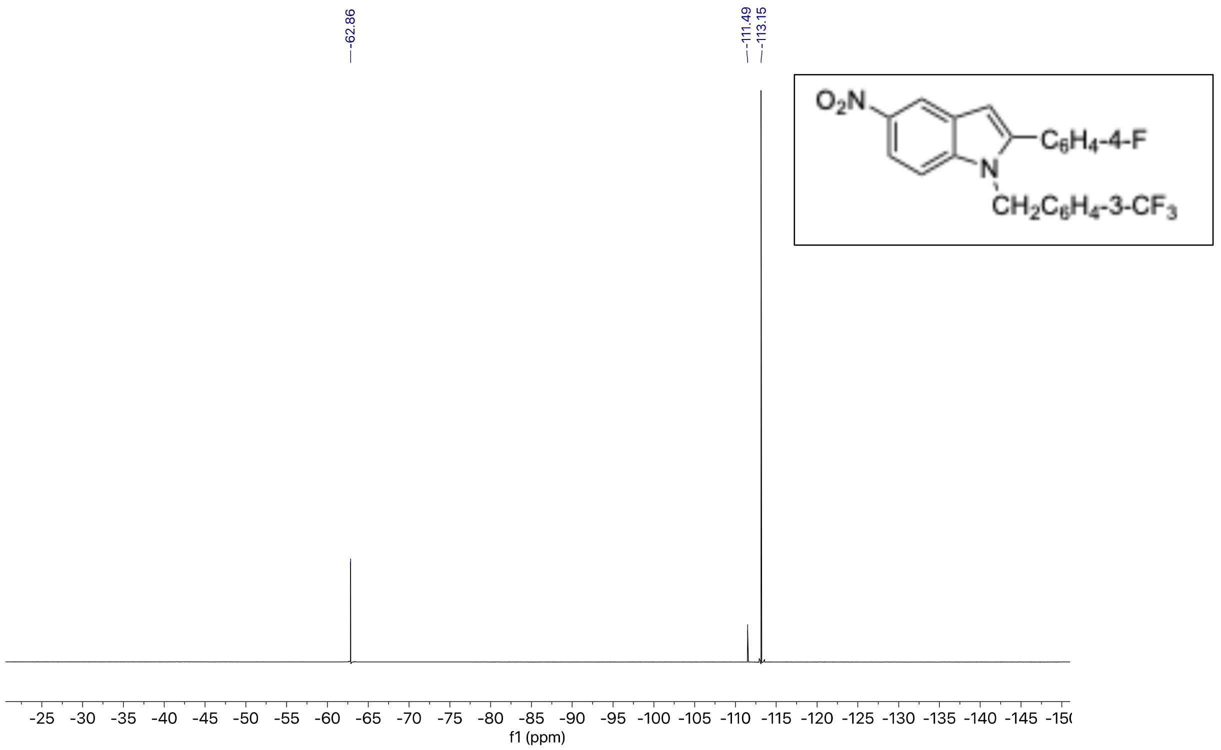

^{1}H NMR of 2-(4-fluorophenyl)-5-nitro-1-phenethyl-1*H*-indole (**33**)

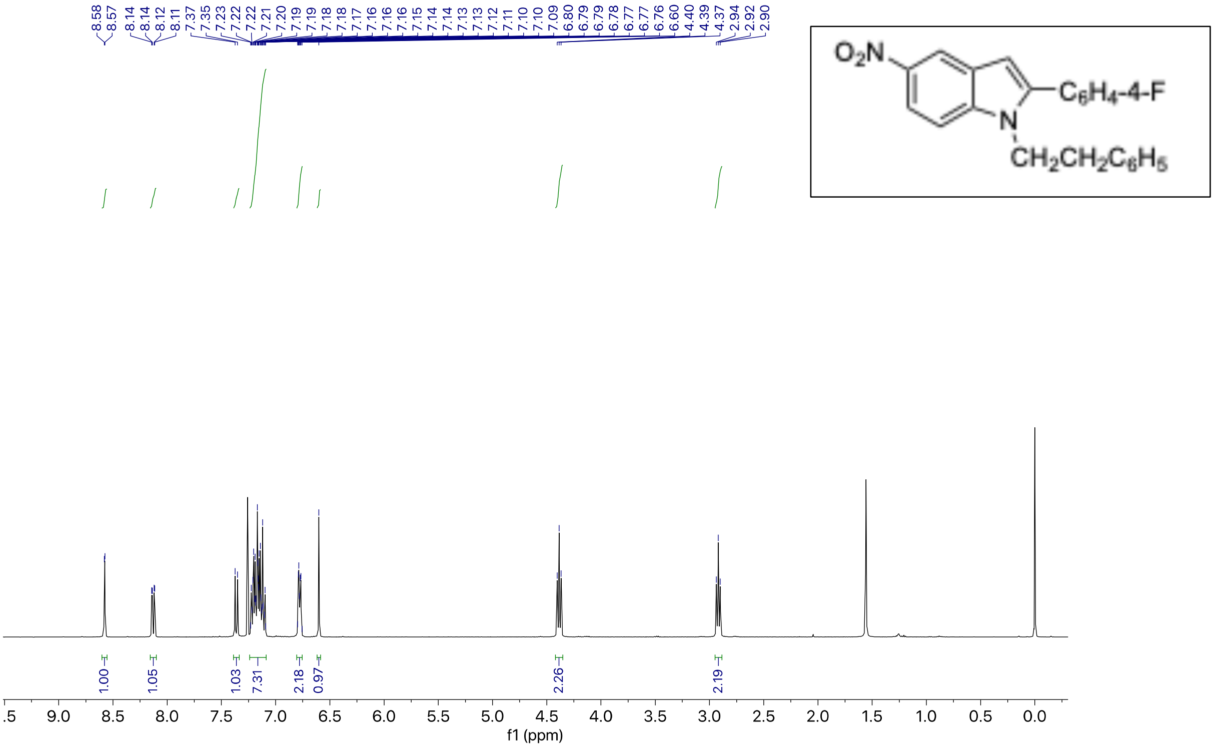

^{13}C NMR of 2-(4-fluorophenyl)-5-nitro-1-phenethyl-1*H*-indole (**33**)

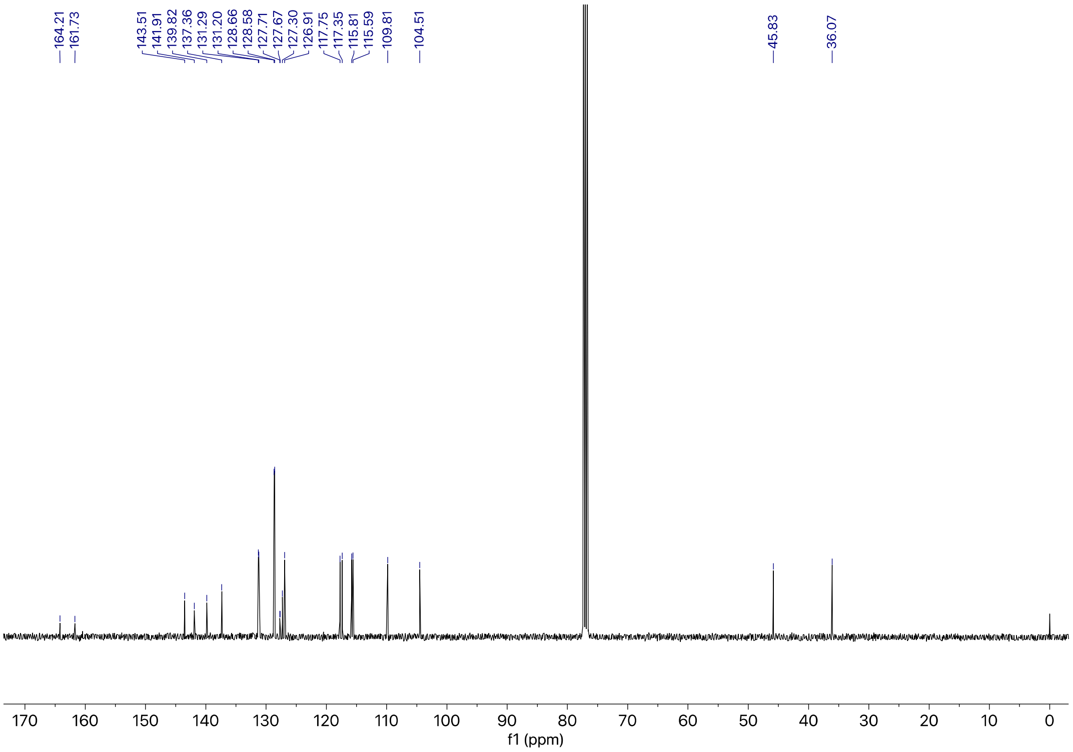

^{19}F NMR of 2-(4-fluorophenyl)-5-nitro-1-phenethyl-1*H*-indole (**33**)

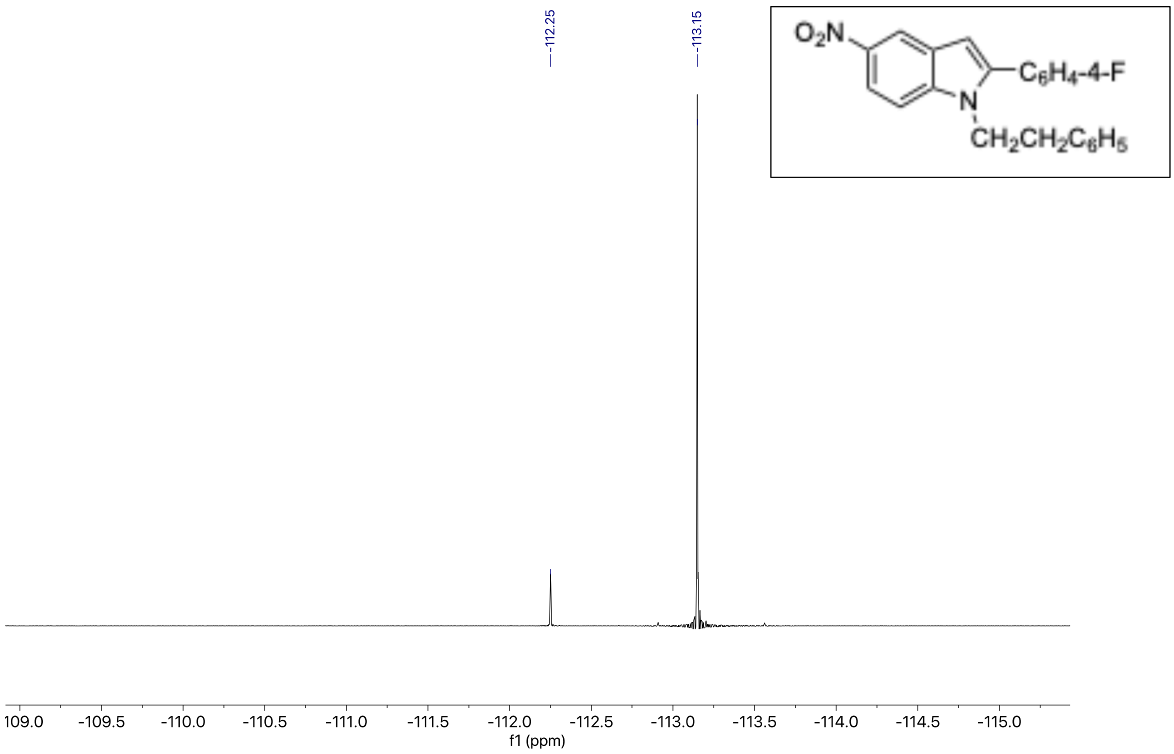

^{1}H NMR of 1-isobutyl-2-(4-fluorophenyl)-5-nitro-1*H*-indole (**34**)

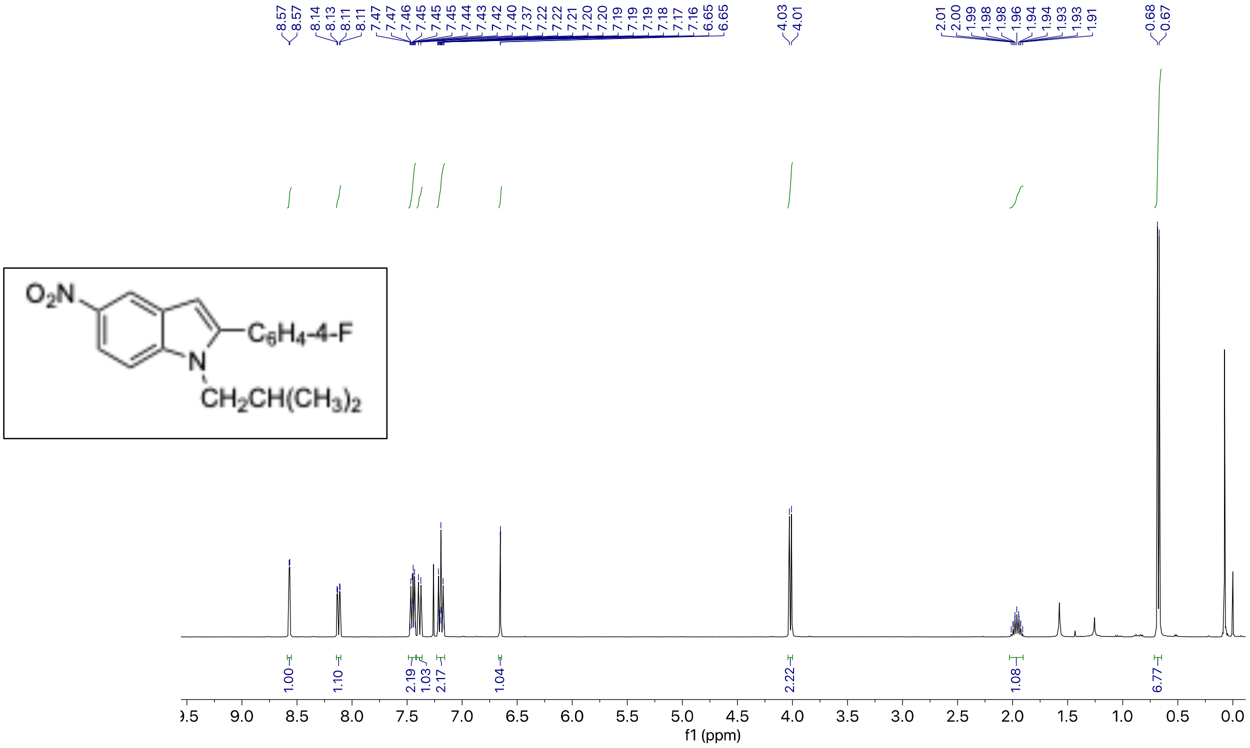

^{13}C NMR of 1-isobutyl-2-(4-fluorophenyl)-5-nitro-1*H*-indole (**34**)

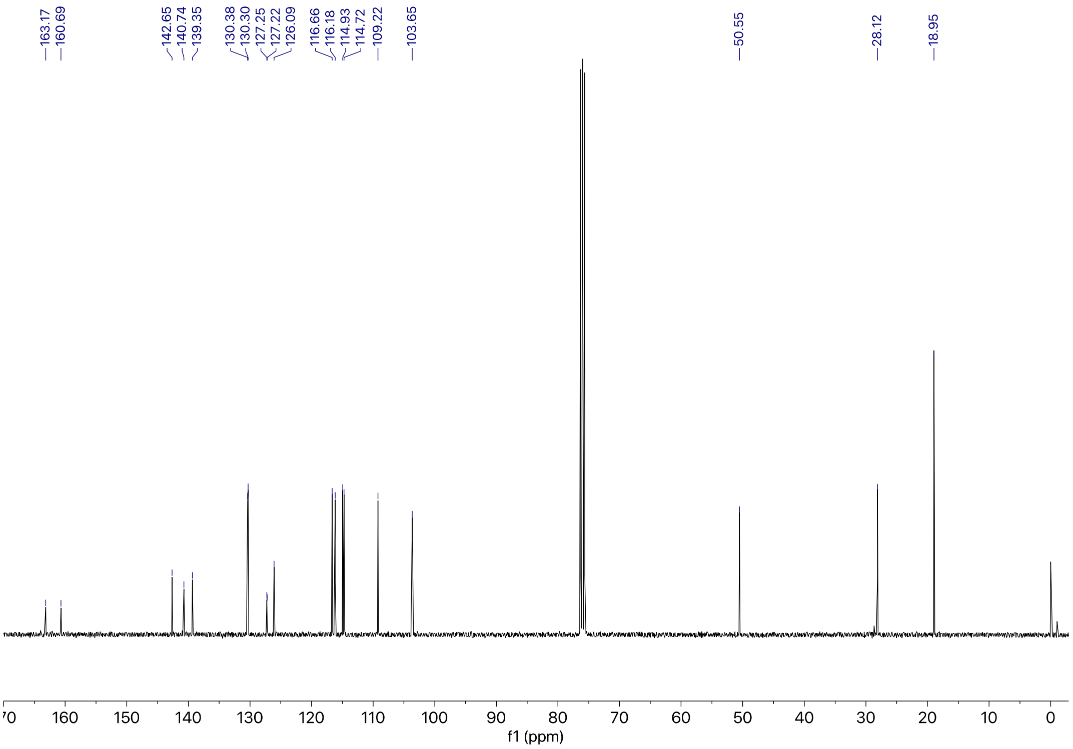

^{19}F NMR of 1-isobutyl-2-(4-fluorophenyl)-5-nitro-1*H*-indole (**34**)

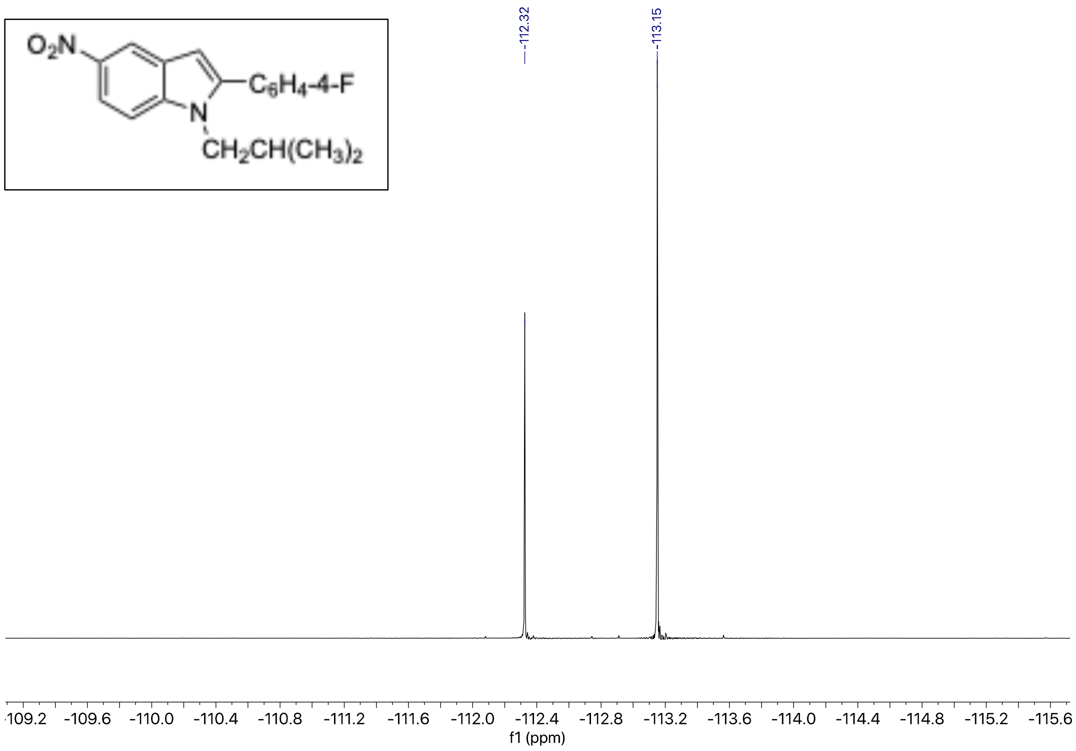

^{1}H NMR of 1-benzyl-2-(4-fluoro-3-methylphenyl)-5-nitro-1*H*-indole (**35**)

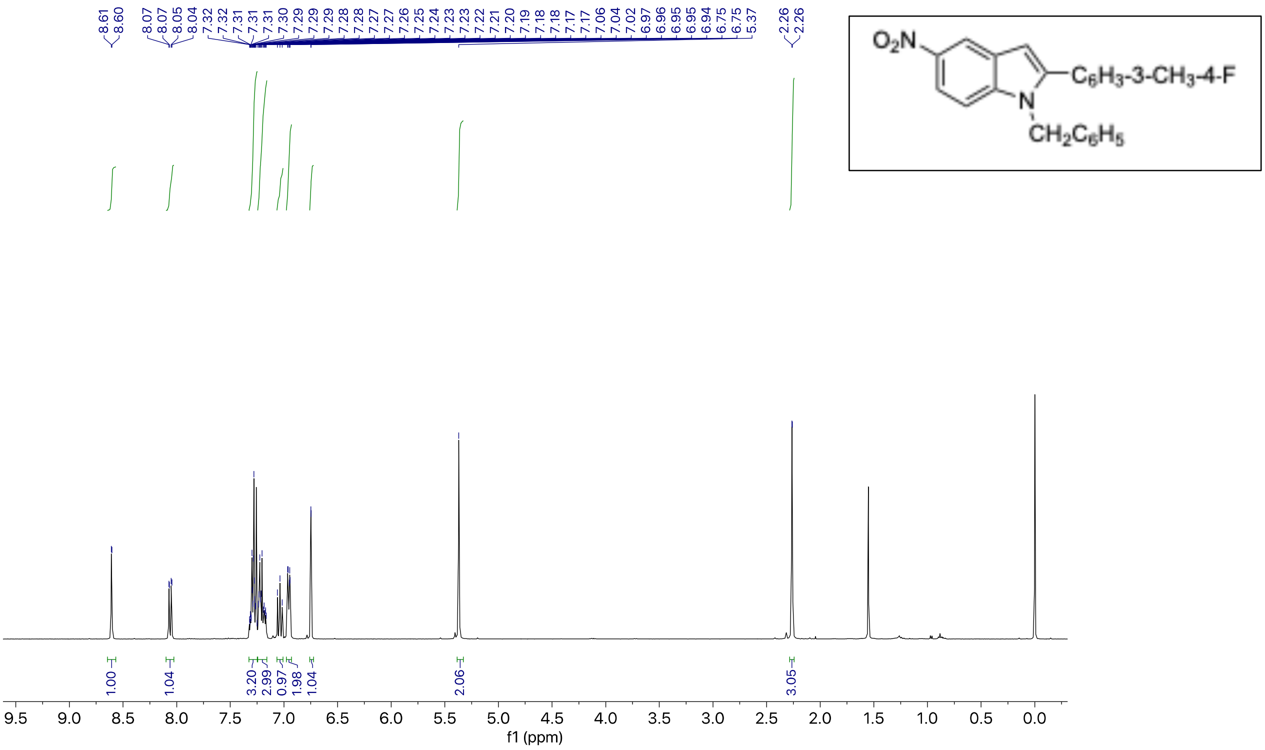

^{13}C NMR of 1-benzyl-2-(4-fluoro-3-methylphenyl)-5-nitro-1*H*-indole (**35**)

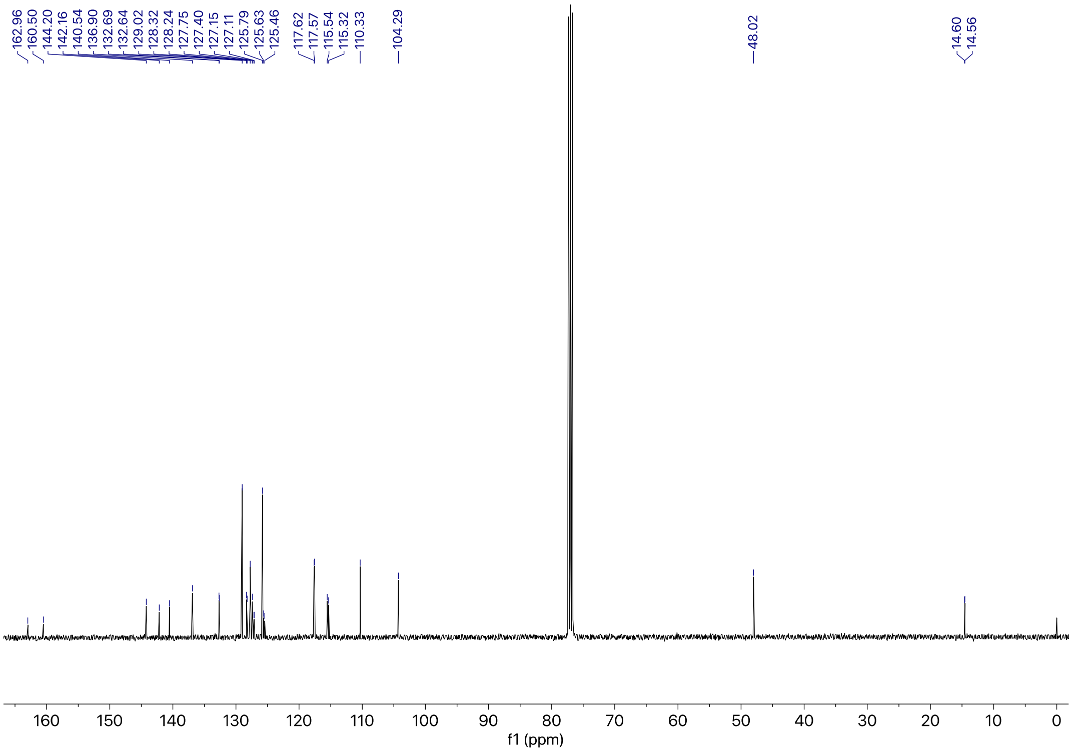

^{19}F NMR of 1-benzyl-2-(4-fluoro-3-methylphenyl)-5-nitro-1*H*-indole (**35**)

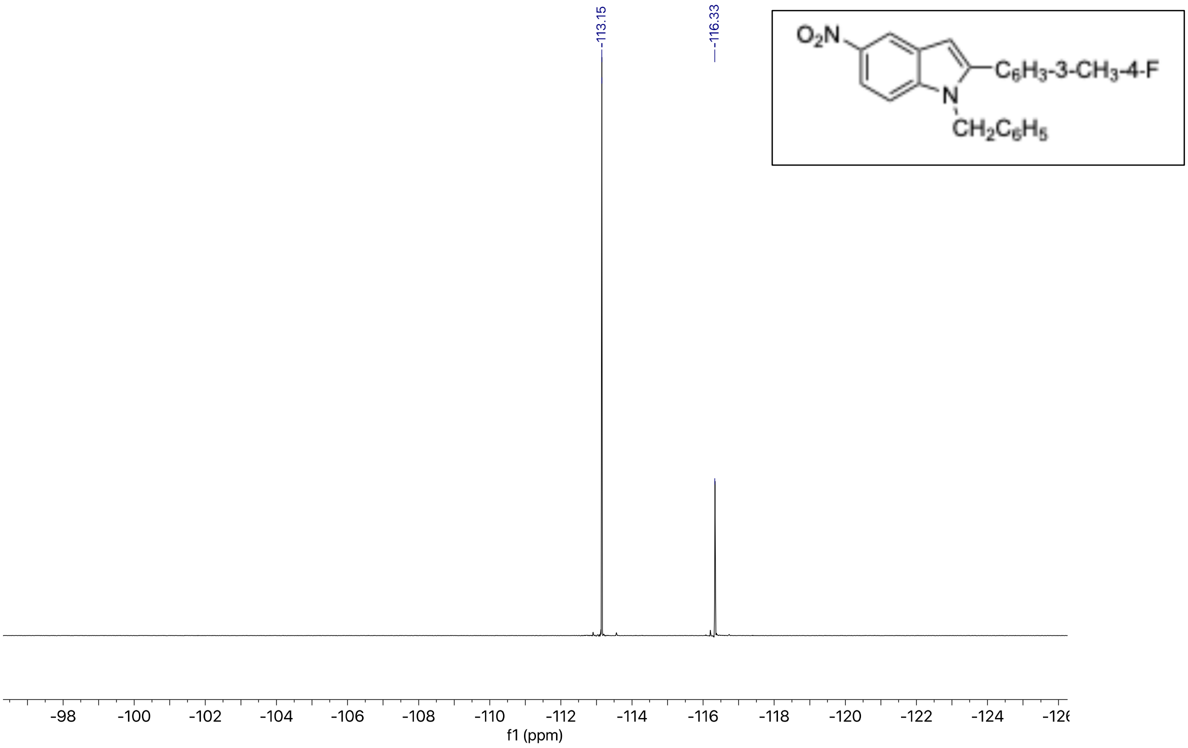

^{1}H NMR of 2-(4-fluoro-3-methylphenyl)-5-nitro-1-phenethyl-1*H*-indole (**36**)

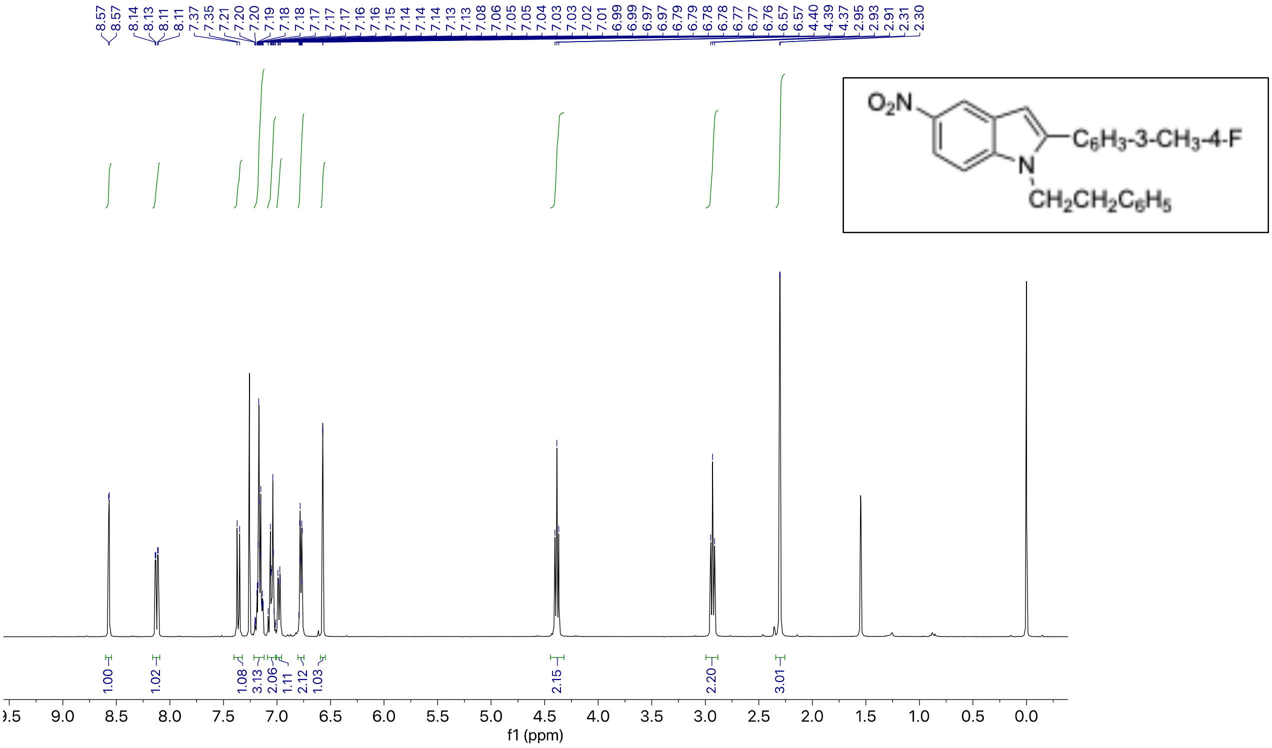

^{13}C NMR of 2-(4-fluoro-3-methylphenyl)-5-nitro-1-phenethyl-1*H*-indole (**36**)

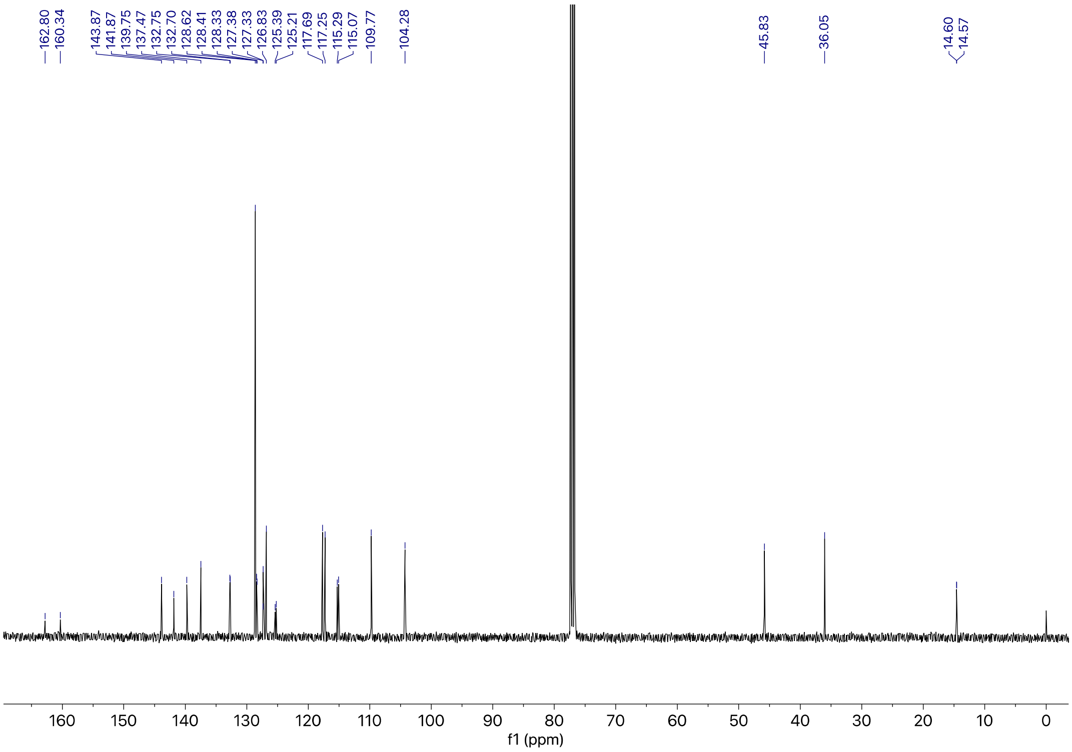

^{9}F NMR of 2-(4-fluoro-3-methylphenyl)-5-nitro-1-phenethyl-1*H*-indole (**36**)

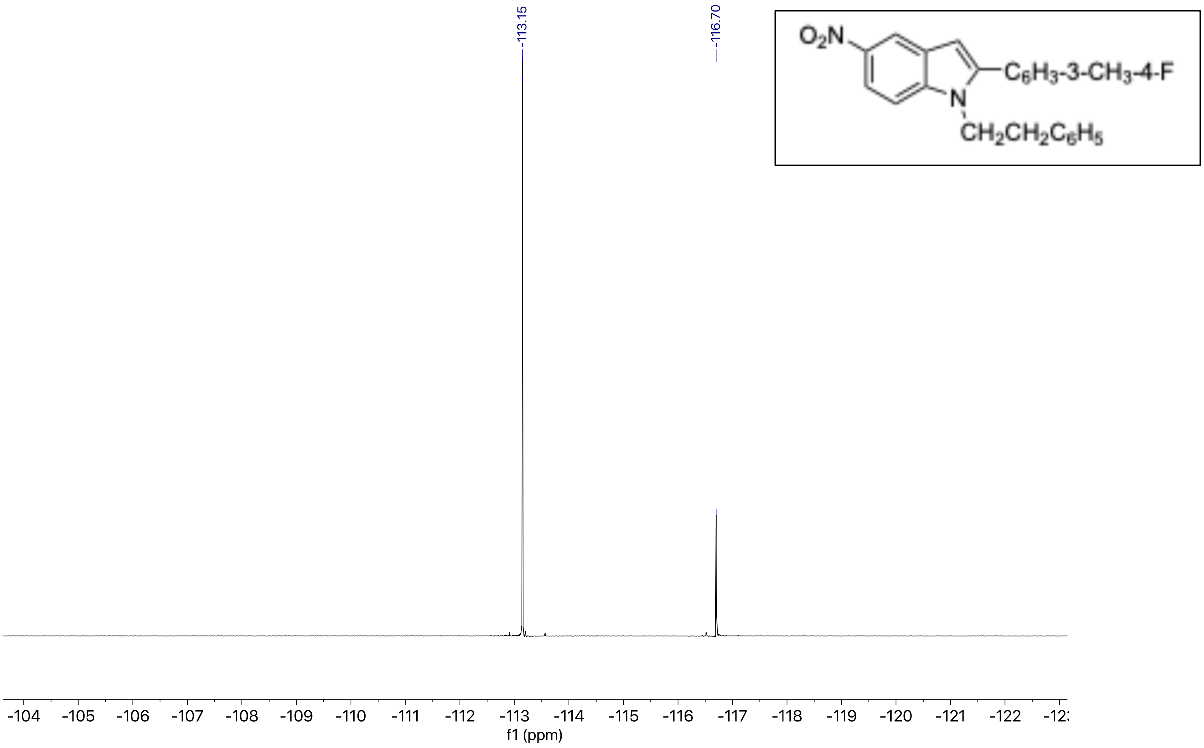

^{1}H NMR of 2-(4-fluoro-3-methylphenyl)-1-(2-fluorophenethyl)-5-nitro-1H-indole (**37**)

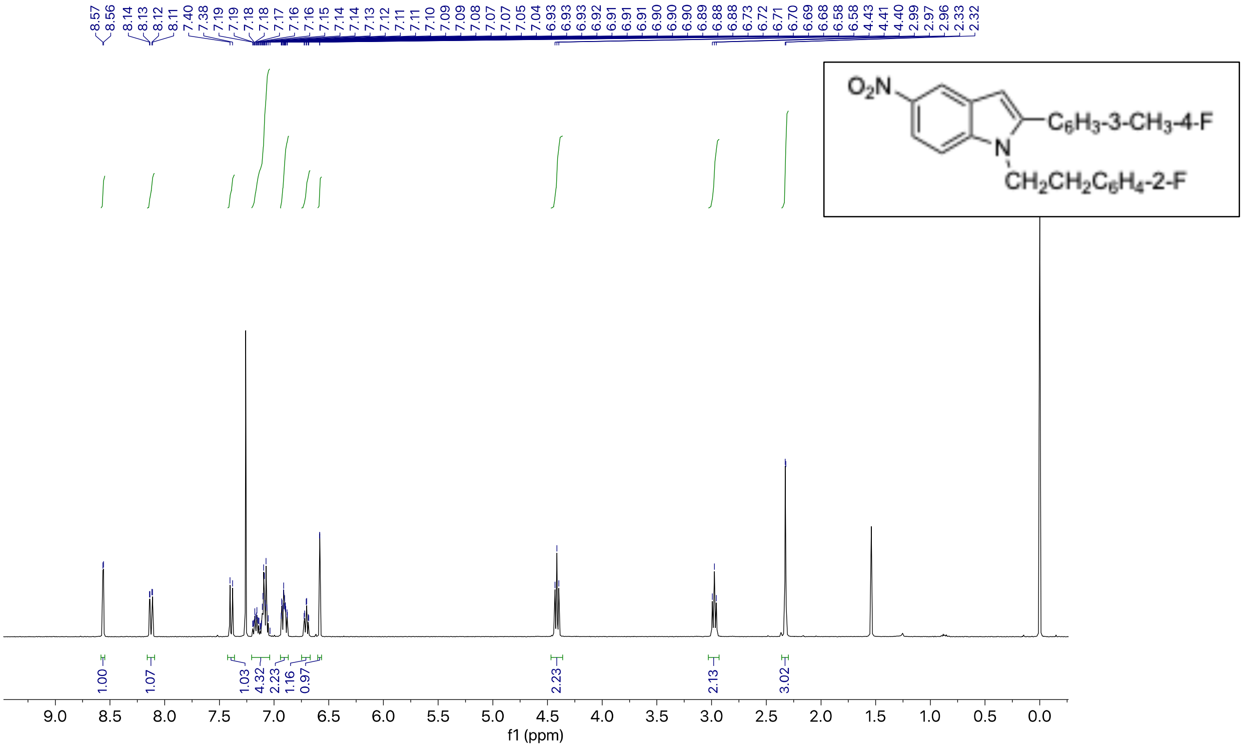

^{13}C NMR of 2-(4-fluoro-3-methylphenyl)-1-(2-fluorophenethyl)-5-nitro-1H-indole (**37**)

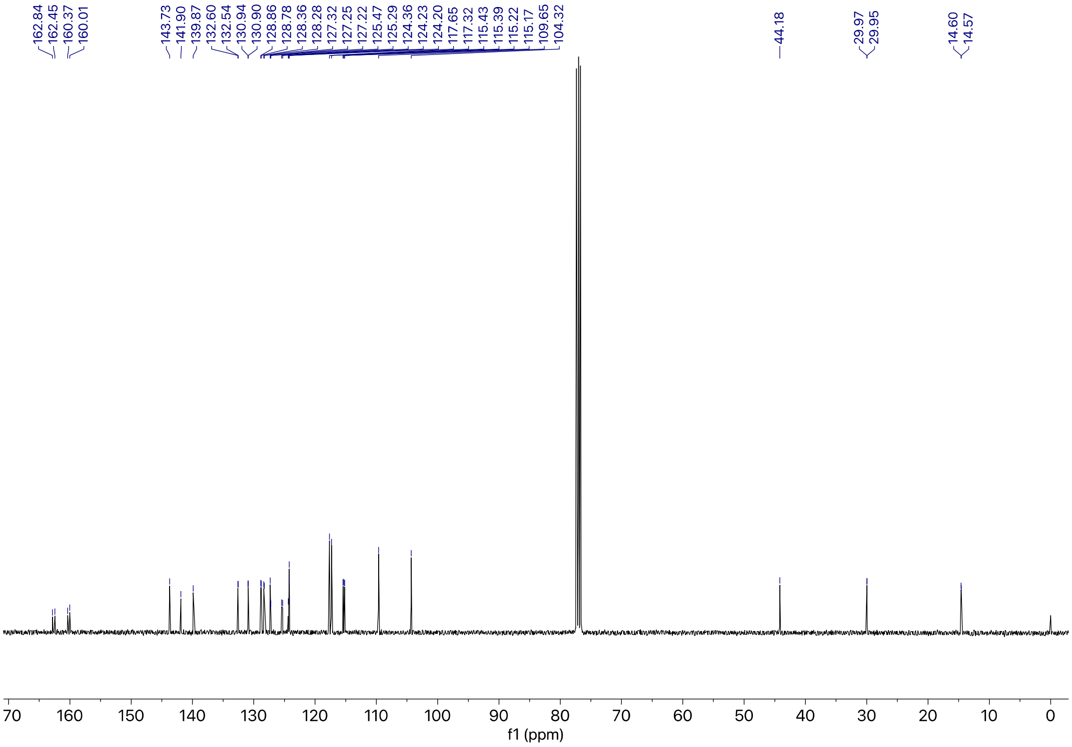

^{19}F NMR of 2-(4-fluoro-3-methylphenyl)-1-(2-fluorophenethyl)-5-nitro-1H-indole (**37**)

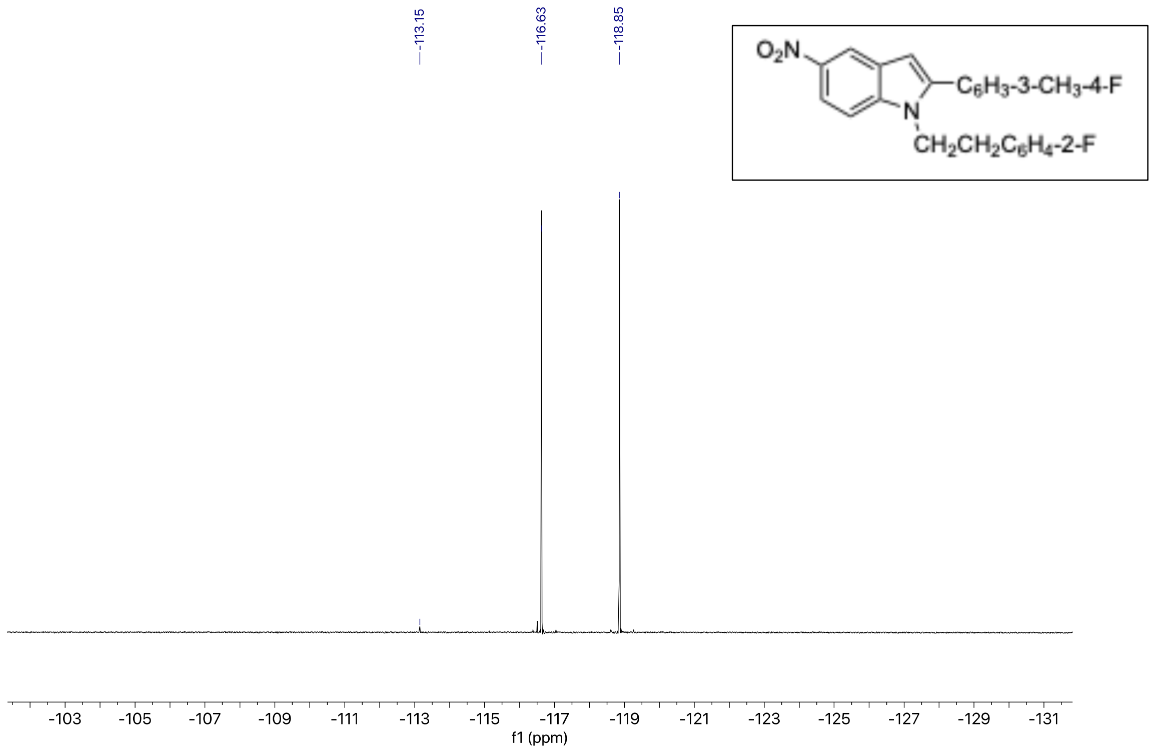

^{1}H NMR of 1-benzyl-2-(4-chlorophenyl)-5-nitro-1*H*-indole (**38**)

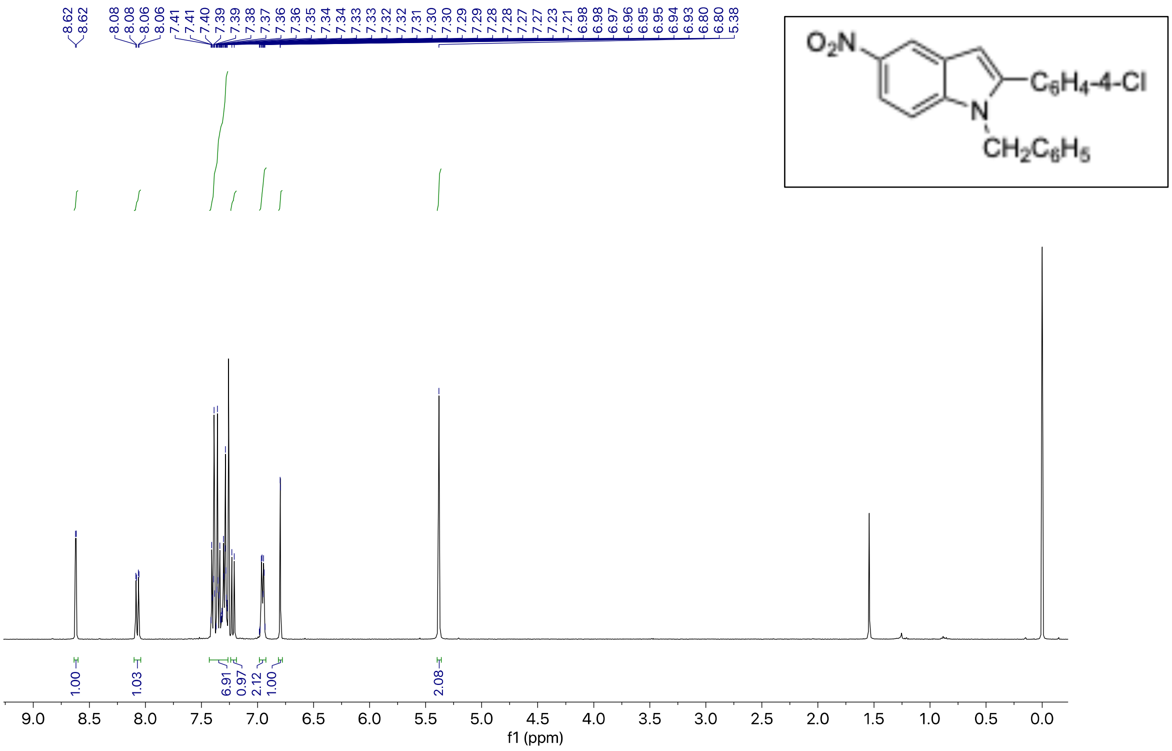

13C NMR of 1-benzyl-2-(4-chlorophenyl)-5-nitro-1*H*-indole (**38**)

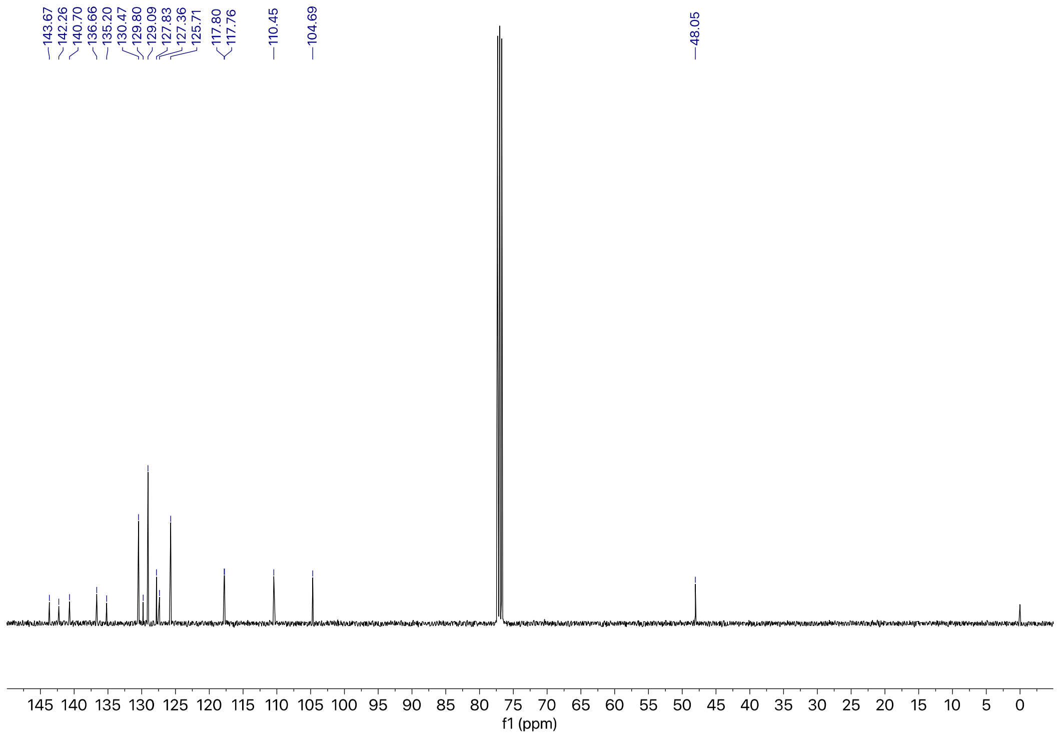

1H NMR of 2-(4-chlorophenyl)-5-nitro-1-(3-(trifluoromethyl)benzyl)-1*H*-indole (**39**)

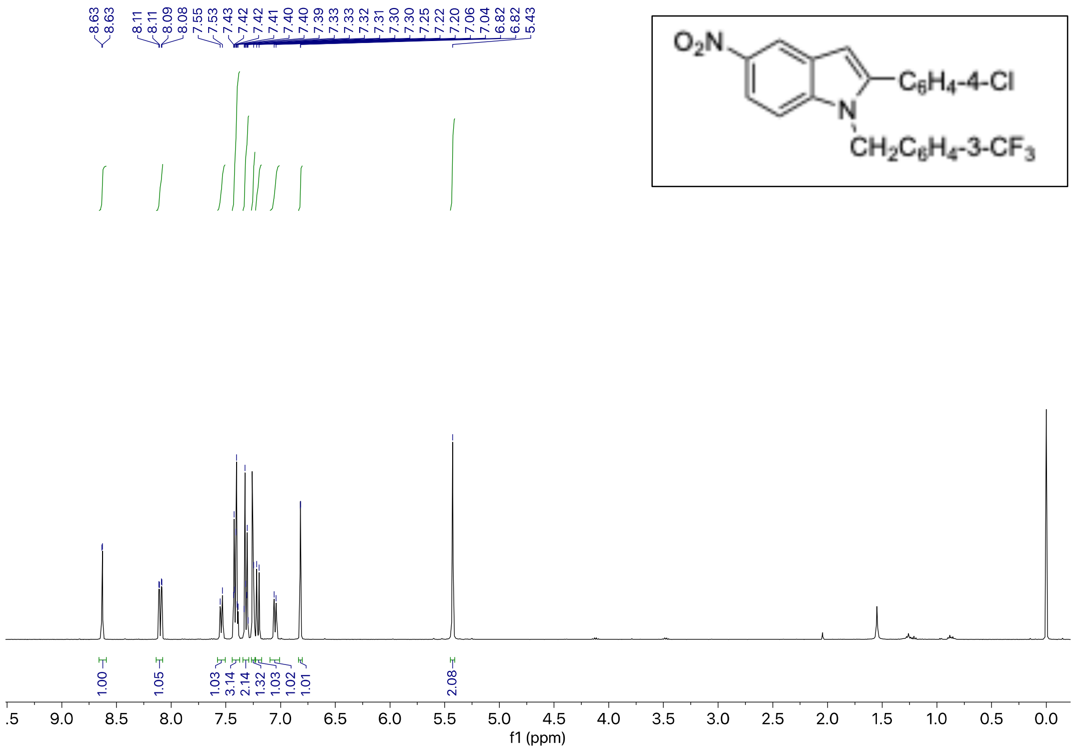

^{13}C NMR of 2-(4-chlorophenyl)-5-nitro-1-(3-(trifluoromethyl)benzyl)-1*H*-indole (**39**)

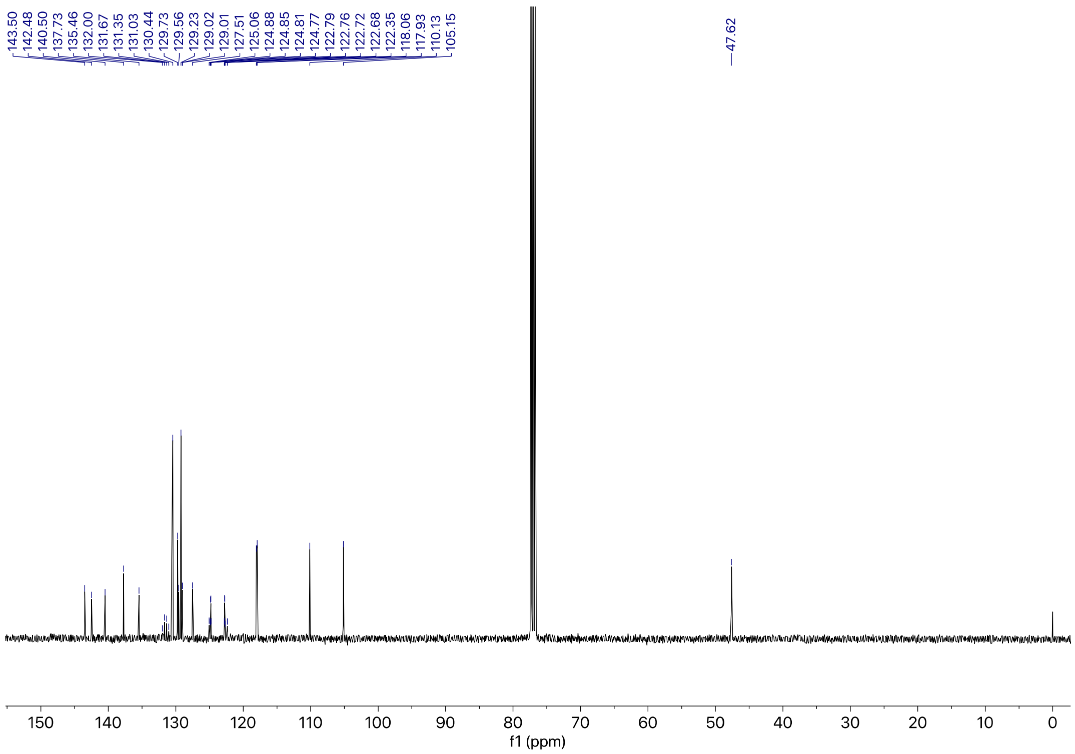

^{19}F NMR of 2-(4-chlorophenyl)-5-nitro-1-(3-(trifluoromethyl)benzyl)-1*H*-indole (**39**)

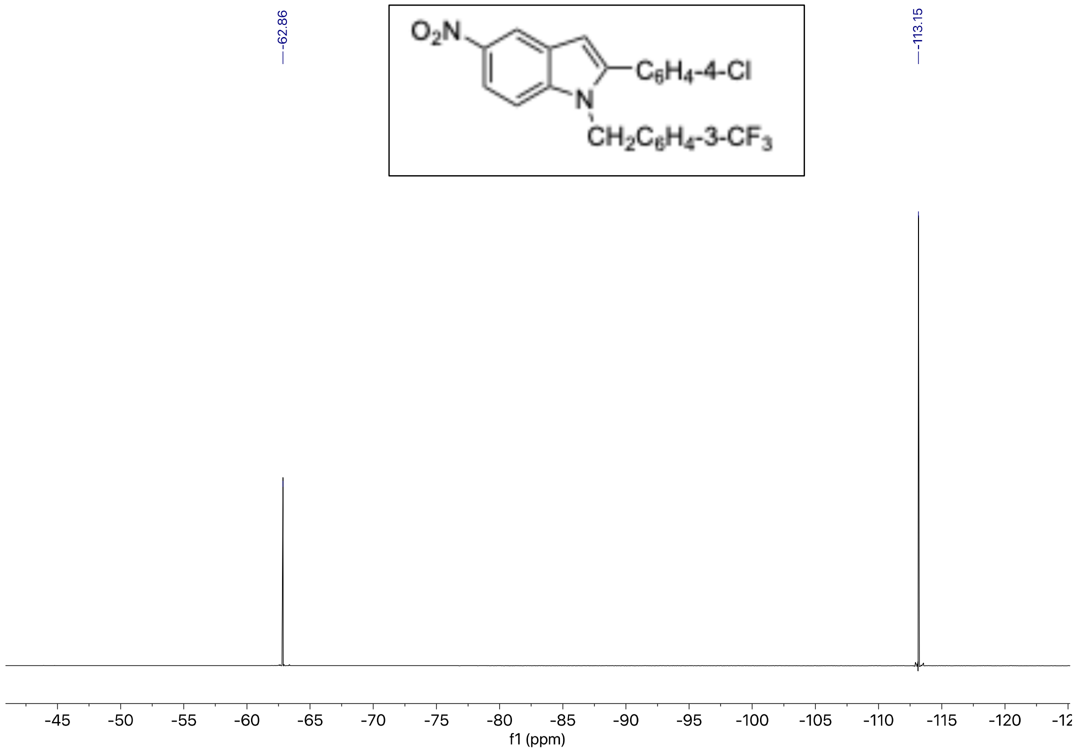

^{1}H NMR of 2-(4-chlorophenyl)-5-nitro-1-phenethyl-1*H*-indole (**40**)

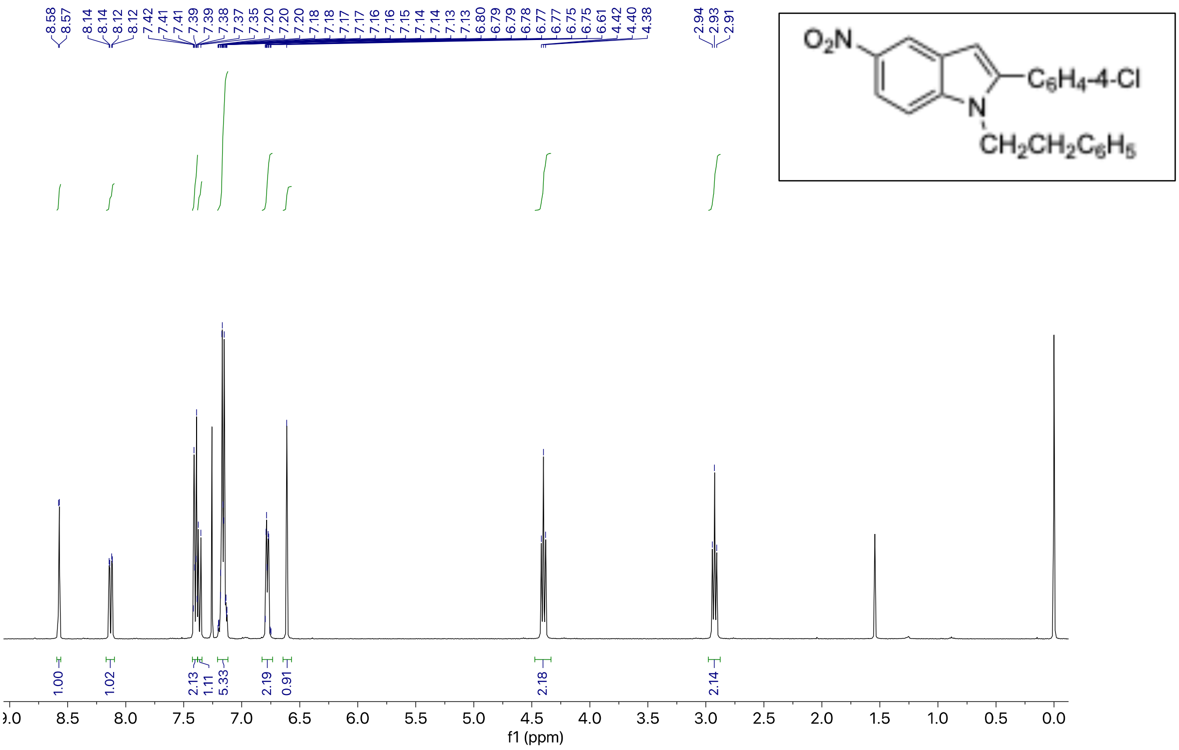

13C NMR of 2-(4-chlorophenyl)-5-nitro-1-phenethyl-1*H*-indole (**40**)

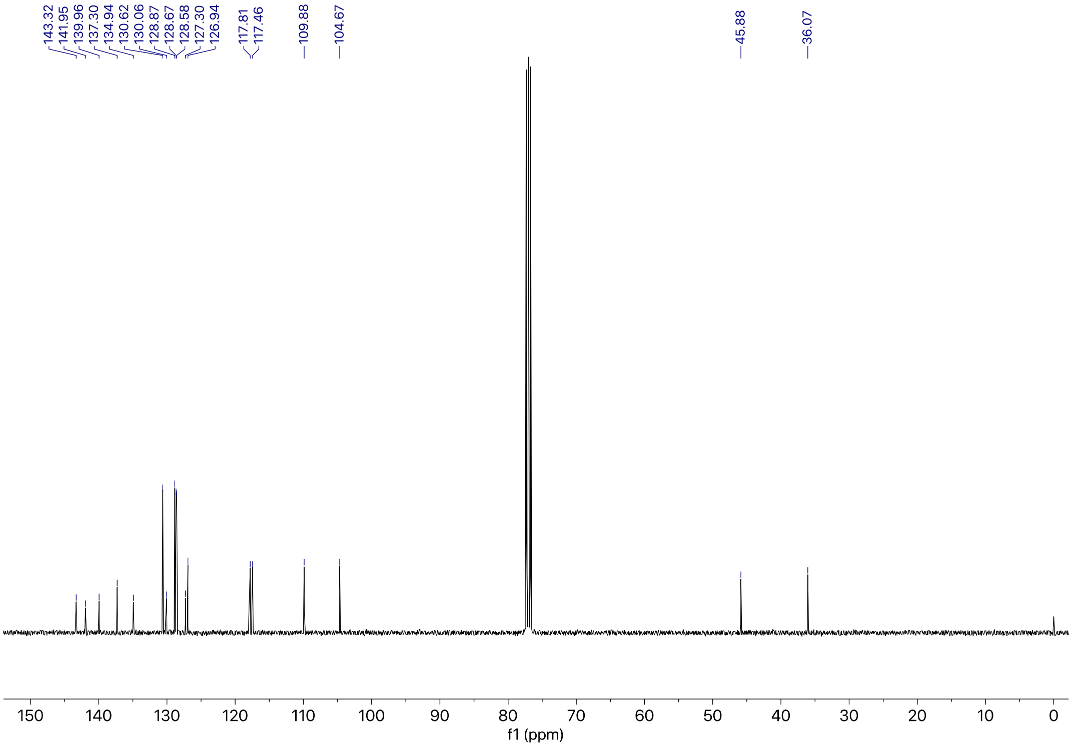

1H NMR of 1-benzyl-2-(2,3-dihydrobenzo[*b*][1,4]dioxin-6-yl-)-5-nitro-1*H*-indole (**41**)

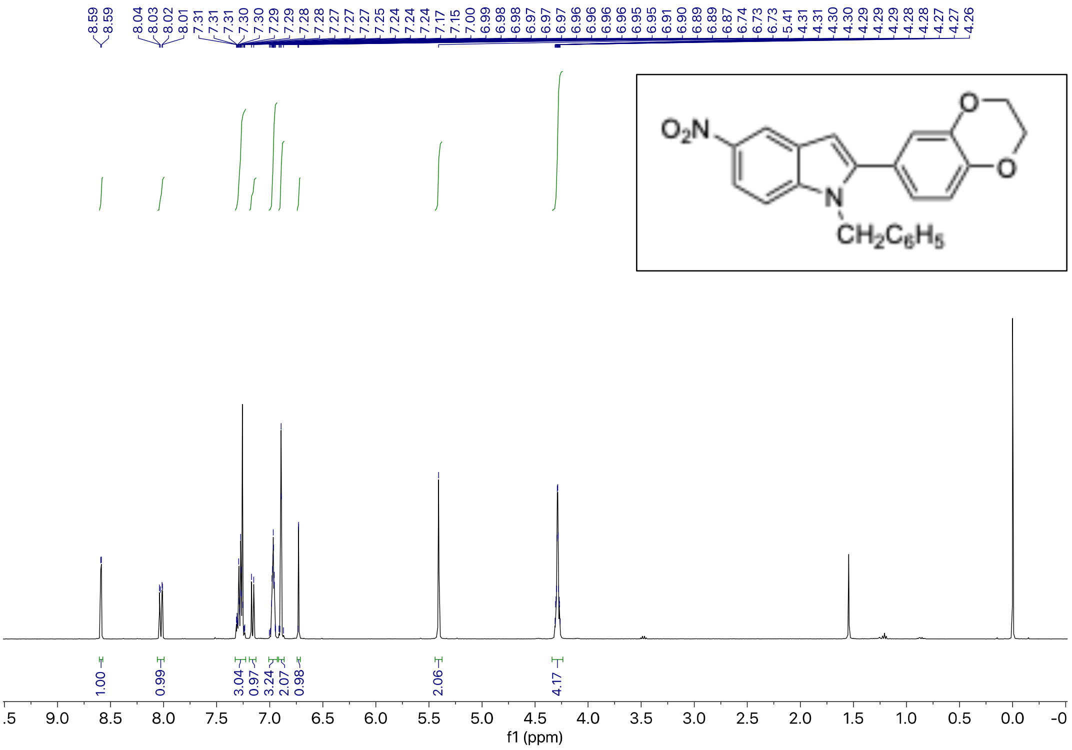

^{13}C NMR of 1-benzyl-2-(2,3-dihydrobenzo[*b*][1,4]dioxin-6-yl-)-5-nitro-1*H*-indole (**41**)

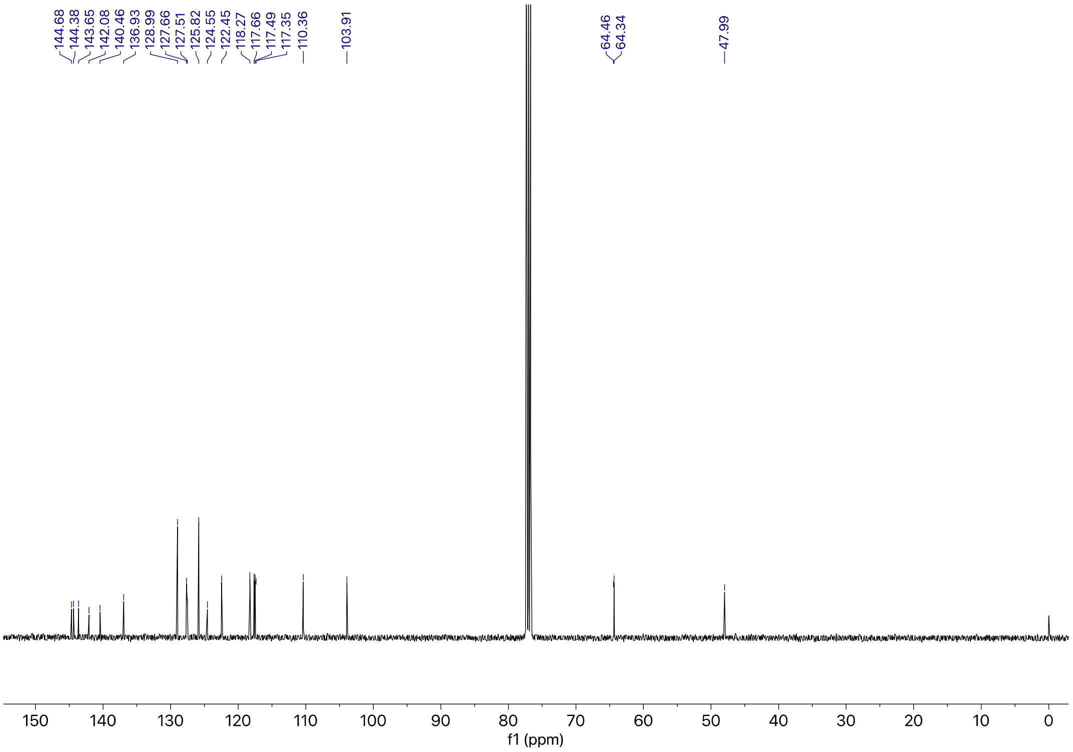

^{1}H NMR of 2-(2,3-dihydobenzo[*b*][1,4]dioxin-6-yl)-1-(2-fluorophenyl)-5-nitro-1*H*-indole (**42**)

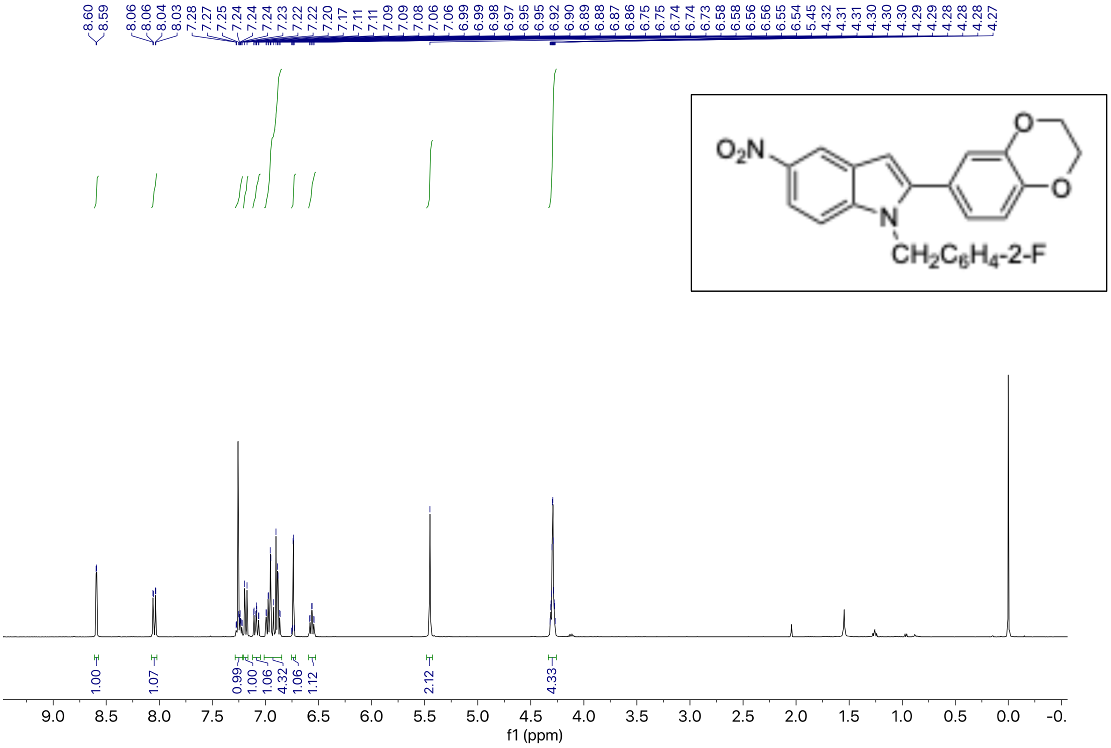

^{13}C NMR of 2-(2,3-dihydobenzo[*b*][1,4]dioxin-6-yl)-1-(2-fluorophenyl)-5-nitro-1*H*-indole (**42**)

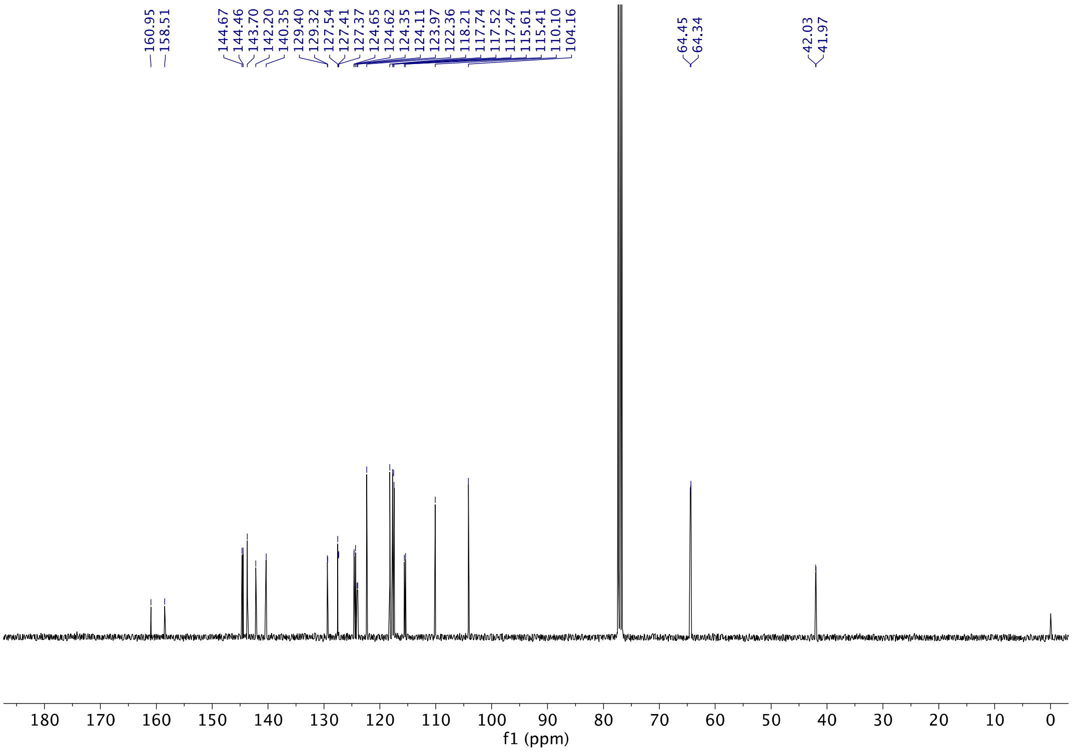

^{19}F NMR of 2-(2,3-dihydobenzo[*b*][1,4]dioxin-6-yl)-1-(2-fluorophenyl)-5-nitro-1*H*-indole (**42**)

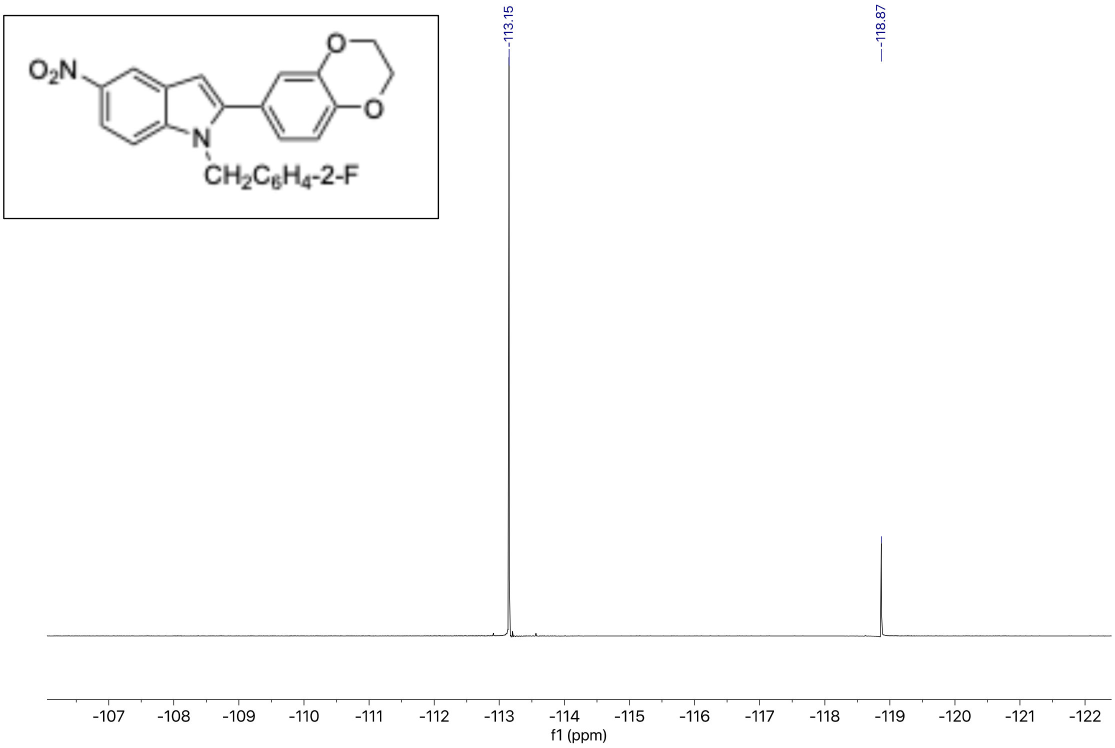

^{1}H NMR of 2-(2,3-dihydobenzo[*b*][1,4]dioxin-6-yl)-5-nitro-1-phenethyl-1*H*-indole (**43**)

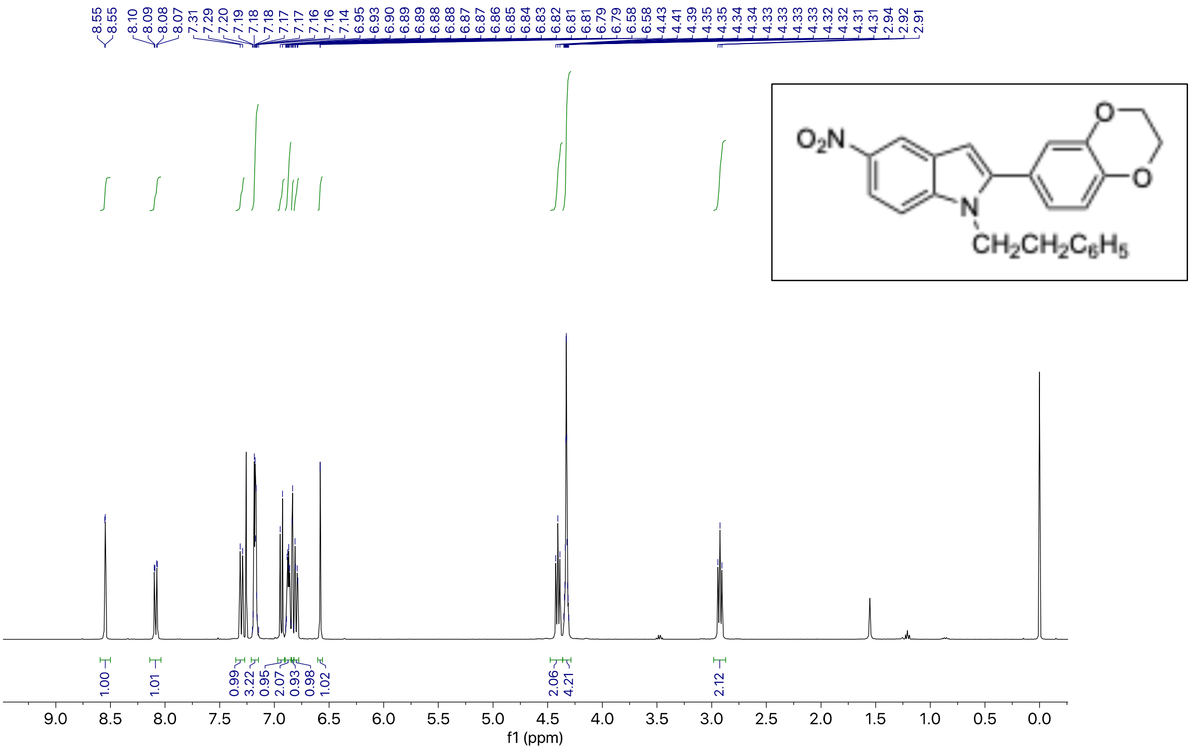

^{13}C NMR of 2-(2,3-dihydobenzo[*b*][1,4]dioxin-6-yl)-5-nitro-1-phenethyl-1*H*-indole (**43**)

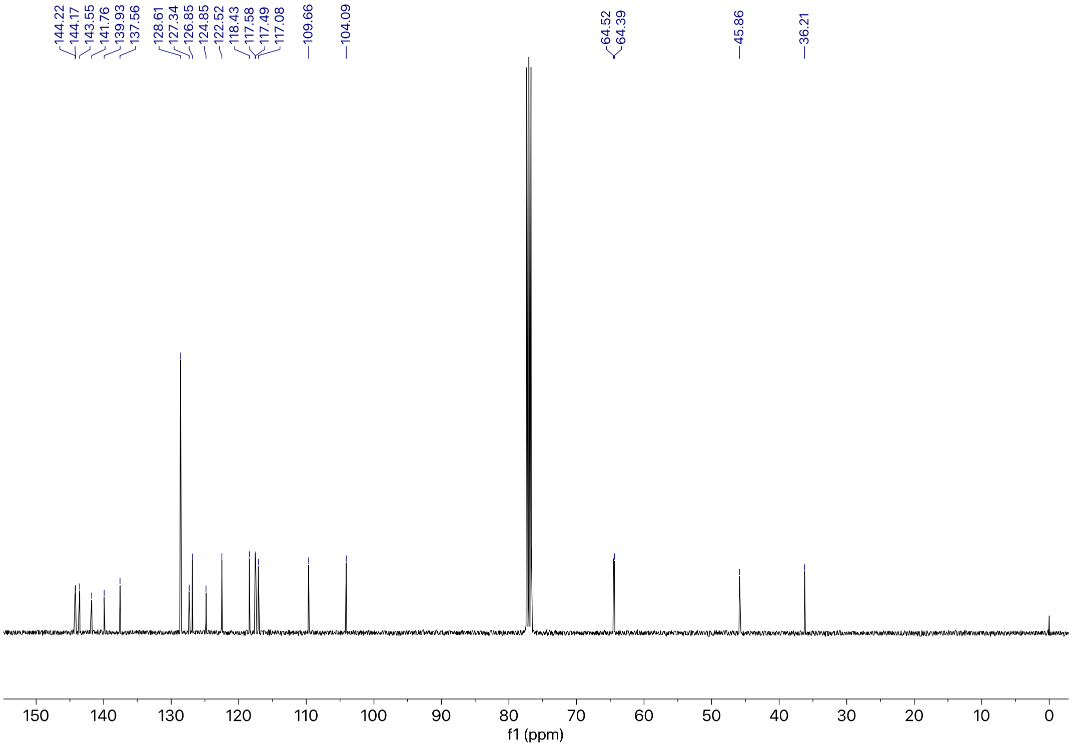

Supplement: Supplementary file 1 [file molecules-30-03894-s001.zip › molecules-3877273-supplementary.pdf]
